# Photoredox catalysis on unactivated substrates with strongly reducing iridium photosensitizers†

**DOI:** 10.1039/d0sc06306a

**Published:** 2021-01-29

**Authors:** Jong-Hwa Shon, Dooyoung Kim, Manjula D. Rathnayake, Steven Sittel, Jimmie Weaver, Thomas S. Teets

**Affiliations:** Department of Chemistry, University of Houston 3585 Cullen Blvd., Room 112 Houston TX 77204-5003 USA tteets@uh.edu; Department of Chemistry, Oklahoma State University 107, Physical Science Stillwater OK 74078 USA

## Abstract

Photoredox catalysis has emerged as a powerful strategy in synthetic organic chemistry, but substrates that are difficult to reduce either require complex reaction conditions or are not amenable at all to photoredox transformations. In this work, we show that strong bis-cyclometalated iridium photoreductants with electron-rich β-diketiminate (NacNac) ancillary ligands enable high-yielding photoredox transformations of challenging substrates with very simple reaction conditions that require only a single sacrificial reagent. Using blue or green visible-light activation we demonstrate a variety of reactions, which include hydrodehalogenation, cyclization, intramolecular radical addition, and prenylation *via* radical-mediated pathways, with optimized conditions that only require the photocatalyst and a sacrificial reductant/hydrogen atom donor. Many of these reactions involve organobromide and organochloride substrates which in the past have had limited utility in photoredox catalysis. This work paves the way for the continued expansion of the substrate scope in photoredox catalysis.

## Abstract 2

Strong bis-cyclometalated iridium photoreductants, in combination with a single sacrificial reductant, enable visible-light-promoted reductive activation of a variety of challenging substrates under simple and general reaction conditions.

## Graphical abstract

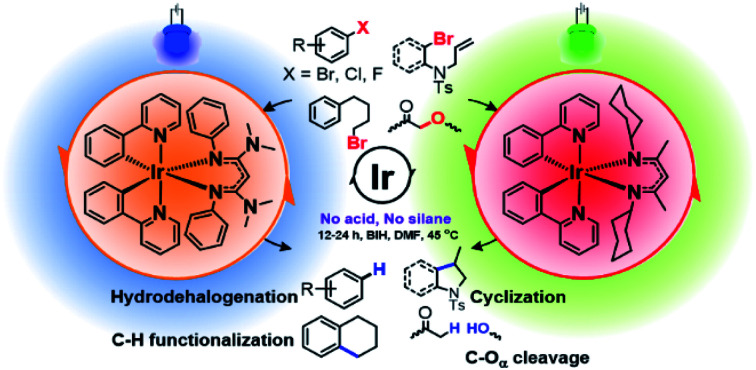

## Introduction

Visible-light photoredox catalysis has emerged as a powerful, versatile, and increasingly important methodology in organic synthesis.^1^ In this strategy, a photosensitizer, usually a chromophoric organic compound or transition-metal coordination complex, absorbs visible light and initiates charge transfer to the substrate, either directly, *via* a redox mediator, or with the assistance of a co-catalyst. Such an approach had already become mainstream in the area of solar fuels,^2,3^ before being adopted by the small-molecule organic synthesis community and even more recently becoming widespread in polymer synthesis.^4^

One of the most significant remaining challenges in photoredox catalysis is to extend the substrate scope to molecules that are difficult to reduce or oxidize. In photoredox transformations initiated by electron transfer, highly activated substrates, like α-carbonyl halides, benzyl halides, sulfones, and sulfoniums are commonly used.^5–8^ These substrate classes can be readily activated by homoleptic ruthenium photosensitizers of the general formula [Ru(*N*^*N*)_3_]^2+^, where *N*^*N* is 2,2′-bipyridine or a related derivative, as well as certain organic photosensitizers. To expand the substrate scope to molecules that are more difficult to reduce, Stephenson's group turned to another photosensitizer which has become ubiquitous in photoredox catalysis, *fac*-Ir(ppy)_3_ (ppy = 2-phenylpyridine),^9^ demonstrating hydrodeiodination of unactivated aryl, alkyl, and vinyl iodide substrates.^10^ This photosensitizer has an excited-state reduction potential almost 1 V more negative than the common ruthenium polypyridyl photosensitizers, and has become a very popular choice for photoredox methodology involving less activated substrates.^10,11^ It is also possible to activate the same substrate classes for cyclization reactions, using [Ir(ppy)_2_(dtbbpy)]^+^ (dtbbpy = 4,4′-di-*tert*-butyl-2,2′-bipyridine) as the photosensitizer.^12^

**Fig. 1:**
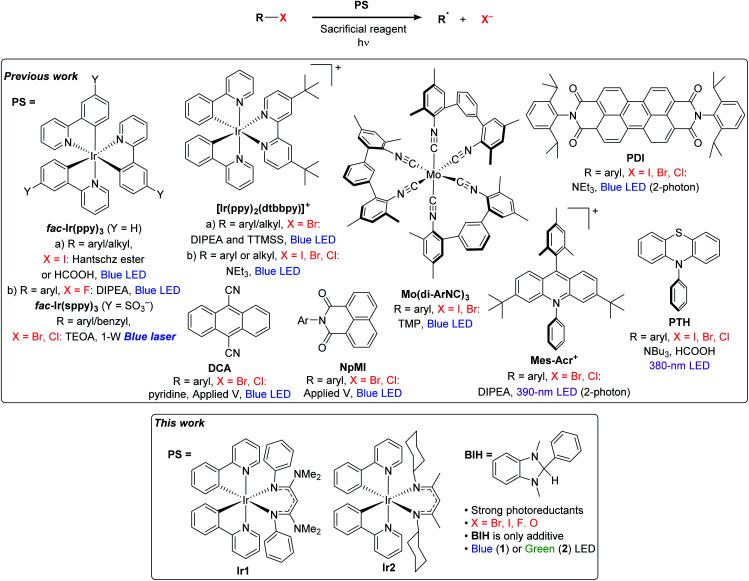
Previously reported methods for photoredox activation of challenging substrates and an introduction to this work.

Despite the recent successes expanding photoredox chemistry to a variety of unactivated organoiodide substrates, transformations involving more economical and widely available bromide and chloride substrates remain rare and have only been developed very recently. There are several photoredox transformations on unactivated organobromide and organochloride substrates that require UV excitation.^13–17^ In addition, recent work from Jui's group demonstrated intramolecular hydroarylation reactions involving aryl bromide substrates, using visible light, an organic photosensitizer, and Hünig's base as the only reductant.^18^ However, the vast majority of visible-light photoredox transformations on unactivated organochloride or organobromide substrates require either additional energy input in the form of an applied potential or a second photon, or the use of additional costly additives beyond the sacrificial reductant. A selection of these recent advances in visible-light photoredox activation of challenging organohalide substrates is summarized in Fig. 1. MacMillan's and Stephenson's groups both revealed strategies for photoredox activation of aryl and alkyl bromide substrates which involve a superstoichiometric amount of tris(trimethylsilyl)silane (TTMSS) as an additive.^19,20^ Another recent approach for photoredox activation of challenging substrates is to use two-photon activation. König's work with perylene diimide (PDI)^21^ and Nicewicz's work with acridinium photosensitizers^22^ have shown that certain organic photosensitizers can be reductively quenched to a radical state, which upon second excitation produces a strong reductant capable of reacting with a wide variety of aryl halide substrates. In another approach which requires two-photon excitation, high-power laser irradiation of a water-soluble *fac*-Ir(ppy)_3_ analogue produces hydrated electrons which can reduce aryl or benzyl halides in aqueous solution.^23,24^ And finally, in two works published concurrently, Lin's and Wickens's groups developed strategies that combine electrochemical and photochemical activation of organic photosensitizers, generating reductants strong enough to react with aryl bromide and aryl chloride compounds.^25,26^

All of these aforementioned visible-light approaches, while effective, require some combination of technically challenging reaction conditions, atom-inefficient silane additives, or high-powered laser light sources. An approach that has been much less explored until recently is to tailor new, more reactive photosensitizers capable of reacting directly with more challenging substrates. There have been some promising efforts with low-valent group 6 (Cr, Mo, W) isocyanide complexes with very appealing photophysical and redox attributes,^27,28^ and some early success in photoredox transformations of difficult substrates.^28,29^ The highly reducing organic photosensitizer 10-phenylphenothiazine (PTH) has also been shown to be capable of promoting hydrodehalogenation of a wide range of aryl halide substrates, albeit with UV activation.^30^ Our group has introduced a new class of heteroleptic bis-cyclometalated iridium photosensitizers with the general formula Ir(ppy)_2_(NacNac), where NacNac is a variably substituted β-diketiminate ligand, which have excited-state reduction potentials more potent than *fac*-Ir(ppy)_3_, by ∼300–500 mV.^31^ With some of these photosensitizers we have shown very efficient hydrodebromination of a few aryl bromide substrates, using only blue LED irradiation and an amine base in concert with the photosensitizer.^32^ Relatedly, Connell *et al.* have shown that the well-known photosensitizer [Ir(ppy)_2_(dtbbpy)]^+^ is converted under photoredox reaction conditions to a more reducing charge–neutral complex, *via* semireduction of dtbbpy, and that this modified complex is a much more potent photoreductant and promotes photoredox transformations of a variety of aryl halide and some alkyl bromide substrates.^33^

In this work, we show that the strongly photoreducing Ir(ppy)_2_(NacNac) complexes previously developed by our group (Fig. 1) are versatile and efficient photoredox catalysts. Using simple reaction conditions with only a single additive needed, we demonstrate high-yielding hydrodehalogenation transformations of aryl bromide, aryl chloride, aryl fluoride, and alkyl bromide substrates. In addition to hydrodehalogenation, we also show that these newly developed iridium photocatalysts are effective for other synthetically useful functional group transformations, including ether C–O cleavage, radical addition and cyclization, arylation, prenylation, and ketone reduction. We show with one of the catalysts that green light irradiation results in good product yields for most of these transformations, which may be useful for more advanced applications involving wavelength-control of dual-catalyst systems.^34^ This work shows that strongly reducing photosensitizers can enable reactions on a wider range of substrates to include those not easily amenable to photoredox catalysis, using simple, generalizable reaction conditions for many different transformations.

## Results and discussion

### Choice of photosensitizer and sacrificial reagent

**Fig. 2:**
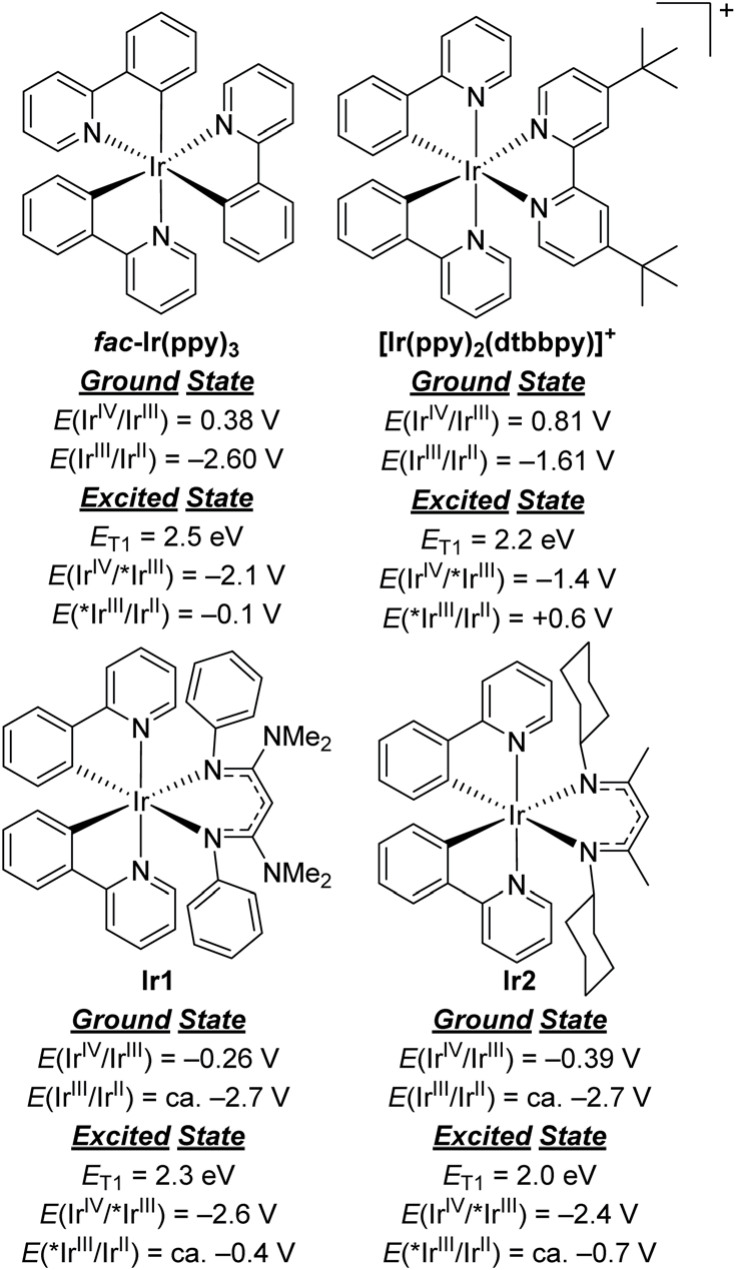
Comparison of ground- and excited-state redox potentials for widely used Ir photosensitizers with **Ir1** and **Ir2**.

To comment briefly on our choice of photosensitizers, we chose complexes **Ir1** and **Ir2** (Fig. 1) for this study, noting that they performed best of the 10 photosensitizers we previously evaluated for aryl bromide hydrodebromination.^32^ These catalysts are synthesized by a general and simple route involving the readily accessible precursor [Ir(ppy)_2_(μ-Cl)]_2_ and the potassium salt of the respective β-diketiminate. Whereas stock solutions of **Ir1** and **Ir2** slowly degrade under ambient conditions, and reactions described here were typically set up in a glovebox, if necessary solids of **Ir1** and **Ir2** can be handled on the benchtop and reactions can be set up outside of a glovebox, as we have previously shown.^32^**Ir1** and **Ir2** are soluble and stable in most aromatic and polar organic solvents and have significant visible absorption beyond 400 nm (Fig. S1 of the ESI†), making them suitable for visible-light photoredox catalysis. The catalysts and their degradation products can easily be separated from organic reaction products by filtering through a short plug of silica. As shown in Fig. 2, the ground- and excited-state redox potentials of **Ir1** and **Ir2** are markedly different than the iridium photosensitizers *fac*-Ir(ppy)_3_ and [Ir(ppy)_2_(dtbbpy)]^+^, both commonly used in photoredox activation of aryl halide substrates.^32,35^ The biggest effect of the β-diketiminate ancillary ligand is to destabilize the HOMO, making **Ir1** and **Ir2** both much easier to oxidize in the ground-state. Their formal Ir^IV^/Ir^III^ redox couples occur below the ferrocene couple, which in combination with their triplet excited-state energies (*E*_T1_) leads to the prediction that **Ir1** and **Ir2** are much stronger excited-state reductants than *fac*-Ir(ppy)_3_ and [Ir(ppy)_2_(dtbbpy)]^+^, with *E*(Ir^IV^/*Ir^III^) = −2.6 V and −2.4 V for **Ir1** and **Ir2**, respectively. We have previously shown that these more potent excited-state reduction potentials correlate with faster photoinduced electron-transfer to model substrates.^31,32^ On the other hand, **Ir1** and **Ir2** are expected to be much weaker excited-state oxidants than the two reference compounds. In short, we expect **Ir1** and **Ir2** to be thermodynamically and kinetically superior for photoredox transformations that involve excited-state reduction steps, which are the types of reactions we studied in this work.

Our previous conditions for hydrodebromination used tetramethylethylenediamine (TMEDA) as the sacrificial reagent. In hopes of further optimizing the reaction conditions and being able to access even more challenging substrates, we evaluated our choice of sacrificial reagent. The role of the sacrificial reductant depends on the reaction mechanism, and either involves reductive quenching of the photosensitizer excited state, or reduction of the oxidized photosensitizer to regenerate the photosensitizer ground state if oxidative quenching is operative. Whichever mechanism is occurring, the reaction could benefit from a more strongly reducing sacrificial reagent. Complexes **Ir1** and **Ir2** are difficult to reduce both in the excited state *E*(*Ir^III^/Ir^II^ < 0 V) and in the oxidized ground state, with *E*(Ir^IV^/Ir^III^) respectively −0.26 and −0.39 V (all potentials are referenced to the ferrocenium/ferrocene couple). The oxidation potentials of tertiary amine bases fall in the range of 0.17–0.52 V, and Pellegrin and Odobel have suggested a list of alternative sacrificial reagents for photocatalysis, many of which are more reducing.^36^ After some preliminary screening of strongly reducing thiolate or dithiocarbamate salts which did not lead to quenching or to particularly robust catalysis, we settled on 1,3-dimethyl-2,3-dihydro-2-phenyl-benzimidazole (BIH, *E*^ox^ = −0.07 V). BIH is known to be a good electron and hydrogen atom donor, and has been adopted in multiple studies including both photoredox catalysis^37,38^ and photocatalytic CO_2_ reduction.^39,40^

### Hydrodebromination of aryl bromides

**Scheme 1:**
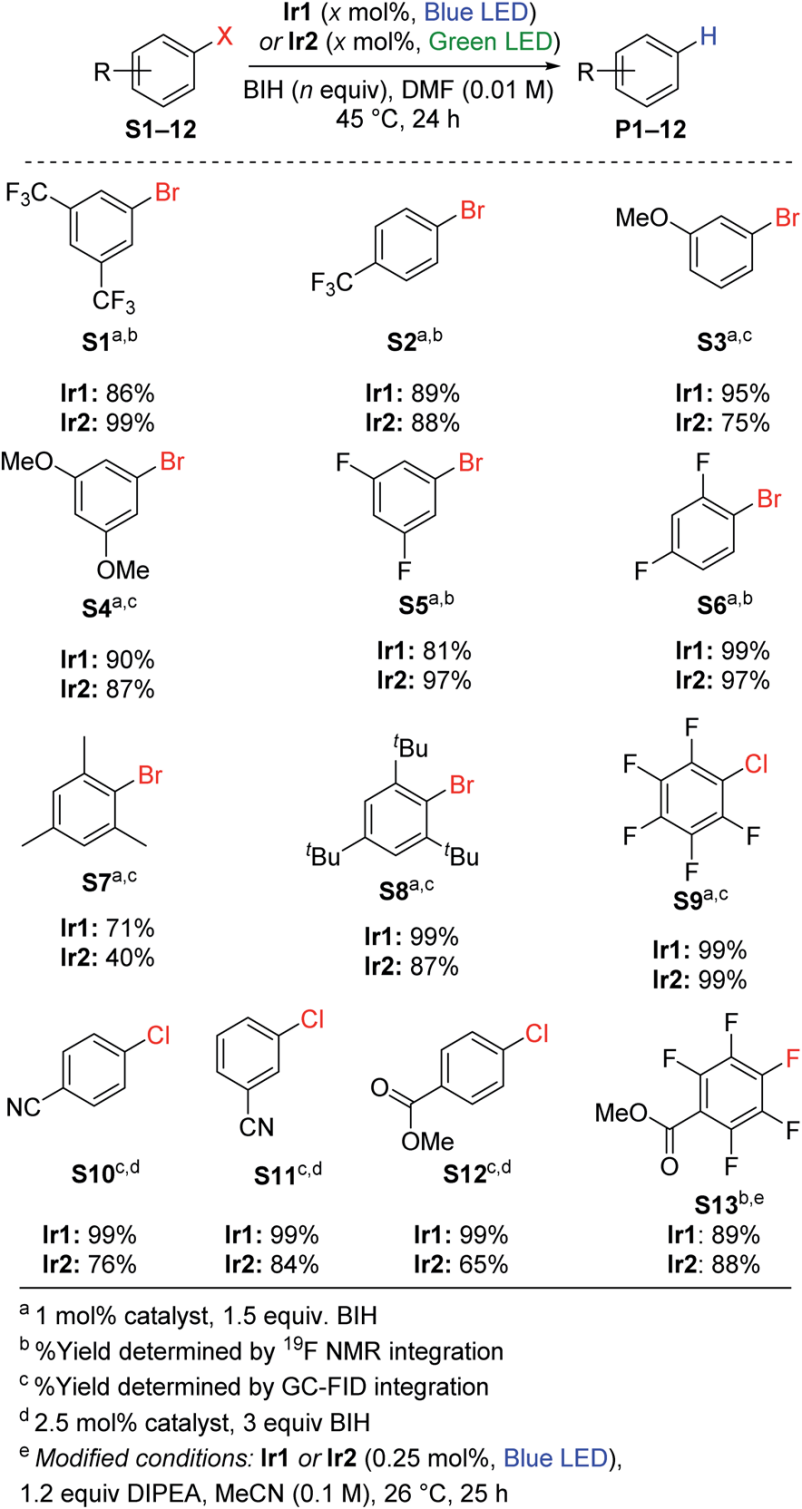
Hydrodehalogenation of aryl halides.

Replacing TMEDA with BIH as the sacrificial reagent allows further optimization of the conditions for aryl bromide hydrodebromination and an expansion of the substrate scope, summarized in Scheme 1. In our previous work where TMEDA was used as the sacrificial reagent, the reactions required 2.5 mol% Ir catalyst and 48 h reaction times for optimal yields at 45 °C, but with BIH we can decrease catalyst loading to 1 mol%, decrease reaction time to 24 h, and still achieve similar or even better product yields. Eight substrates were tested, and in all cases hydrodebromination occurs with moderate to excellent yields. The reaction tolerates electron-poor, electron-rich, and sterically encumbered aryl bromide substrates. Comparing catalyst **Ir1** with blue LED irradiation (465–470 nm) and catalyst **Ir2** with green LED irradiation (520–525 nm; see Fig. S2† for pictures of reaction apparatuses), we observe comparable product yields across the range of substrates tested. Control experiments with the substrates 4-trifluoromethylbromobenzene (**S2**) and 3-methoxybromobenzene (**S3**), using identical conditions but *without* the Ir catalyst added, did not lead to the formation of the respective arene products **P2** and **P3**, indicating that BIH is not a catalyst on its own. One possible complication with BIH or even amine-based sacrificial reagent in photoredox catalysis is that the radical cation state (BIH˙^+^ or NR_3_˙^+^) can participate in a dark radical chain reaction^41^ with the substrate, following an initiation step involving the photosensitizer and light. To check the possibility of radical chain reactions, we have measured the photochemical quantum yield (*Φ*) for the formation of product from hydrodebromination of **S2** and **S7**. The measured quantum yields are 4.7% for the conversion of **S2** to **P2**, and 1.4% for the conversion of **S7** to **P7**. Whereas a quantum yield >100% must indicate dark radical chain processes and the quantum yields we obtained do not explicitly rule out chain reactions following a slow initiation step, the results are in line with other recently reported quantum yields for photocatalytic transformations and suggest that radical chain reactions are not a dominant pathway.^37,42,43^ One other experiment in support of this conclusion is summarized in Fig. S3 of the ESI,† where we monitored the hydrodebromination of 2-bromomesitylene with alternating light/dark cycles. As shown in Fig. S3,† hydrodebromination progress ceases in the absence of light, indicating that if there are radical chain processes, they are short-lived and not persistent over long timescales.

### Hydrodechlorination of aryl chlorides

Using the same conditions optimized for hydrodebromination of aryl bromides, we moved on to more challenging aryl chloride substrates. We did achieve successful hydrodechlorination reactions of aryl chlorides, also summarized in Scheme 1, which is rare in visible-light photoredox catalysis.^23,25,26,33^ These reactions required higher catalyst loading (2.5 mol%) and a larger excess of BIH (3 equiv.) for optimization, but otherwise conditions and reaction times are the same as those in Scheme 1 for hydrodebromination. One limitation is that the substrate for hydrodechlorination must have one or more electron-withdrawing groups to be activated, suggesting that the electron-transfer step is limiting with these substrates. Nevertheless, with optimized conditions we were able to achieve high-yielding hydrodechlorination of four aryl chloride substrates, with quantitative yields when using catalyst **Ir1** and blue LED irradiation, and slightly lower yields with catalyst **Ir2** and green LED irradiation.

### Hydrodefluorination

Recently photoredox catalysis has been shown to be a promising strategy to break even strong and recalcitrant aryl C–F bonds and is limited by the necessary deep reduction. There has been a surge of recent interest in photoredox catalysis on aryl fluoride^44–48^ or trifluoromethylated substrates,^45,49^ which provides convenient access to pharmaceutically relevant partially fluorinated aromatics. Whereas all recent developments in extreme photoredox reductions have focused on organobromide and organochloride substrates,^20,21,23,25,26,29,33^ we are also interested in the efficacy of catalysts **Ir1** and **Ir2** for hydrodefluorination. Using similar conditions to those optimized by Weaver's group, we demonstrated high-yielding *para*-selective hydrodefluorination (HDF) of the substrate **S13**, forming product **P13** in good yield using either **Ir1** or **Ir2** as the catalyst with blue LED illumination (Scheme 1). The regioselectivity of the HDF remains the same, and with only half the catalyst loading the yield is slightly higher than that obtained with *fac*-Ir(ppy)_3_ as the catalyst.^44^ As part of our optimization of the HDF reaction we screened several other catalyst variants (**Ir3–Ir8**, see Table S1†), with the HDF results summarized in Table S2.† A few other catalysts, **Ir3–Ir6**, performed as well as **Ir1** and **Ir2**, but none were clearly superior for HDF.

### Hydrodebromination of alkyl bromides

**Scheme 2:**
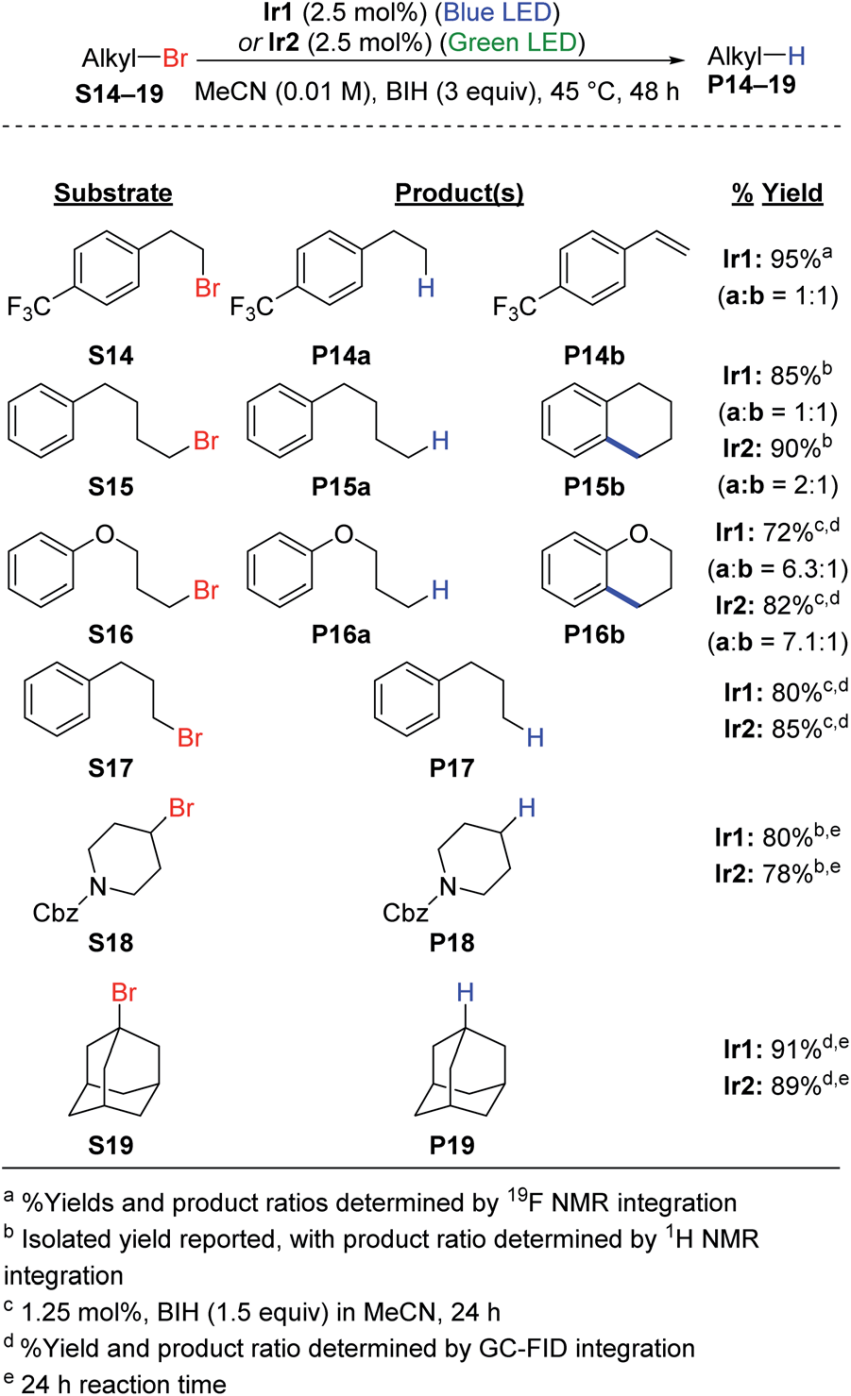
Hydrodebromination and intramolecular radical addition of unactivated alkyl bromides.

Hydrodebromination of alkyl bromides remains rare in photoredox catalysis, with the most systematic study from Stephenson's group using reaction conditions that include excess TTMSS as an additive, in addition to excess DIPEA,^20^ and Connell *et al.* recently showed that TTMSS additive is not required for two alkyl bromide substrates.^33^ As shown in Scheme 2, the newly optimized hydrodehalogenation conditions presented here are also operable for unactivated alkyl bromide substrates. The reactions require 2.5 mol% of Ir catalyst with 3 equiv. of BIH, avoiding wasteful silane additives that were previously used with alkyl bromide substrates.^19,20^

The reactions tolerate primary (**S14–S17**), secondary (**S18**), and tertiary alkyl bromide (**S19**) substrates. The primary alkyl bromide substrates tested all have tethered aryl rings, and three involved side reactions and thus did not exclusively produce the straight-chain alkane product. For substrate **S14** HBr elimination competed with hydrodebromination, resulting in a 1 : 1 mixture of the alkane (**P14a**) and substituted styrene (**P14b**) products. As shown in Fig. S4,† replacing BIH with DIPEA or TMEDA improves the selectivity for **P14a**, albeit at the expense of lower conversion. For substrates **S15** (alkane) and **S16** (ether), intramolecular radical addition can occur following C–Br cleavage, resulting in cyclized side products. Similar intramolecular radical addition reactions to an aryl group have been studied by Stephenson *et al.*^50^ and Zhang *et al.*^51^ For **S15** nearly equal amounts of the linear (**P15a**) and cyclized **(P15b**) products are formed (Fig. S5†), with the ratio depending slightly on the catalyst choice and excitation wavelength. Efforts to optimize the ratio between **P15a** and **P15b** did not result in further improvement (Fig. S6 and S7†). Reactions are more sluggish in MeCN compared to DMF (Fig. S7†) and replacing BIH with TMEDA does not improve conversion or yield (Fig. S6†). For **S16**, the linear hydrodebromination product **P16a** was preferred, forming in a 6 : 1 to 7 : 1 ratio with the cyclic ether product **P16b** (Fig. S8†). For the rest of the reactions, hydrodebromination is the only outcome observed. Substrate **S17** does not undergo radical addition to form a fused five-membered ring, and only the linear product **P17** is observed in good yield (Fig. S8†). For the secondary (**S18**) and tertiary (**S19**) alkyl bromide substrates, we observe hydrodebromination exclusively. Unlike **Ir1** and **Ir2**, *fac*-Ir(ppy)_3_ does not promote effective hydrodebromination of 1-bromoadamantane (**S19**), with majority starting material remaining after 24 h (Fig. S9†).

### Radical cyclization

**Scheme 3:**
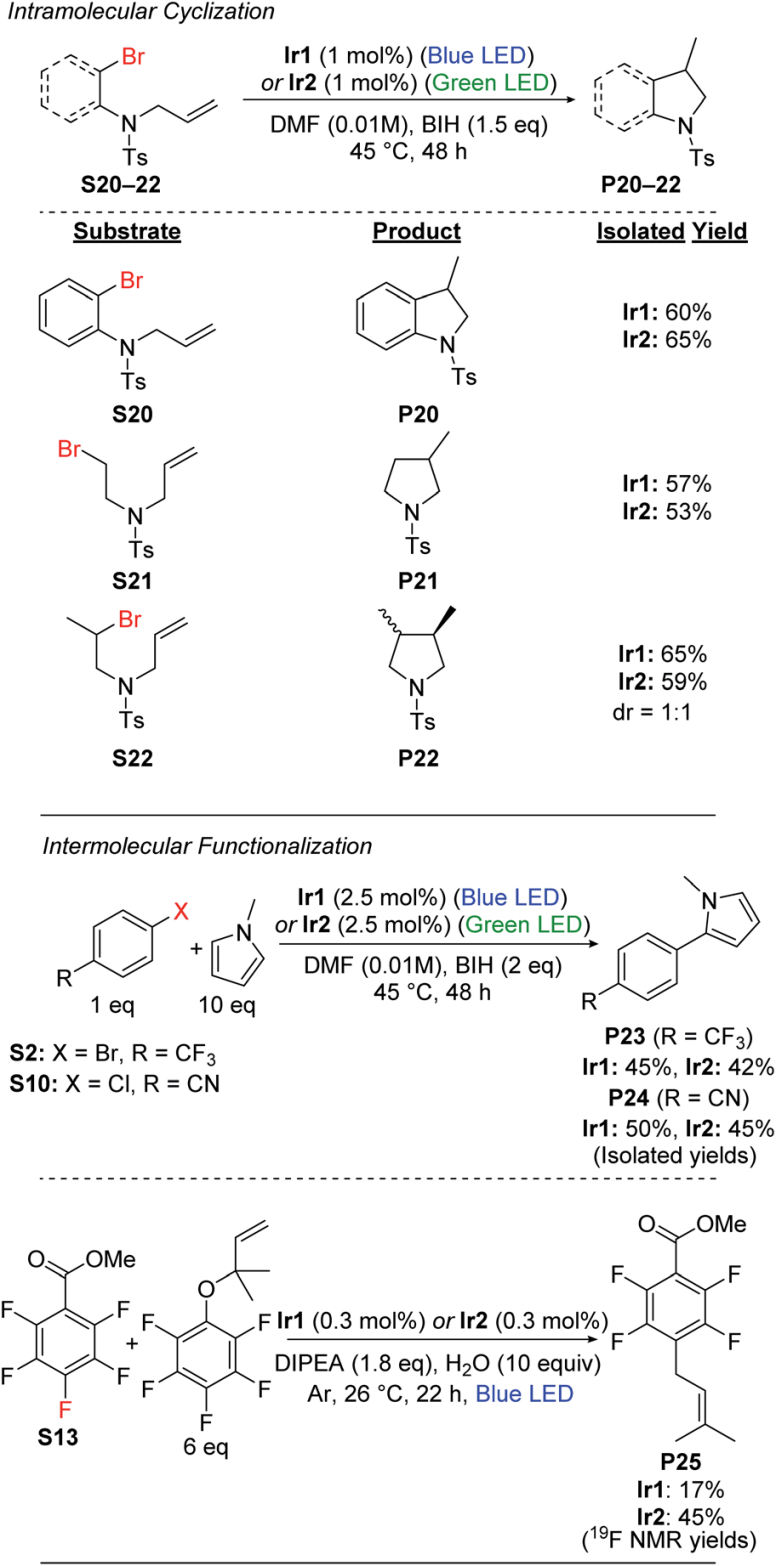
Intramolecular and intermolecular functionalization reactions.

Having demonstrated efficient hydrodebromination of alkyl and aryl bromides (Schemes 1 and 2), and observing cyclization side products in some of the cases (Scheme 2), we next turned our attention to substrates with tethered allyl substituents, which are poised for radical cyclization following C–Br bond cleavage. Scheme 3 summarizes the results with three such substrates, where the cyclization is initiated either by cleavage of an aryl C–Br bond (**S20**) or an alkyl C–Br bond (**S21** and **S22**). Cyclization of these substrates occurs under the same conditions as previously optimized for hydrodebromination, and forms substituted heterocyclic indoline (**P20**) or pyrrolidine (**P21** and **P22**) products. In all cases, moderate yields of the cyclized products are obtained, with no major differences between catalyst **Ir1**(blue LED) or **Ir2** (green LED). For the secondary alkyl bromide substrate **S22**, the two diastereomeric products are formed in a 1 : 1 ratio, further supporting the radical nature of the reaction.

### Intermolecular functionalizations

We also investigated intermolecular functionalization reactions catalyzed by **Ir1** and **Ir2**, where the organic radical that is generated is intercepted by a second reagent. We initially chose the radical acceptor *N*-methylpyrrole for these studies, previously used by König and coworkers.^42^ We subjected aryl bromide substrate **S2** and aryl chloride substrate **S10** to our optimized conditions in the presence of 10 equiv. of *N*-methylpyrrole, which resulted in moderate yields of the arylated products (**P23** and **P24**) along with 20–25% yield of the respective hydrodehalogenation product (Scheme 3). We attempted the same transformation using the alkyl bromide substrates **S18** and **S19**, but with *N*-methylpyrrole present, we still only observed hydrodebromination products.

We also investigated prenylation reactions^52^ of the aryl fluoride substrate **S13** (Scheme 3), intercepting the radical with 6 equiv. of a secondary prenylation reagent. These reactions were screened with eight different catalysts from our library (Table S3†), and in all cases we observed modest conversion and modest yield of the desired product **P25**. The catalyst **Ir2** was among the best for this transformation, with **P25** formed in 45% yield, but even in this case we observed 14% of the HDF product **P13** and 41% of unconverted starting material after 22 h, with no further conversion after 68 h. Also, as shown in Table S3 in the ESI,† we observe quantitative mass balance in these reactions, indicating there are not deleterious side reactions involving **S13**. Taken together, the results of these studies seem to indicate catalyst decomposition under the reaction conditions, as in all cases modest conversion is observed after 22 h, but no improvement in yield is observed after 68 h. We suspect that the rather acidic pentafluorophenol byproduct, formed following prenyl group transfer, reacts deleteriously with the electron-rich, highly basic β-diketiminate ligands in **Ir1** and **Ir2**.

### Activation of C–O bonds

**Scheme 4:**
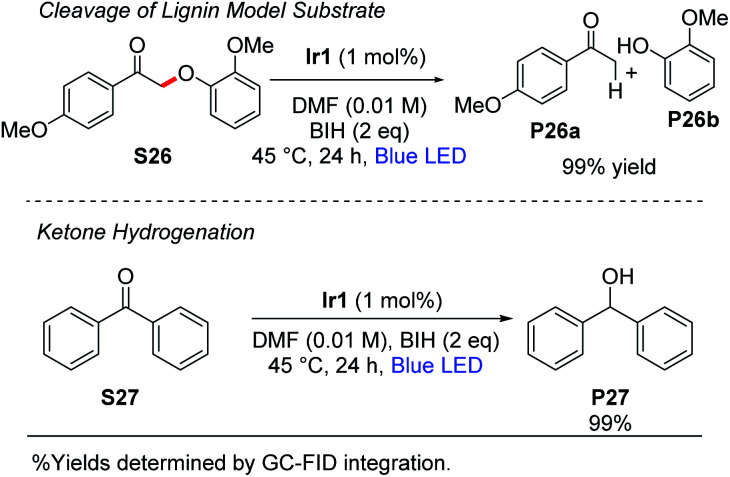
Activation of substrates with C–O bonds.

Stephenson and coworkers have introduced a two-step strategy for cleavage of β-O-4 linkages in substrates relevant to lignin depolymerization, which proceeds *via* oxidation of the substrate secondary alcohol to a ketone, followed by C_α_–O cleavage under photoredox conditions using [Ir(ppy)_2_(dtbbpy)]^+^ as the catalyst.^53,54^ Using the typical reaction conditions described here, with 1 mol% of **Ir1** and 2 equiv of BIH, we observed near-quantitative cleavage of **S26** to the respective ketone (**P26a**) and phenol (**P26b**) products (Scheme 4). We did not observe any cleavage of native lignin model substrates that had not been pre-oxidized to the ketone. Finally, as also summarized in Scheme 4, the combination of **Ir1** and BIH was effective for the hydrogenation of benzophenone (**S27**) to diphenylmethanol (**P27**). Benzophenone is difficult to reduce and has a reduction potential of −2.2 V *vs.* Fc^+^/Fc.^55,56^ Thus, photoredox activation is uncommon although it has been previously reported,^57^ and chemical reduction typically requires hydridic reagents like sodium borohydride. Quantitative reduction of **S27** to **P27** was observed with 1 mol% **Ir1**, 2 equiv of BIH, and blue LED irradiation for 24 h. This outcome deviates from previously reported photoredox reactions of benzophenone and other aromatic ketones, which use amine sacrificial reagents and proceed *via* reductive C–C coupling to form substituted pinacol products.^16,57^ In our reaction conditions, where BIH is used as the sacrificial reductant in combination with **Ir1** or **Ir2**, the ketyl radical intermediate that is formed *via* SET to the ketone is trapped before it can dimerize.

This either suggests that the ketyl radical does not build up to a substantial concentration in our reaction mixture to allow bimolecular combination, or that BIH is more effective at trapping ketyl radicals *via* hydrogen atom transfer than an amine is. We hypothesize that two equivalents of BIH are involved in this transformation to generate the H_2_ equivalent that adds to benzophenone.

### Comparison with *fac*-Ir(ppy)_3_

Also of interest in this study is how the catalytic performance of complexes **Ir1** and **Ir2** compares with *fac*-Ir(ppy)_3_, historically the most widely used photosensitizer for challenging reductive transformations. We also carried out hydrodehalogenation on select substrates using *fac*-Ir(ppy)_3_ as the photocatalyst under blue light irradiation, using otherwise identical conditions to those outlined in Schemes 1 and 2. The product yields are summarized in Table 1 and compared directly with the yields obtained with **Ir1** (blue light irradiation) and **Ir2** (green light irradiation). For one aryl bromide substrate, **S1**, and one aryl chloride substrate, **S9**, we obtained near-quantitative yields with *fac*-Ir(ppy)_3_, as we did with **Ir1** and **Ir2**. However, for the remainder of the tested substrates we obtained lower yields with *fac*-Ir(ppy)_3_. This includes the previously reported hydrodefluorination of **S13**, which proceeded to 75% yield with *fac*-Ir(ppy)_3_ with 0.5 mol% loading,^44^ compared to 89% and 88% with **Ir1** and **Ir2**, at half the catalyst loading. In addition, for aryl bromide substrates **S2**, **S5**, and **S6**, hydrodebromination yields ranged from 66% to 74% with *fac*-Ir(ppy)_3_, compared with 81% to 99% with **Ir1** and **Ir2**. Finally, for adamantyl bromide (**S19**) the yield of hydrodebromination is only 25% with *fac*-Ir(ppy)_3_, compared to 91% and 89% with **Ir1** and **Ir2**, respectively. Thus, for most of the tested hydrodebromination reactions **Ir1** and **Ir2** outperform *fac*-Ir(ppy)_3_.

**Table 1:** Comparison of hydrodehalogenation yields for select substrates, using *fac*-Ir(ppy)_3_, **Ir1**, and **Ir2** as photosensitizers

Substrate	*fac*-Ir(ppy)_3_^a^	Ir1	Ir2
**S1**	99%	86%	99%
**S2**	66%	89%	88%
**S5**	69%	81%	97%
**S6**	74%	99%	97%
**S9**	99%	99%	99%
**S13**	75% (ref. 44)^b^	89%	88%
**S19**	25%	91%	89%

a Conditions identical to those shown in Schemes 1 and 2 unless otherwise noted. For *fac*-Ir(ppy)3 blue light irradiation was used.

b 0.5 mol% *fac*-Ir(ppy)_3_.

### Mechanistic considerations

**Scheme 5:**
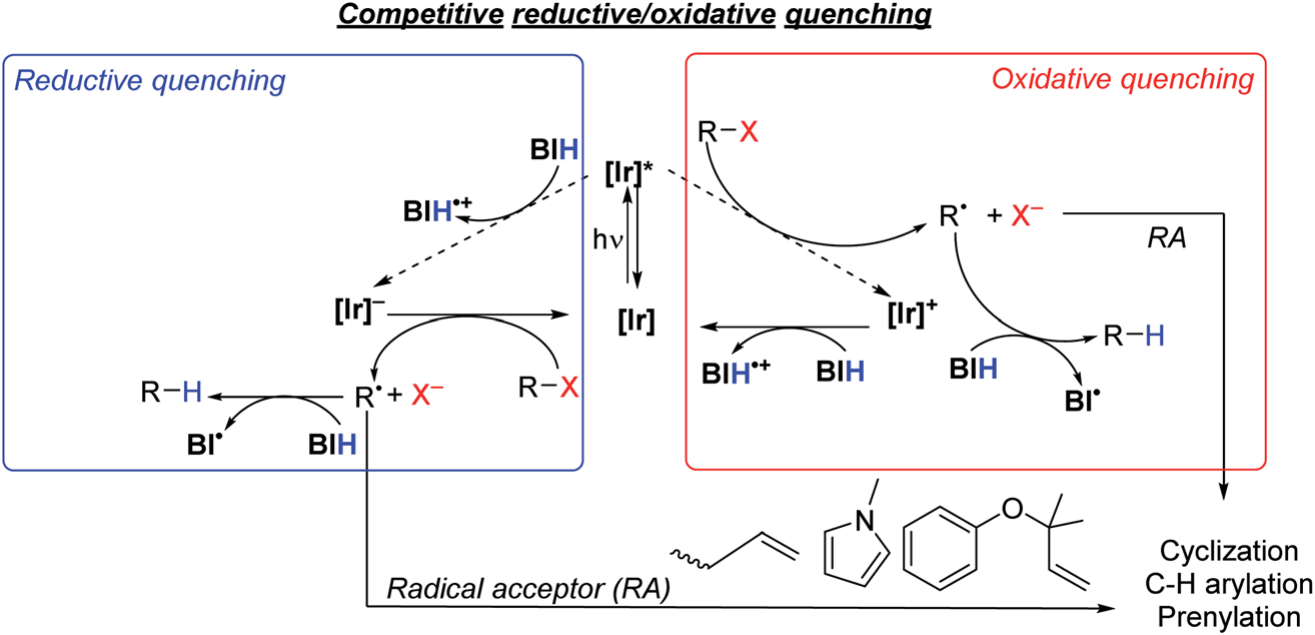
Proposed quenching pathways involving iridium photosensitizers, [Ir], and substrates to generate organic radical intermediates.

As summarized in Scheme 5, there are two limiting mechanisms for photoredox activation of C–X bonds. Experiments described above and summarized in the ESI (Section VI and Fig. S3†) speak against a radical chain mechanism, leaving more typical oxidative and reductive quenching pathways as the likely mechanisms. In the oxidative quenching mechanism, the excited Ir photosensitizer ([Ir]*) donates an electron to the substrate directly, which fragments to form an aryl or alkyl radical. In the presence of BIH donation of a hydrogen atom furnishes the hydrodehalogenation product, but if a suitable intra- or intermolecular radical trap is present, functionalization can occur. For the reductive quenching mechanism, the net outcome is the same, but the photosensitizer in its excited state instead accepts an electron from BIH, and the reduced photosensitizer (**[Ir]−**) reacts with the substrate to generate the substrate radical. The formation of R˙ and X^−^ following electron transfer to R–X is typically a stepwise process, where electron transfer generates [R–X]˙^−^, which then fragments.^58,59^ Unproductive geminate recombination is thus possible if the reduced [R–X]˙^−^ species has a sufficient lifetime, and this may factor into the low product-formation quantum yields we observe with **S2** and **S7** (see above). Acknowledging the importance of the stepwise fragmentation of R–X, we still show it in Scheme 5 as a single step for simplicity, and once formed the fate of R˙ depends on what other reagents are available to trap it.

Thermodynamically, the oxidative quenching pathway should be preferred. The excited-state Ir^IV^/*Ir^III^ potentials of **Ir1** and **Ir2** are −2.6 and −2.4 V *vs.* Fc^+^/Fc (see Fig. 2), which is enough reducing power to transfer an electron to most aryl halide substrates.^59,60^ In contrast, the excited-state *Ir^III^/Ir^II^ potentials of **Ir1** and **Ir2** are estimated to be −0.4 V and −0.7 V, more negative than the *E*^ox^ of BIH (−0.07 V),^61^ indicating that reductive quenching by BIH should not be thermodynamically favorable. We do note that the *Ir^III^/Ir^II^ potentials are a crude estimate since we could not identify a clear Ir^III^/Ir^II^ wave in **Ir1** and **Ir2** and the triplet-state energy has an uncertainty of ±0.1 eV. Nevertheless, it seems that of the two initial photoreactions outlined in Scheme 5, oxidative quenching by the substrate is thermodynamically more likely.

In an attempt to determine the kinetically preferred pathway for R˙ formation, we performed Stern–Volmer quenching experiments of **Ir1** with two substrates and BIH. Our prior investigations, conducted in MeCN with several aryl bromide substrates, including **S1**, **S3**, **S6**, and **S7**, revealed that oxidative quenching by the substrate (*k*_q_ = 0.078–4.6 × 10^8^ M^−1^ s^−1^ in MeCN)^32^ was generally preferred over reductive quenching by the sacrificial reagent TMEDA (*k*_q_ = 6.3 × 10^6^ M^−1^ s^−1^ in MeCN). In this work our typical optimized reactions were carried out in a different solvent (DMF) with a different sacrificial reagent (BIH), prompting us to reevaluate the kinetics of some of the key electron-transfer steps. Despite reductive quenching seemingly being thermodynamically disfavored, BIH quenches the excited state of **Ir1** (Fig. S10†) with an observed rate constant of 2.3 × 10^8^ M^−1^ s^−1^ in DMF. We note a similar, albeit slightly smaller quenching rate constant of 1.5 × 10^8^ M^−1^ cm^−1^ for *fac*-Ir(ppy)_3_ with BIH, which on the basis of redox potentials (Fig. 1) should be faster if reductive quenching were occurring. Noting also that Stern–Volmer alone does not identify the quenching mechanism,^62^ we cannot conclusively state that the quenching by BIH is in fact electron-transfer quenching, and it may be caused by unproductive triplet–triplet energy-transfer without electron transfer. Regardless of the precise quenching pathway with BIH, its *k*_q_ value exceeds the quenching rate constant for aryl bromide substrate **S7** (1.9 × 10^8^ M^−1^ s^−1^) and aryl chloride substrate **S10** (8.1 × 10^7^ M^−1^ s^−1^), which almost certainly involve oxidative electron-transfer quenching. This observation, in concert with the practical consideration that all reactions in this study were conducted with excess BIH (1.5–3 equiv.), indicates that quenching by BIH is the kinetically preferred pathway. That said, this outcome does not guarantee that the quenching by BIH is productive, and the rate constants for the two quenching pathways appear to be similar. For some of the more easily reduced substrates (*e.g.***S1** and **S9**) direct quenching by the substrate is likely competitive with or even faster than quenching by BIH. Thus, these studies don't conclusively identify a mechanism, but they do suggest that the quenching pathways involving BIH and the substrate are kinetically competitive.

Considering the likely importance of substrate oxidative quenching, we also investigated select quenching rate constants of *fac*-Ir(ppy)_3_ in comparison with **Ir1**. We have previously described oxidative quenching rate constants of model substrates methyl viologen and benzophenone with *fac*-Ir(ppy)_3_, **Ir1**, **Ir2**, and several other NacNac-supported bis-cyclometalated iridium photosensitizers.^31,32^ We showed in this previous work that the greater reducing power of **Ir1** and **Ir2** leads to significantly faster excited-state electron transfer, particularly for benzophenone which has a reduction potential similar to typical aryl halides.^56^ As noted above we measured quenching rate constants of aryl bromide substrate **S7** (1.9 × 10^8^ M^−1^ s^−1^) and aryl chloride substrate **S10** (8.1 × 10^7^ M^−1^ s^−1^) in DMF, using **Ir1** as the photosensitizer. These quenching rate constants exceed those of *fac*-Ir(ppy)_3_ with the same substrates, by a factor of 1.4 (**S7**, *k*_q_ = 1.4 × 10^8^ M^−1^ s^−1^) and 1.5 (**S10**, *k*_q_ = 5.3 × 10^7^ M^−1^ cm^−1^), continuing the trend that oxidative quenching with the more strongly photoreducing **Ir1** is faster than *fac*-Ir(ppy)_3_. Thus, for transformations where oxidative quenching is an important elementary step, the more reducing excited state of **Ir1** can lead to significantly faster rates of excited-state electron transfer, compared to *fac*-Ir(ppy)_3_.

The thermodynamic and kinetic data described above is only relevant to electron-transfer steps that occur from the photosensitizer excited state. Another interesting mechanistic consideration is the precise role of the BIH sacrificial reagent, particularly as it pertains to the hydrogen-atom transfer steps. The electron and hydrogen-atom transfer chemistry of BIH is well understood.^61,63^ BIH itself is a reasonably strong reductant, with *E*(BIH˙^+^/BIH) = −0.07 V. In addition, the C–H bond strength in BIH is considerably weaker than that of a typical alkene or arene. As shown in Scheme 5 it is likely that the organic radical that forms, R˙, can be trapped by BIH to form R–H. However, it is worth noting that the C–H bond strength in BIH˙^+^, which would form following electron-transfer from BIH, is over 40 kcal mol^−1^ weaker than that of BIH,^61^ so it is possible that this radical cation can serve as the hydrogen atom donor provided it is able to diffuse to the organic radical. It is also possible to consider a radical chain mechanism that propagates *via* the BI˙ radical, although this alternative is not favored by the available thermodynamic and mechanistic data. BI˙, which would form following hydrogen atom transfer from BIH, is itself a potent reductant with *E*(BI^+^/BI˙) = −2.06 V.^61^ This reducing potential is below that required to reduce a typical aryl halide or alkyl halide, for which *E*^red^ lies beyond −2.3 V.^59,60^ Moreover, the excited states of **Ir1** and **Ir2** (*E*(Ir^IV^/Ir^III^) = −2.4 to −2.6 V) and their reduced state that would form *via* reductive quenching (*E*(Ir^III^/Ir^II^) ∼ −2.7 V) are both substantially more potent as reductants. In addition, as discussed above the quantum yield data and light “on/off” experiments argue against a dark radical chain mechanism. For these reasons, we favor the oxidative/reductive quenching mechanisms shown in Scheme 5, as opposed to the radical chain process involving electron transfer to the substrate by BI˙ and hydrogen atom transfer to the substrate radical by BIH.

## Conclusions

We have demonstrated that bis-cyclometalated iridium complexes with β-diketiminate ancillary ligands are versatile photosensitizers for a variety of photoredox transformations. The highly reducing nature of their excited state permits an expansion of the substrate scope to challenging organobromide and organochloride substrates, which traditionally are inert to photoredox catalysis or require forcing, complex reaction conditions to activate. These reactions generate an organic radical following photoinduced electron transfer, and the fate of this radical depends on the nature of the substrate and the reaction conditions. In most cases under typical reaction conditions, hydrodehalogenation is the outcome, which establishes the breadth of the substrate scope enabled by these reaction conditions. If the substrate has an appropriately positioned aryl or alkenyl group, radical cyclization is possible, and reactions conducted in the presence of a second radical trap substrate can result in bimolecular functionalization. Overall, this work motivates the continued pursuit of designer photocatalysts and modified photoredox reaction conditions that will allow a much wider range of cheap, readily available substrates to be used in synthetically valuable photoredox transformations.

## Conflicts of interest

There are no conflicts to declare.

## Supplementary Material

SC-012-D0SC06306A-s001
