# Supplementary material for: Photoredox catalysis on unactivated substrates with strongly reducing iridium photosensitizers
Source: Chem Sci. 2021 Jan 29;12(11):4069–78. doi: 10.1039/d0sc06306a (PMC8179447; doi:10.1039/d0sc06306a)
Supplement: SC-012-D0SC06306A-s001 [file SC-012-D0SC06306A-s001.pdf]

Electronic Supplementary Material (ESI) for Chemical Science.
This journal is © The Royal Society of Chemistry 2021

Electronic Supplementary Information

Photoredox Catalysis on Unactivated Substrates with Strongly Reducing Iridium Photosensitizers

Jong-Hwa Shon,† Dooyoung Kim,† Manjula D. Rathnayake,§ Steven Sittel,† Jimmie Weaver,§ and Thomas S. Teets*,†

† University of Houston, Department of Chemistry, 3585 Cullen Blvd., Room 112, Houston, TX 77204-5003, USA

§ Oklahoma State University, Department of Chemistry, Stillwater, OK 74078, USA

email: tteets@uh.edu

Contents

Index	Page
UV-vis absorption spectra of **Ir1** and **Ir2**	S2
Photographs of photoredox reaction apparatuses	S3
Time course of a representative photoredox reaction	S4
Photophysical and electrochemical properties of the photosensitizers	S5
Reaction optimization	S6–S13
Experimental section	S14–S26
Reaction Monitoring with GC and NMR	S27–S40
NMR spectra of isolated products	S41–S52
^{19}F NMR spectra for hydrodehalogenation reactions with *fac*-Ir(ppy)$_3$	S53–S57
ESI References	S58

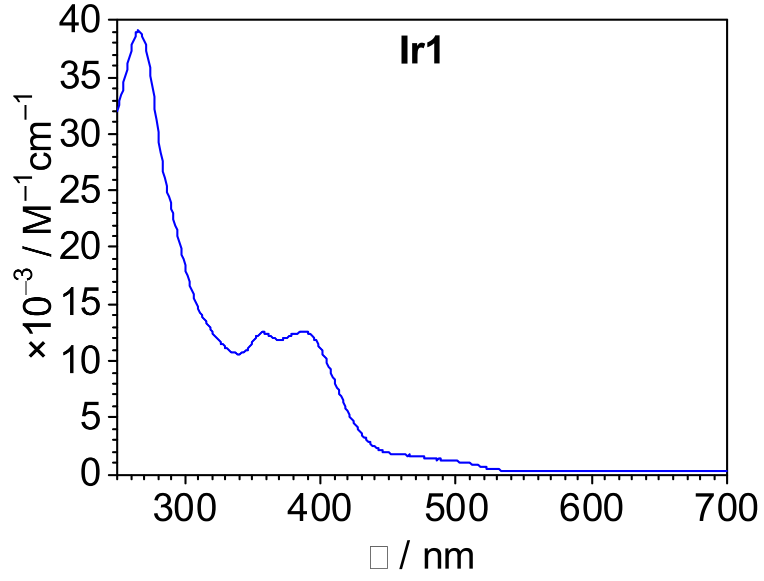

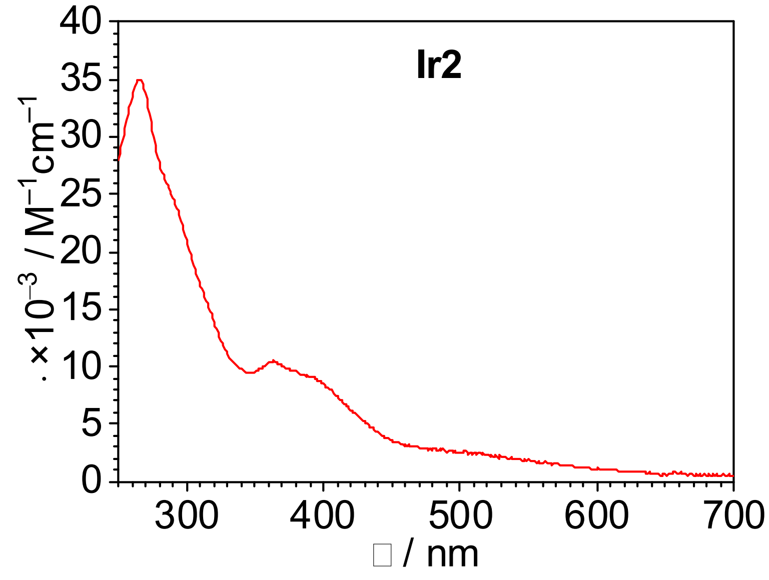

Figure S1. UV-vis absorption spectra of photosensitizers **Ir1** and **Ir2**, recorded at room temperature in MeCN. These spectra were originally reported elsewhere.[1,2]

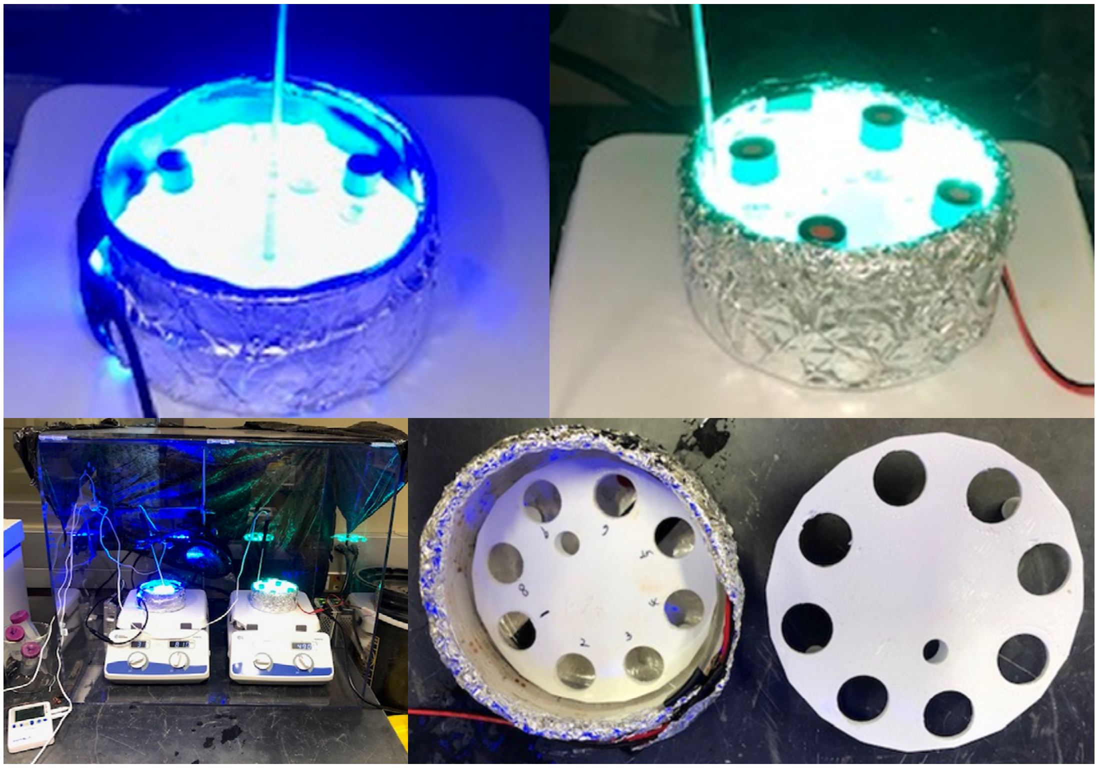

Figure S2. Experimental setup for the photoredox catalysis under blue LED irradiation (top, left) and green LED irradiation (top, right). Reaction apparatus for benchtop reactions (bottom, left). Vial holders prepared by 3D printing (bottom, right).

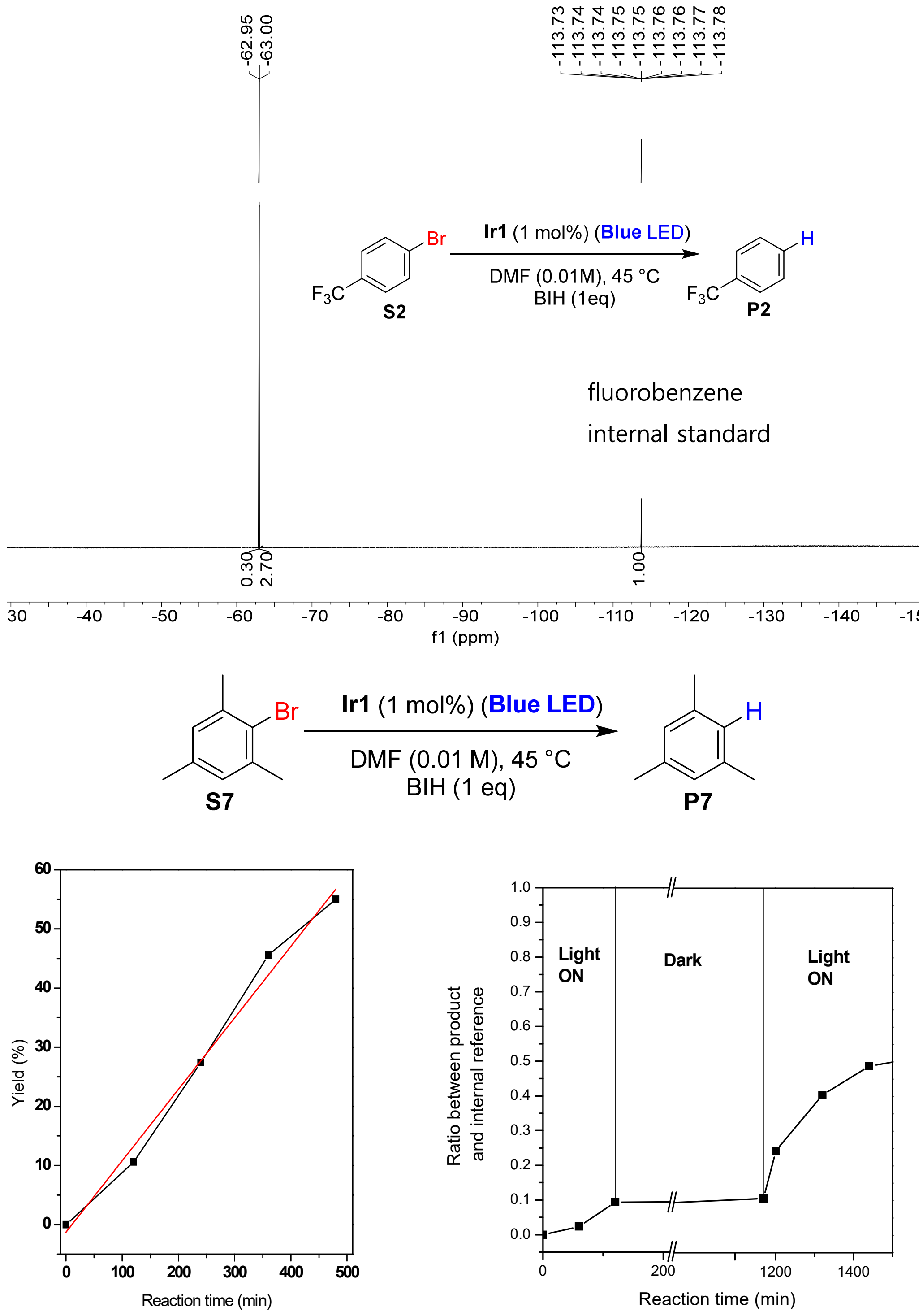

Figure S3. Representative data for monitoring photoredox reaction progress. Top: ^{19}F NMR spectrum following the hydrodebromination of **S2**. Bottom left: Time course for hydrodebromination of **S7**. Bottom right: On/off progress for hydrodebromination of **S7.**

Table S1. Summarized photophysical and electrochemical properties of **Ir1**–**Ir8** and molecular structures of **Ir3**–**Ir8.**[1,2]

	$E(Ir^{IV}/Ir^{III})$ / V vs. Fc^+/Fc	UV-vis Absorption λ / nm ($\varepsilon/10^3$ $M^{-1}cm^{-1}$)[a]	emission, λ / nm		Φ_{PL}[a]	τ / μs[a]	E_{T1} / eV[d]	$E(Ir^{IV}/{}^*Ir^{III})$ / V vs. Fc^+/Fc
			293 K[a]	77 K[c]				
**Ir1**	−0.26	269 (27), 298 (21), 393 (6.1), 511 (1.9)	634	560, 594 (sh)	0.16	0.76	2.3	−2.6
**Ir2**	−0.39	265 (36), 288 (sh) (26), 361 (10) 391 (sh), 506 (2.4)	661	607, 658 (sh)	0.040	0.35	2.0	−2.4
**Ir3**	−0.10	259 (50), 287 (sh) (34), 308 (sh) (23), 365 (12), 391 (15), 500 (1.4)	605	553, 590 (sh)	0.18	0.53	2.3	−2.4
**Ir4**	−0.11	260 (14), 290 (sh) (8.7), 353 (3.0), 385 (sh) (2.9), 435 (0.5)	576	536, 575 (sh)	0.021	0.52	2.4	−2.5
**Ir5**	−0.23	259 (14), 289 (sh) (7.2), 357 (sh) (3.4), 385 (3.8), 435 (0.5), 488 (sh) (0.3)	[b]	547, 589 (sh)	[b]	[b]	2.3[e]	−2.5
**Ir6**	−0.27	269 (15), 304 (sh) (10), 391 (2.3), 494 (0.6)	594	558, 593 (sh)	0.12	0.51	2.3	−2.6
**Ir7**	−0.10	245 (22), 307 (7.5), 308 (6.7)	[b]	533	[b]	[b]	2.3[e]	−2.4
**Ir8**	−0.25	240 (39), 302 (22), 400 (5.9)	637	570	0.017	0.35	2.2[e]	−2.4

[a] In MeCN unless otherwise noted. [b] Not luminescent at 293 K. [c] In butyronitrile. [d] Determined from the intersection point of UV-vis absorption and emission unless otherwise noted. [e] Determined from the first vibronic peak in the 77-K emission spectrum.

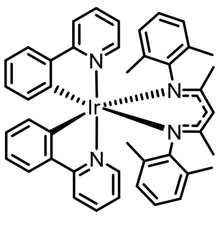

Ir3

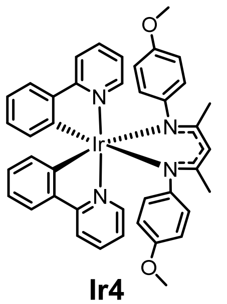

Ir4

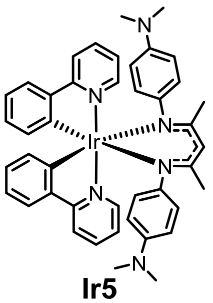

Ir5

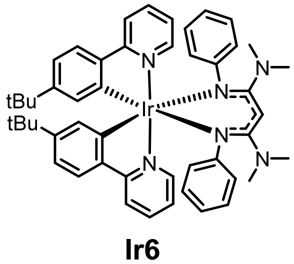

Ir6

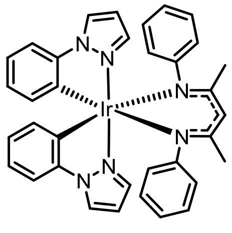

Ir7

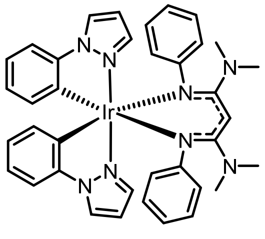

Ir8

Table S2. Catalyst screening for the hydrodefluorination reaction.

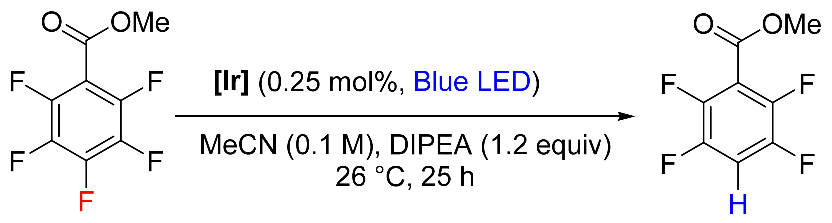

S13 **P13**

[Ir] catalyst	Time (h)	S13%	P13%	Other pdt%
**Ir1**	6	18%	73%	9%
	25	0	89%	11%
**Ir2**	6	28%	65%	7%
	25	0	88%	12%
**Ir3**	6	53%	42%	5%
	25	0	88%	12%
**Ir4**	6	64%	34%	2%
	25	16%	74%	10%
	72	0	88%	12%
**Ir5**	6	26%	65%	9%
	25	0	88%	12%
**Ir6**	6	22%	69%	9%
	25	0	88%	12%
**Ir7**	6	88%	12%	0
	25	85%	15%	0
	72	75%	25%	0
**Ir8**	6	92%	8%	0
	25	87%	13%	0
	72	71%	26%	3%

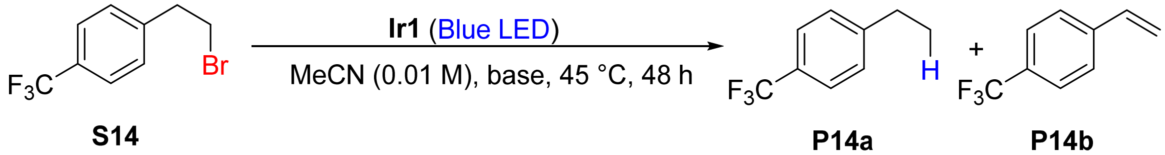

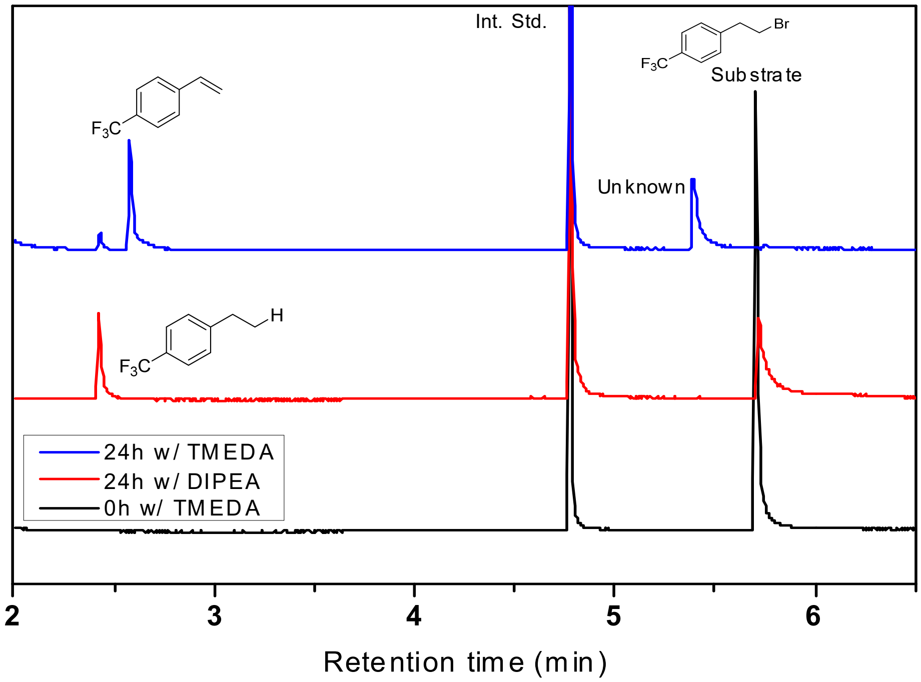

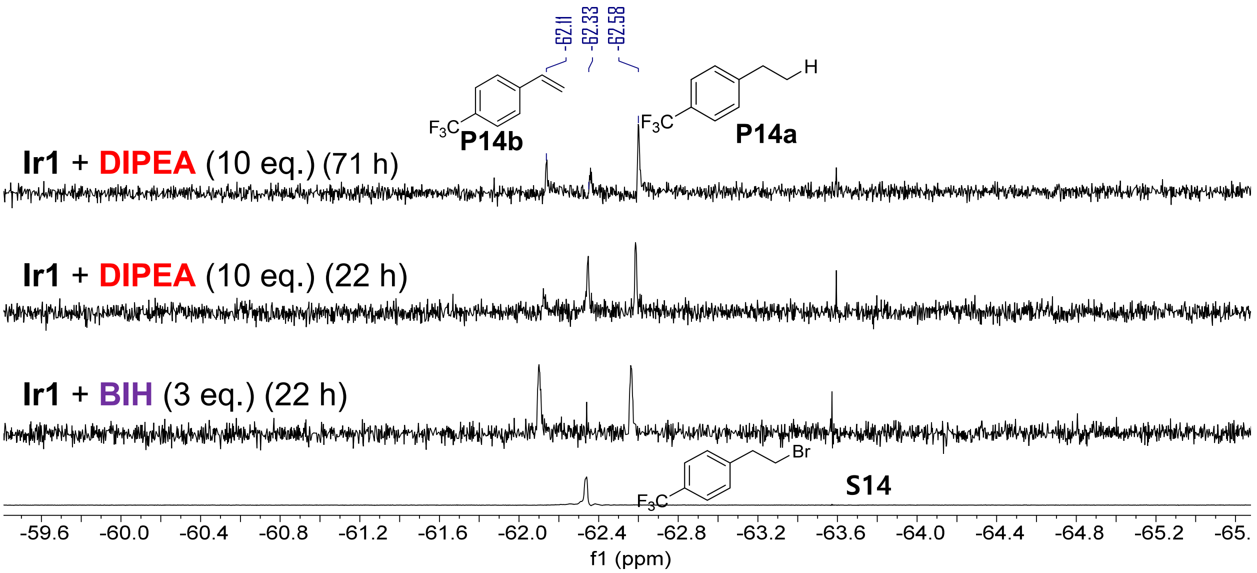

Figure S4. Optimization of the hydrodebromination of **S14**, with catalyst **Ir1**. The top plots show GC traces for reactions involving amine sacrificial reagents, and the bottom shows ^{19}F NMR spectra for reactions involving DIPEA or BIH as sacrificial reagents.

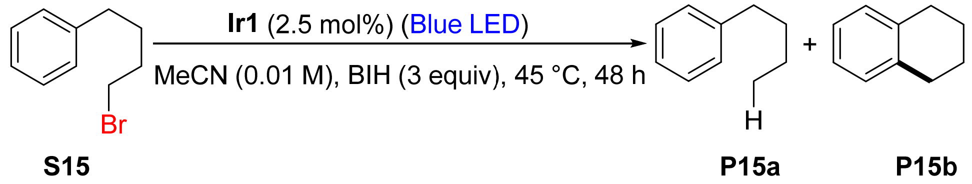

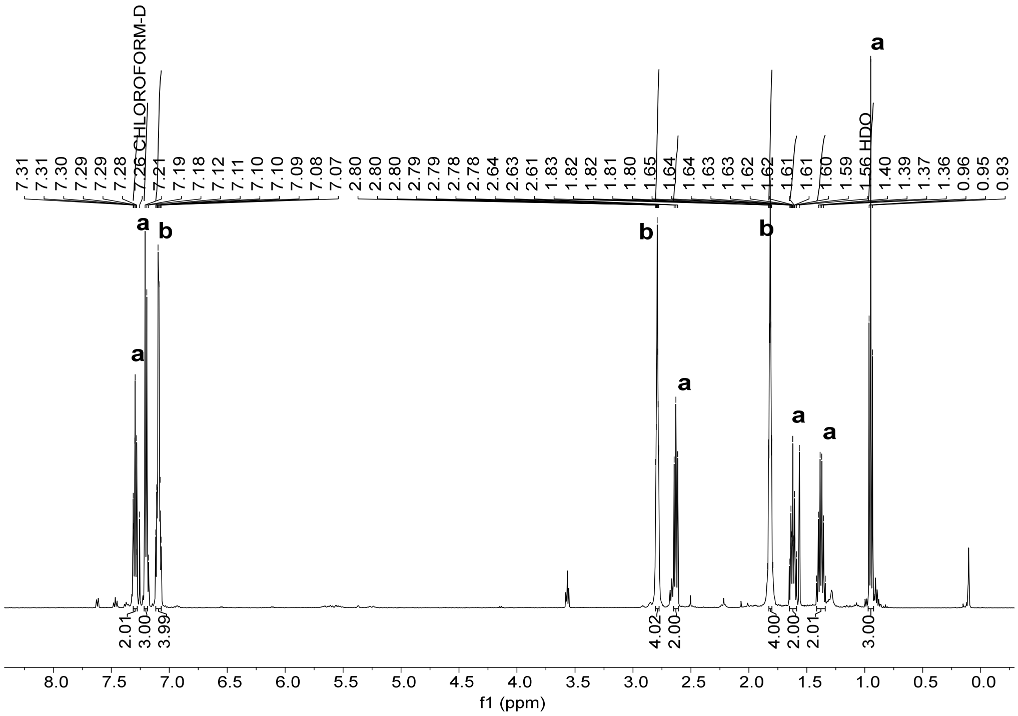

Figure S5. ^{1}H NMR spectrum of an isolated mixture of **P15a** and **P15b**.

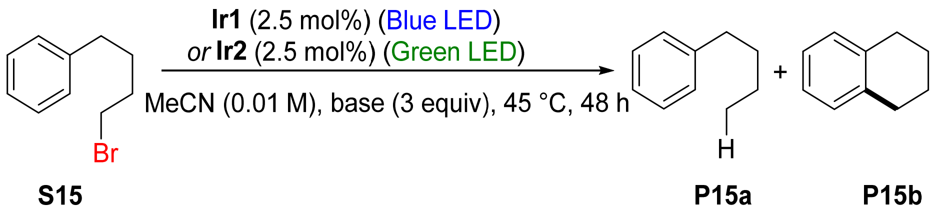

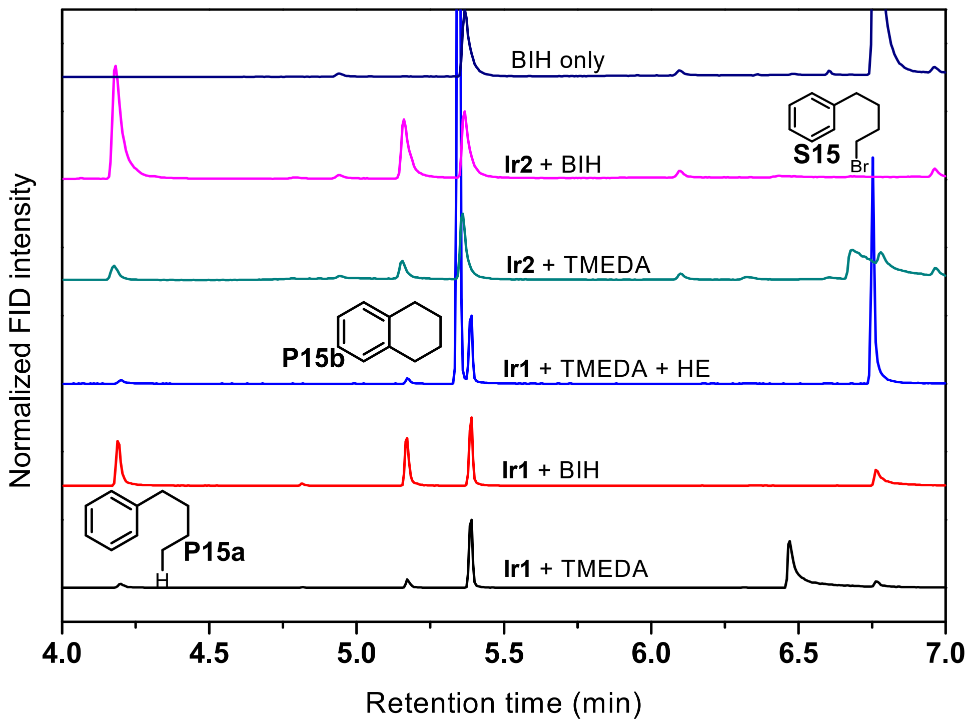

Figure S6. GC-FID traces for the optimization of the hydrodehalogenation of **S15**. (HE: Hantzsch ester, TMEDA: Tetramethylethylenediamine)

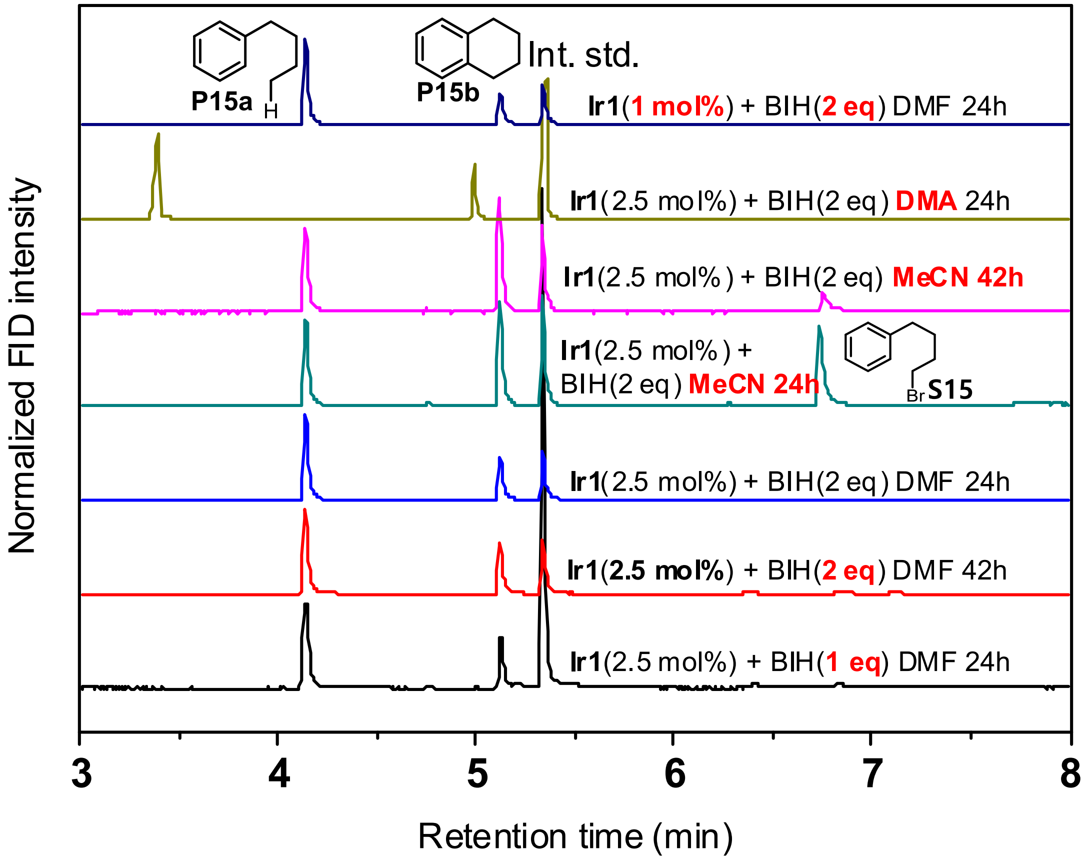

Figure S7. GC-FID traces for the optimization of the hydrodehalogenation of **S15**.

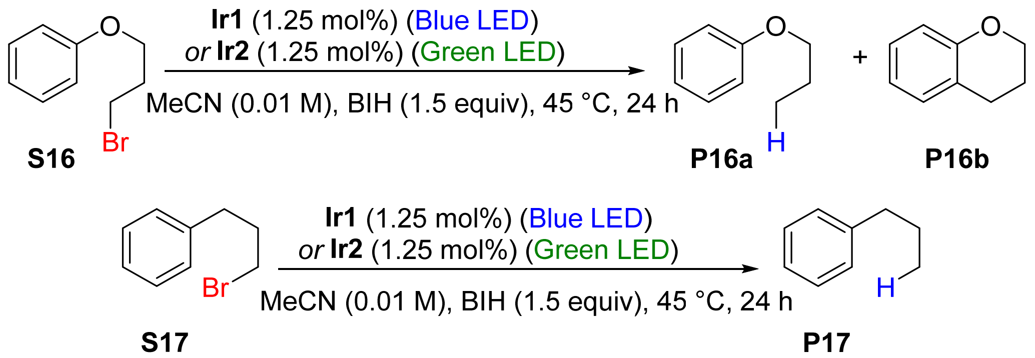

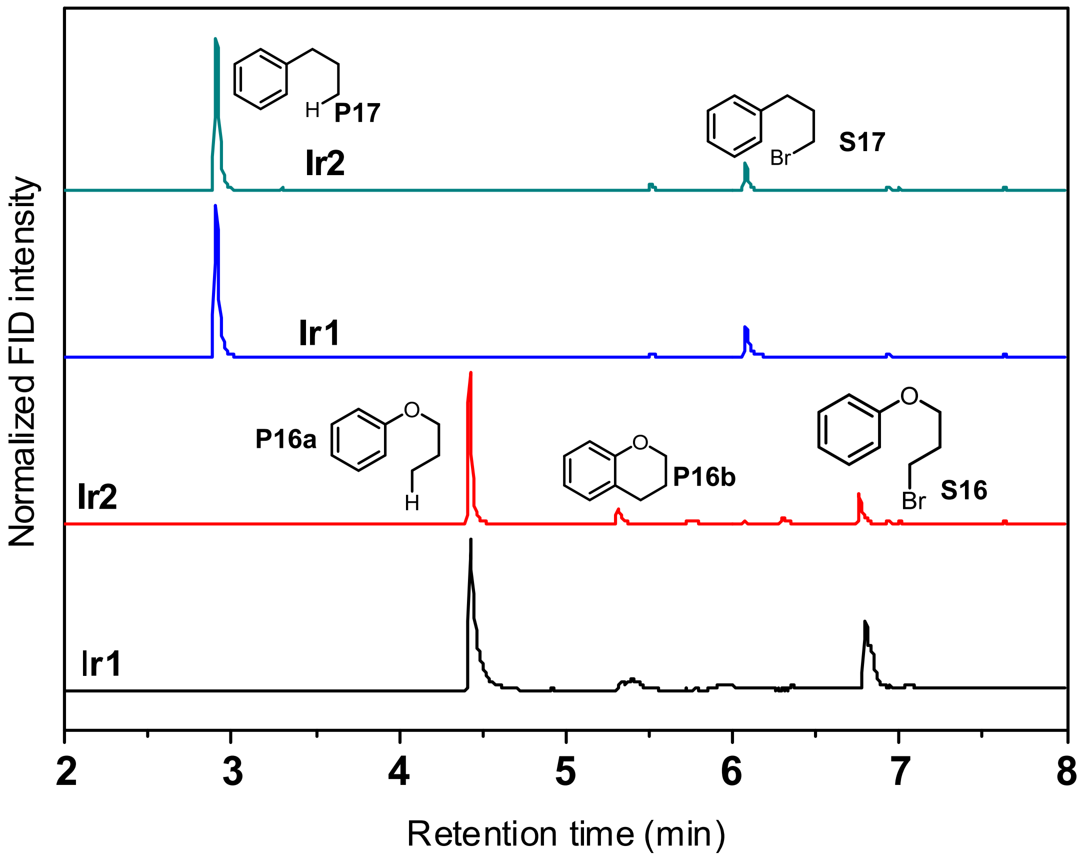

Figure S8. GC-FID traces for reactions of **S16** (bottom two traces) and **S17** (top two traces).

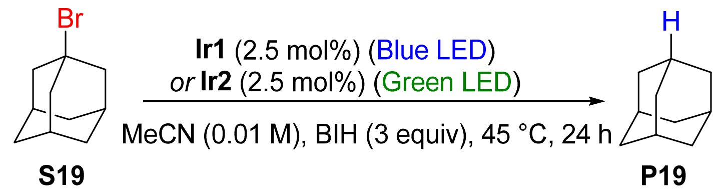

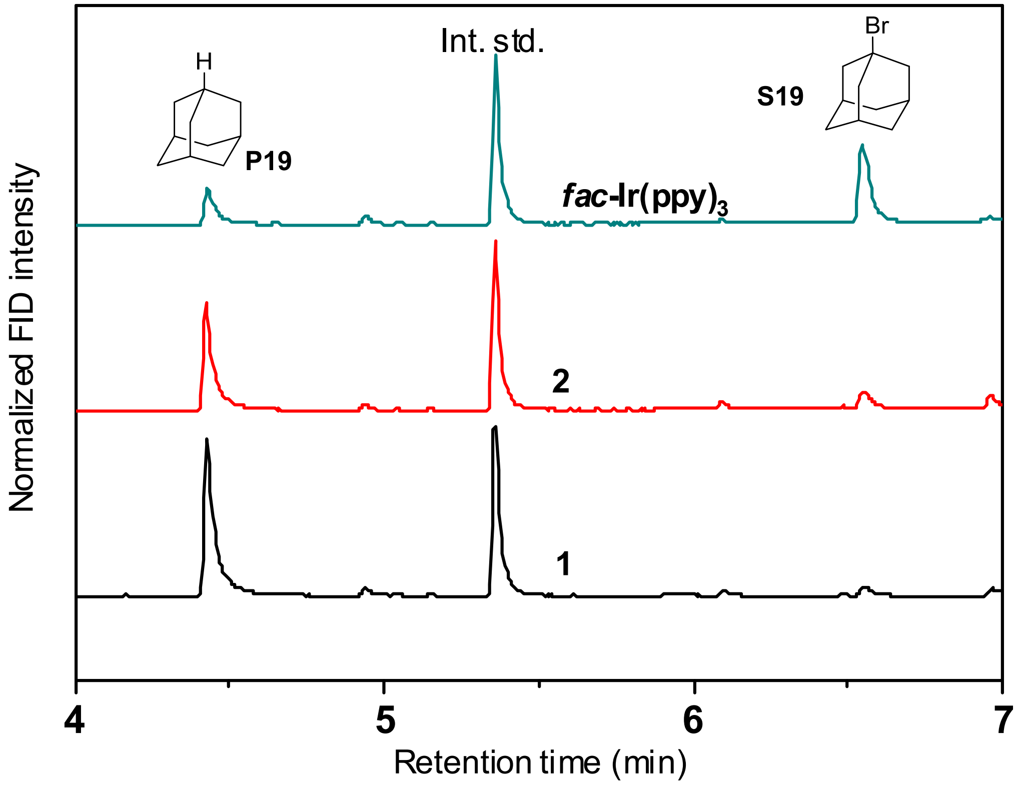

Figure S9. Reaction tracking of **S-B5** with a comparison of **1**, **2**, and *fac*-Ir(ppy)3.

Table S3. Reaction progress of prenylation on perfluoroarenes with the comparison of **Ir1**–**Ir8**.

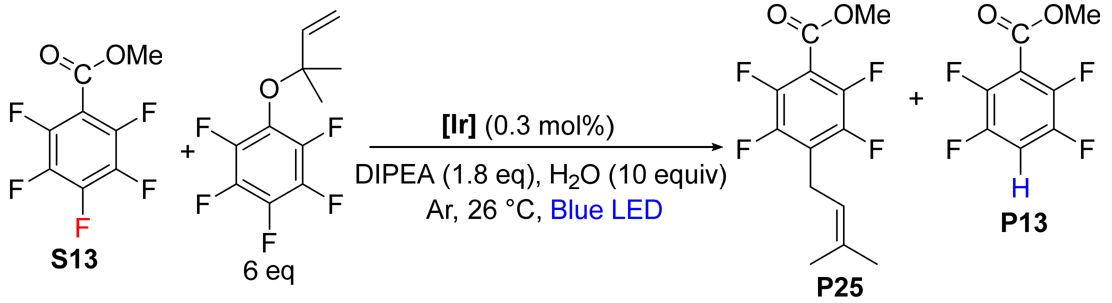

**[Ir]** catalyst	**Time (h)**	**S13%**	**P25%**	**P13%**
**Ir1**	22	78%	17%	5%
	68	78%	17%	5%
**Ir2**	22	41%	45%	14%
	68	41%	45%	14%
**Ir3**	22	79%	16%	5%
	68	79%	16%	5%
**Ir4**	22	96%	3%	1%
	68	96%	3%	1%
**Ir5**	22	39%	46%	15%
	68	39%	46%	15%
**Ir6**	22	71%	23%	6%
	68	51%	36%	13%
**Ir7**	22	95%	4%	1%
	68	93%	6%	1%
**Ir8**	22	85%	10%	5%
	68	81%	14%	5%

Experimental section

I. Materials

Reagents for photoredox reactions were measured and combined under an inert atmosphere inside a nitrogen-filled glovebox. Solvents were dried with 3 Å molecular sieves for overnight and deaerated with the freeze-pump-thaw method. All NMR solvents were dried with molecular sieves. Halide substrates (**S1–S19**), *N*-methylpyrrole, and benzophenone were obtained from commercial sources and used without further purification. All iridium photosensitizers, **Ir1**, **Ir2** and *fac*-Ir(ppy)$_3$, were synthesized according to the previously described procedure.[2,3] BIH[4,5] and other substrates (**S20–S22**,[6–8] and **S26**[9]) were prepared by following procedures from other reported literature. For hydrodefluorination reactions, all reagents were obtained from commercial suppliers (VWR, TCI Chemicals, and Oakwood Chemicals). Acetonitrile (CH_3CN) was dried for 48 h over activated 3 Å molecular sieves. Distilled diisopropylethylamine was stored over KOH pellets under an argon atmosphere in an amber bottle.

II. Physical methods

NMR spectra were recorded at room temperature using Oxford AS400, JEOL ECA-500, 600 NMR spectrometers. For hydrodefluorination experiments, NMR spectra were obtained on a 400 MHz Bruker Avance III spectrometer. UV–vis absorption spectra were recorded in DMF solutions in screw-capped quartz cuvettes using an Agilent Carey 60 UV–vis spectrophotometer. Emission spectra were recorded using a Horiba FluoroMax-5 spectrofluorometer with appropriate long-pass filters to exclude stray excitation light. Luminescence lifetimes were measured with a Horiba DeltaFlex lifetime instrument with a 453 nm pulsed diode excitation and an additional long-pass filter was used. The decay trace was fit using the instrument's analysis program.

III. General reaction procedure of photoredox catalysis

Procedure 1. Reaction screening: A 100 mL DMF stock solution was prepared with **Ir1**, **Ir2**, or *fac*-Ir(ppy)$_3$ and 1–3 equivalents of BIH (220–670 mg). A 1 mL aliquot of the stock solution was distributed into each reaction vial which had been previously charged with the substrate (1 equivalent, 10 μmol). The vial is sealed with a cap and is taken out from the glovebox for irradiation with LED light. The home-made reactor is surrounded by blue (465–470 nm) or green (520–525 nm) LED strip (purchased from Creativelighting LED Flex Ribbon Strips -12vdc, IP68 WP). The reaction progress was tracked with ^{19}F NMR or GC. ^{19}F NMR of the product was compared with a commercially available product molecule. GC-MS was used to identify the product molecule and the yield was calculated the GC-FID trace, comparing the integration to that the pure product, obtained commercially. For monitoring products via ^{19}F NMR, 1 or 2 equiv of fluorobenzene or trifluorotoluene were used as an internal standard, and for GC-FID experiments *n*-dodecane was used as an internal standard for quantification.

Procedure 2. Scale-up procedure for determining isolated yields (P15, P18, P20–P24): The reaction mixture was prepared in the same manner as described in Procedure 1, except that an appropriate volume of the stock solution was added to scale with an increased amount of the

substrate (100 μmol). After the reaction was terminated, the solution was treated with DI water to eliminate DMF and salt byproduct. The product was extracted with DCM several times and purified by column chromatography with ethyl acetate and n-hexane mixture as eluent (30% ethyl acetate to n-hexane).

Procedure 3. Hydrodefluorination and prenylation. Reactions were set up in a light bath which consists of high-intensity blue LEDs (λ_{max} emission ~ 450 nm) as described below. Blue LEDs (200 LEDs) were wrapped around the inner walls of the cylindrical metal container. The lid which was placed on the top of the bath made with holes such that reaction tubes were held firmly in the bath. The temperature of the bath was maintained at 26 °C using a cooling fan at the bottom of the metal container.

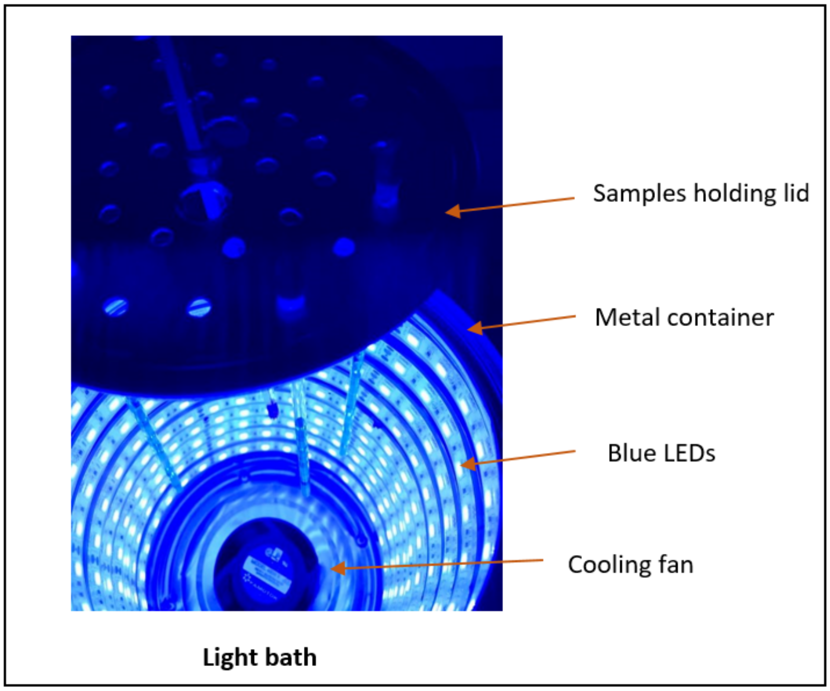

IV. Detailed procedures for photoredox catalysis

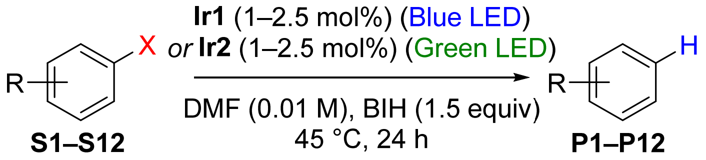

Synthesis of 1,3-Bis(trifluoromethyl)benzene (P1). The reaction was performed following Procedure 1 described above, using 1-bromo-3,5-bis(trifluoromethyl)benzene (**S1**) 1 mol% catalyst. The product yield was determined by ^{19}F NMR integration relative to an internal standard of 1-fluorobenzene. The product was not isolated due to its low boiling point.

Synthesis of trifluorotoluene (P2). The reaction was performed following Procedure 1 described above, using 4-bromobenzotrifluoride (**S2**, 1 mol% catalyst). The product yield was determined

by ^{19}F NMR integration relative to an internal standard of 1-fluorobenzene. The product was not isolated due to its low boiling point.

Synthesis of anisole (P3). The reaction was performed following Procedure 1 described above, using 3-bromoanisole (**S3**) with catalyst 1 mol% catalyst. The product yield was determined by GC-FID integration, using a calibration curve from an authentic sample of the product. The product was not isolated due to its low boiling point.

Synthesis of 1,3-dimethoxybenzene (P4). The reaction was performed following Procedure 1 described above, using 1-bromo-3,5-dimethoxybenzene (**S4**) with 1 mol% catalyst. The product yield was determined by GC-FID integration, using a calibration curve from an authentic sample of the product. The product was not isolated due to the low boiling point.

Synthesis of 1,3-difluorobenzene (P5 or **P6).** The reaction was performed following Procedure 1 described above, using 1-bromo-3,5-difluorobenzene (**S5**) or 1-bromo-2,4-difluorobenzene (**S6**) with 1 mol% catalyst. The product yield was determined by ^{19}F NMR integration relative to an internal standard of benzotrifluoride. The product was not isolated due to its low boiling point.

Synthesis of mesitylene (P7). The reaction was performed following Procedure 1 described above, using 2-bromomesitylene (**S7**) with 1 mol% catalyst. The product yield was determined by GC-FID integration, using a calibration curve from an authentic sample of the product. The product was not isolated due to its low boiling point.

Synthesis of 1,3,5-tri-*tert*-butyl-benzene (P8). The reaction was performed following Procedure 1 described above, using 2-bromo-1,3,5-tri-*tert*-butylbenzene (**S8**) with 1 mol% catalyst. The product yield was determined by GC-FID integration, using a calibration curve from an authentic sample of the product.

Synthesis of pentafluorobenzene (P9). The reaction was performed following Procedure 1 described above, using 1-chloro-2,3,4,5,6-pentafluorobenzene (**S9**) with 1 mol% catalyst. The product yield was determined by ^{19}F NMR integration relative to an internal standard of benzotrifluoride. The product was not isolated due to its low boiling point.

Synthesis of benzonitrile (P10 or **P11).** The reaction was performed following Procedure 1 described above, using 4-chlorobenzonitrile (**S10**) or 3-chlorobenzonitrile (**S11**) with 2.5 mol% catalyst. The product yield was determined by GC-FID integration, using a calibration curve from an authentic sample of the product. The product was not isolated due to its low boiling point.

Synthesis of methyl benzoate (P12). The reaction was performed following Procedure 1 described above, using methyl 4-chlorobenzoate (**S12**) with 2.5 mol% catalyst. The product yield was determined by GC-FID integration, using a calibration curve from an authentic sample of the product. The product was not isolated due to its low boiling point.

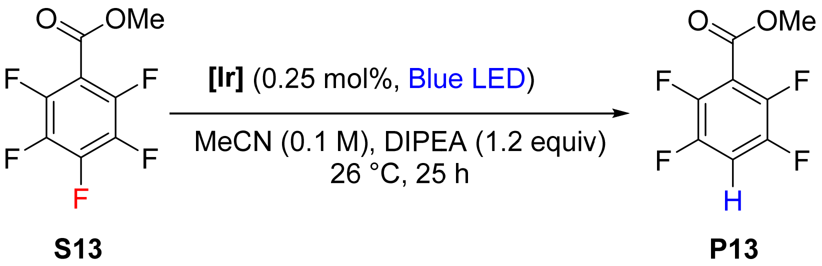

Synthesis of P13 via hydrodefluorination of 2,3,4,5,6-pentafluorobenzoate. A NMR tube fitted with a rubber septum was charged with **Ir cat** (0.25 mM, 1 mL in MeCN), methyl 2,3,4,5,6-pentafluorobenzoate (0.1 mmol, 22.6 mg, 15 µL, 1 equiv) and DIPEA (0.12 mmol, 15.5 mg, 21 µL, 1.2 equiv). Then the reaction mixture was degassed via Ar bubbling for 10 min and left under positive Ar pressure by removing the exit needle. The tube was placed in a light bath (description above) which was maintained at 26 °C. The reaction was monitored by ^{19}F NMR. Desired product signals were identified based on comparison with the literature values.[10] This reaction was carried out with Ir catalysts **Ir1-8**. Product conversion was determined by ^{19}F NMR (Table S2).

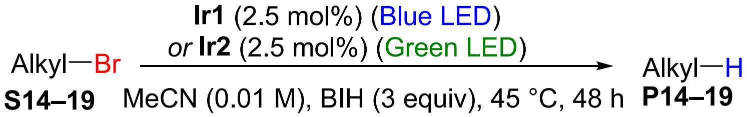

Synthesis of 1-ethyl-4-(trifluoromethyl)benzene (P14a) and 4-(trifluoromethyl)styrene (P14b). The reaction was performed following Procedure 2 described above, using 1-(2-bromoethyl)-4-(trifluoromethyl)benzene (**S14**) with 2.5 mol% catalyst. The product yields were determined by ^{19}F NMR integration relative to an internal standard of 1-fluorobenzene. The products were not isolated due to their low boiling points.

Synthesis of butylbenzene (P15a) and tetralin (P15b). The reaction was performed following Procedure 1 described above, using 1-bromo-4-phenylbutane (**S15**) with 2.5 mol% catalyst. The product was purified by column chromatography to afford a mixture of **P15a** and **P15b**. The final yield was determined by ^{1}H NMR integration. NMR peaks were matched with reported data. Butylbenzene: ^{1}H NMR (500 MHz, $CDCl_3$) δ 7.30 (dd, *J* = 8.3, 6.8 Hz, 2H), 7.20 (d, *J* = 7.5 Hz, 3H), 2.66 – 2.60 (m, 2H), 1.66 – 1.58 (m, 2H), 1.38 (h, *J* = 7.4 Hz, 2H), 0.95 (t, *J* = 7.4 Hz, 3H). Tetralin: ^{1}H NMR (500 MHz, $CDCl_3$) δ 7.09 (q, J = 5.2 Hz, 4H), 2.81 – 2.76 (m, 4H), 1.82 (p, J = 3.4 Hz, 4H).

Synthesis of propoxybenzene (P16a) and **chromane (P16b).** The reaction was performed following Procedure 1 described above, using 1-bromo-3-phenoxypropane (**S16**) with 1.25 mol% catalyst. The products were identified with GC-MS and quantified with GC-FID.

Synthesis of propylbenzene (P17). The reaction was performed following Procedure 1 described above, using 1-bromo-3-phenylpropane (**S17**) with 1.25 mol% catalyst. The product was identified with GC-MS and quantified with GC-FID.

Synthesis of Cbz-protected piperidine (P18). The reaction was performed following Procedure

2 described above, using Cbz-protected 4-bromopiperidine (**S18**) with 2.5 mol% catalyst. The product was purified with column chromatography with EA:Hex (30%) eluent. Isolated yields are 78–80%. ^{1}H NMR (400 MHz, $CDCl_3$) δ 7.37 – 7.26 (m, 5H), 5.12 (s, 2H), 3.46 – 3.41 (m, 4H), 1.59 – 1.52 (m, 6H). ^{13}C NMR (151 MHz, $CDCl_3$) δ 155.44, 137.12, 128.56, 127.98, 127.90, 77.35, 77.13, 76.92, 66.98, 44.95, 25.69, 24.46.

Synthesis of adamantane (P19). The reaction was performed following Procedure 1 described above, using 1-bromoadamantane (**S19**) with 2.5 mol% catalyst. The product was quantified with GC-FID.

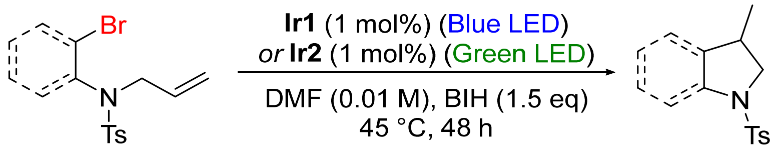

S20–22 **P20–22**

Synthesis of 3-methyl-1-tosylindoline (P20). The reaction was performed following Procedure 2 described above, using substrate **S20** with 1 mol% catalyst. The product was purified with column chromatography with EA:Hex (30%) eluent. Isolated yields are 61–65%. ^{1}H NMR (500 MHz, Acetonitrile-d_3) δ 7.67 (d, J = 8.0 Hz, 2H), 7.52 (d, J = 8.0 Hz, 1H), 7.29 (d, J = 8.1 Hz, 2H), 7.18 (t, J = 7.7 Hz, 1H), 7.09 (d, J = 7.4 Hz, 1H), 6.99 (t, J = 7.4 Hz, 1H), 4.10 – 4.05 (m, 1H), 3.40 (dd, J = 10.6, 7.1 Hz, 1H), 3.19 (h, J = 7.1 Hz, 1H), 2.33 (s, 3H), 1.04 (d, J = 6.8 Hz, 3H). ^{13}C NMR (151 MHz, $CDCl_3$) δ 144.11, 141.59, 136.92, 134.02, 129.72, 127.95, 127.42, 124.02, 123.87, 114.97, 77.33, 77.12, 76.91, 57.60, 34.74, 21.63, 19.59.

Synthesis of 3-methyl-1-tosylpyrrolidine (P21). The reaction was performed following Procedure 2 described above, using substrate **S22** with 1 mol% catalyst. The product was purified with column chromatography with EA:Hex (30%) eluent. Isolated yields are 63–65%. ^{1}H NMR (500 MHz, Acetonitrile-d_3) δ 7.72 – 7.62 (m, 2H), 7.38 (d, J = 8.0 Hz, 2H), 3.35 (dd, J = 9.8, 7.1 Hz, 1H), 3.25 (ddd, J = 9.9, 8.2, 4.2 Hz, 1H), 3.13 (ddd, J = 9.9, 8.2, 7.2 Hz, 1H), 2.66 (dd, J = 9.8, 7.8 Hz, 1H), 2.40 (s, 3H), 2.09 – 1.95 (m, 1H), 1.84 (dtd, J = 11.3, 7.0, 4.2 Hz, 1H), 1.27 (dq, J = 12.3, 8.3 Hz, 1H), 0.82 (d, J = 6.6 Hz, 3H). ^{13}C (126 MHz, $CDCl_3$) δ 143.38, 133.97, 129.71, 127.60, 77.42, 77.17, 76.92, 54.85, 47.71, 33.38, 33.31, 21.64, 17.73. All peaks match the reported spectra.[6]

Synthesis of 3,4-dimethyl-1-tosylpyrrolidine (P22). The reaction was performed following Procedure 2 described above, using substrate **S22** with 1 mol% catalyst. The product was purified with column chromatography with EA:Hex (30%) eluent. The two diastereomers eluted together in a 1:1 ratio. Isolated yields are 69–70%. ^{1}H NMR (600 MHz, $CDCl_3$) δ 7.69 (dd, J = 8.0, 2.9 Hz, 4H), 7.30 (d, J = 7.8 Hz, 4H), 3.49 (dd, J = 9.8, 6.6 Hz, 1H), 3.36 (dd, J = 9.7, 6.2 Hz, 3H), 2.93 (dd, J = 9.7, 5.3 Hz, 3H), 2.77 (t, J = 9.3 Hz, 1H), 2.41 (d, J = 2.6 Hz, 6H), 2.09 (dh, J = 13.1, 6.4 Hz, 3H), 1.58 – 1.52 (m, 1H), 0.88 (d, J = 5.9 Hz, 3H), 0.74 (d, J = 6.3 Hz, 9H). ^{13}C NMR (126 MHz, $CDCl_3$) δ 143.29, 134.18, 129.71, 129.67, 127.56, 127.52, 77.40, 77.15, 76.90, 55.07, 53.96,

40.56, 36.25, 21.64, 15.69, 12.95.

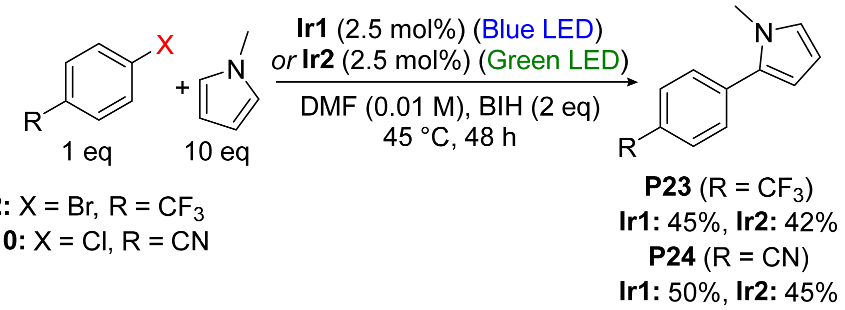

Synthesis of 1-methyl-2-(4-(trifluoromethyl)phenyl)-1H-pyrrole (P23). The reaction was performed following Procedure 2 described above, using 4-bromo-benzotrifluoride (**S2**) and 10 equivalents of *N*-methylpyrrole with 2.5 mol% catalyst. The product was purified with column chromatography with EA:Hex (30%) eluent. Isolated yields are 42–45%. ^{1}H NMR (400 MHz, $CDCl_3$): 7.56 (d, J = 8.0 Hz, 2H), 7.44 (d, J = 8.1 Hz, 2H), 6.69 (s, 1H), 6.22 (s, 1H), 6.14 (t, J = 3.3 Hz, 1H), 3.63 (s, 3H). ^{19}F NMR (235 MHz): −62.3 All peaks match the reported spectra.[11]

Synthesis of 4-(1-methyl-1H-pyrrol-2-yl)benzonitrile (P24). The reaction was performed following Procedure 2 described above, using 4-chloro-benzonitrile (**S10**) and 10 equivalents of *N*-methylpyrrole with 2.5 mol% catalyst. The product was purified with column chromatography with EA:Hex (30%) eluent. Isolated yields are 45–50%. ^{1}H NMR (600 MHz, $CDCl_3$) δ 7.69–7.64 (m, 2H), 7.49 (d, J = 8.2 Hz, 2H), 6.78 (t, J = 2.3 Hz, 1H), 6.37–6.32 (m, 1H), 6.24–6.20 (m, 1H), 3.71 (s, 3H). ^{13}C NMR (151 MHz, $CDCl_3$) δ 137.78, 132.72, 132.38, 128.38, 125.97, 119.19, 110.84, 109.75, 108.68, 77.35, 77.14, 76.93, 35.60. All peaks match the reported spectra.[12]

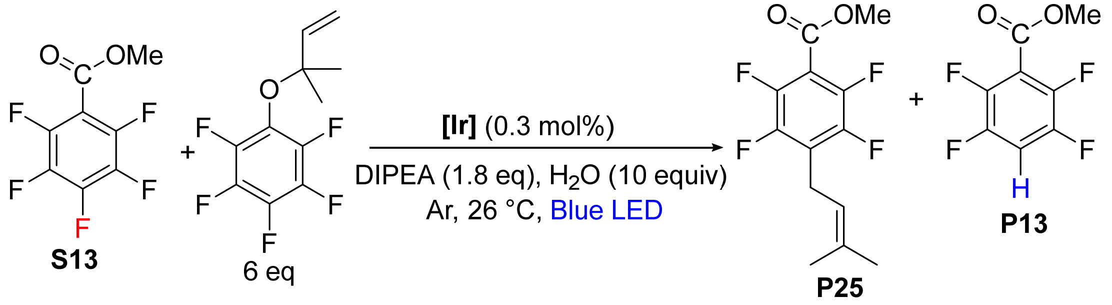

Synthesis of P25 via photocatalytic prenylation on perfluoroarenes. A NMR tube fitted with a rubber septum was charged with **Ir cat** (0.3 mM, 1 mL in MeCN), methyl 2,3,4,5,6-pentafluorobenzoate (0.1 mmol, 22.6 mg, 15 µL, 1 equiv), DIPEA (0.18 mmol, 23.2 mg 31.4 µL, 1.8 equiv), DI water (1 mmol, 18 mg, 18 µL, 10 equiv) and 1,2,3,4,5-pentafluoro-6-((2-methylbut-3-en-2-yl)oxy)benzene (0.6 mmol, 151 mg, 118 µL, 6 equiv). Then the reaction mixture was degassed via Ar bubbling for 10 min and then left under positive Ar pressure by removing the exit needle. The tube was placed in a light bath (description above) which was maintained at 26 °C.

The reaction was monitored by ^{19}F NMR. Desired product signals were identified by comparison with the literature values.[13] This reaction was carried out with Ir catalyst **Ir1-8**. Product conversion was determined by ^{19}F NMR (Table S3).

Degradation of the lignin model substrate S26. The reaction was performed following Procedure 1 described above, using the lignin model substrate **S26** with 1 mol% catalyst and 2 equiv BIH. The products were identified with GC-MS and quantified with GC-FID.

Synthesis of diphenylmethanol (P27). The reaction was performed following Procedure 1 described above, using benzophenone (**S27**) with 1 mol% catalyst and 2 equiv BIH. The product was identified with GC-MS and quantified with GC-FID.

V. Stern-Volmer quenching experiments

Time-resolved quenching experiment

Procedure: Stock solutions of **Ir1** or *fac*-Ir(ppy)$_3$ (1 mM in DMF) and the quencher were prepared. The cuvette was filled with 3 mL of dry DMF and 100 μL of the stock solution of the iridium complex was added to the cuvette. After collecting the photoluminescence lifetime of the solution, 5 uL aliquots of the BIH solution were, and the lifetime was measured after each aliquot.

Quenching rate calculation

Ir1 with BIH:

Volume added (L)	[BIH] in cuvette (M)	Measured lifetime (s)	τ_0/τ_Q
0	0	7.85×10^{-7}	1.00
5.00×10^{-6}	0.0000833	7.77×10^{-7}	1.01
1.00×10^{-5}	0.000166	7.66×10^{-7}	1.02
1.50×10^{-5}	0.000249	7.57×10^{-7}	1.04
2.00×10^{-5}	0.000331	7.38×10^{-7}	1.06

Ir1 with **S10**:

Volume added (L)	[**S10**][a] in cuvette (M)	Measured lifetime (s)	τ_0/τ_Q
0	0	7.25×10^{-7}	1.00
5.00×10^{-6}	0.000311	6.97×10^{-7}	1.04
1.00×10^{-5}	0.000623	6.87×10^{-7}	1.05
1.50×10^{-5}	0.000934	6.78×10^{-7}	1.07
2.00×10^{-5}	0.00124	6.69×10^{-7}	1.08

[a]**S10** = 4-chlorobenzonitrile

Ir1 with **S7**:

Volume added (L)	[**S7**][a] in cuvette (M)	Measured lifetime (s)	τ_0/τ_Q
0	0	7.23×10^{-7}	1.00
5.00×10^{-6}	0.000218	6.84×10^{-7}	1.06
1.00×10^{-5}	0.000436	6.68×10^{-7}	1.08
1.50×10^{-5}	0.000653	6.57×10^{-7}	1.10

[a]**S7** = 2-bromomesitylene

fac-$Ir(ppy)_3$ with BIH:

Volume added (L)	[BIH] in cuvette (M)	Measured lifetime (s)	τ_0/τ_Q
0	0	1.52×10^{-6}	1
5.00×10^{-6}	0.000083	1.50×10^{-6}	1.01
1.00×10^{-5}	0.000166	1.47×10^{-6}	1.03
1.50×10^{-5}	0.000249	1.44×10^{-6}	1.06
2.00×10^{-5}	0.000333	1.41×10^{-6}	1.08
2.50×10^{-5}	0.000416	1.39×10^{-6}	1.09

fac-$Ir(ppy)_3$ with **S10**:

Volume added (L)	[**S10**][a] in cuvette (M)	Measured lifetime (s)	τ_0/τ_Q
0	0	1.50×10^{-6}	1
5.00×10^{-6}	0.000311	1.47×10^{-6}	1.02
1.00×10^{-5}	0.000623	1.44×10^{-6}	1.04
1.50×10^{-5}	0.000934	1.40×10^{-6}	1.07
2.00×10^{-5}	0.001245	1.36×10^{-6}	1.10
2.50×10^{-5}	0.001557	1.34×10^{-6}	1.12

[a]**S10** = 4-chlorobenzonitrile

fac-$Ir(ppy)_3$ with **S7**:

Volume added (L)	[**S7**][a] in cuvette (M)	Measured lifetime (s)	τ_0/τ_Q
0	0	1.51×10^{-6}	1
5.00×10^{-6}	0.000218	1.37×10^{-6}	1.10
1.00×10^{-5}	0.000435	1.31×10^{-6}	1.15
1.50×10^{-5}	0.000653	1.28×10^{-6}	1.18
2.00×10^{-5}	0.000871	1.24×10^{-6}	1.22
2.50×10^{-5}	0.001089	1.21×10^{-6}	1.25

[a]**S7** = 2-bromomesitylene

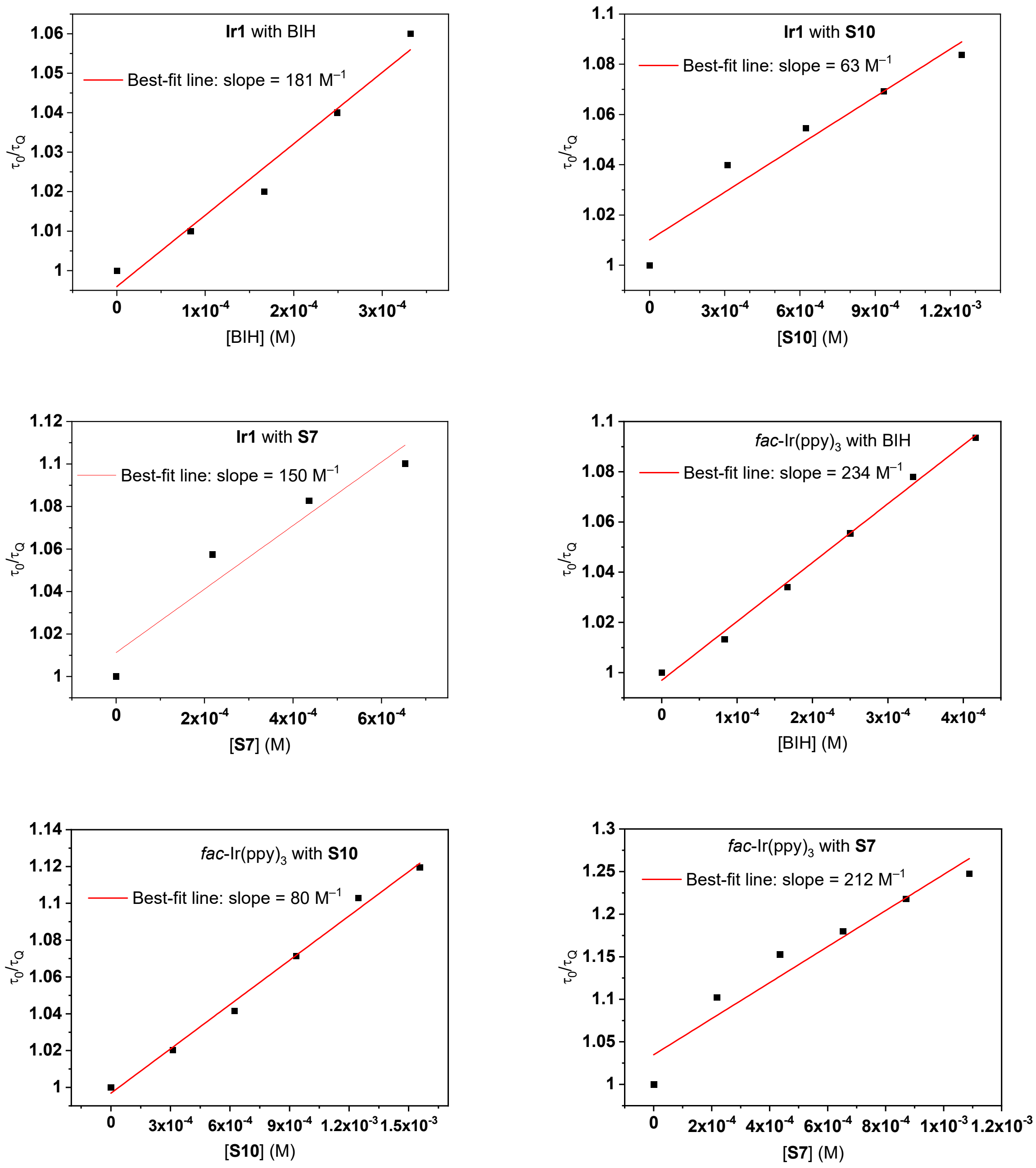

	**Ir1**		*fac*-Ir(ppy)$_3$	
**Quencher**	Slope (k_q/k_0)[a]	k_q (M^{-1} s^{-1})	Slope (k_q/k_0)[a]	k_q (M^{-1} s^{-1})
BIH	181	2.3×10^8	234	1.5×10^8
**S10**	63	8.1×10^7	80	5.3×10^7
**S7**	150	1.9×10^8	212	1.4×10^8

[a] $k_0 = k_{nr} + k_r = 1/\tau_0$

Figure S10. Stern-Volmer quenching plots for BIH, 4-chlorobenzonitrile (**S10**), and 2-bromomesitylene (**S7**). Table showing the calculation of quenching rates.

VI. Photochemical quantum yield measurement

Photon flux calibration

Ferrioxalate actinometry[14] was used to calibrate the intensity of the LED light source. The actinometer, $K_3[Fe(C_2O_4)_3]$, is reduced following excitation by accepting an electron from oxalate. The overall reaction that occurs following irradiation is:

$$[Fe(C_2O_4)_3]^{3-} + h\nu \longrightarrow Fe^{2+} + C_2O_4^{\bullet -} + 2\,C_2O_4^{2-}$$

$$[Fe(C_2O_4)_3]^{3-} + C_2O_4^{\bullet -} \longrightarrow Fe^{2+} + 2\,CO_2 + 3\,C_2O_4^{2-}$$

$$\textit{simplified: } 2\,Fe^{3+} + C_2O_4^{2-} \xrightarrow{h\nu} 2\,Fe^{2+} + 2\,CO_2$$

Generation of the ferrous ion can be detected by introducing an excess amount of 1,10-phenanthroline, which forms the complex $[Fe(phen)_3]^{2+}$. $[Fe(phen)_3]^{2+}$ has a strong absorption at 510 nm with an extinction coefficient of 11,100 M^{-1} cm^{-1}. The reported quantum yield for each excitation wavelength is tabulated in the literature.[14]

The photon flux can be determined by using the following equation:

$$I = \frac{AV_2V_3}{\varepsilon d \phi_{ex} t V_1}$$

Where

I = photon flux (einsteins min^{-1} $sample^{-1}$)

A = absorbance (λ = 510 nm) or ΔA = corrected absorbance at 510 nm (absorption difference before and after irradiation). ΔA = 1.97 for this measurement.

V_1 = volume (mL) of aliquot which was taken from irradiated solution, 1 mL for this study.

V_2 = volume (L) of an initially irradiated actinometric solution, 0.00300 L.

V_3 = total volume (mL) of solution following dilution of V_1 (volume when absorbance was measured), 3.50 mL

ε = extinction coefficient of $[Fe(phen)_3]^{2+}$ at 510 nm, known as 1.11×10^4 M^{-1} cm^{-1}

d = path length of cuvette to measure absorbance, 1 cm.

ϕ_{ex} = Quantum yield for ferrioxalate photoreduction at the specified excitation wavelengths, determined to be 0.879. The 34 W Blue LED lamps: Kessil KSH150B light source used for these experiments emits two distinct wavelengths (419 and 460 nm) and QY is calculated as the average of the QYs at each wavelength, weighted by the relative light intensity at each wavelength. (I(419) : I(460) = 1:1.2)

t = irradiated time (min). 15 sec (0.25 min) for this study.

The calculated photon flux (I) in einsteins min^{-1} $sample^{-1}$ is 8.11×10^{-6} which is obtained with 309 mA current (34 W Blue LED lamps: Kessil KSH150B under 110 V applied potential.) and 3 cm^2 of irradiated area.

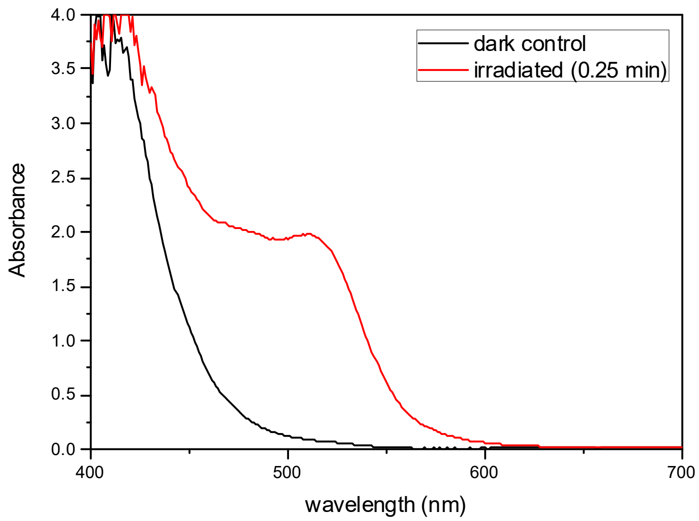

Figure S11. Detection of $[Fe(phen)_3]^{2+}$ by measuring absorbance after 0.25 min of irradiation

<u>Photochemical quantum yield (PCQY) of hydrodebromination of 4-bromo-benzotrifluoride</u>

The same reaction set up used for this measurement. The reaction solution contains 32 µL (0.23 mmol) of 4-bromo-benzotrifluoride, **Ir1** (1 mol% relative to substrate), 1 equivalent of BIH (sacrificial reagent) and 1 equivalent of 1-fluorobenzene as a ^{19}F NMR reference, added to a cuvette filled with 3 mL of dried DMF. The solution was irradiated for 60 min with the same light used in the photon flux measurement described above. The rate of conversion was tracked by ^{19}F NMR (GC-FID for 2-bromomesitylene), using the internal reference to determine product yield. The reaction yield after 1 h irradiation was 10% and the rate of substrate consumption per minute is 3.81×10^{-7} mole min^{-1} $sample^{-1}$

The PCQY can be determined by the following equation:

$$\Phi = \frac{\text{Rate of decay of substrate (moles/min)}}{\text{Total photon flux (moles/min)} \times \text{f}}$$

$$f = 1 - 10^{-A(419nm)}$$

Absorbed photon flux is calculated by considering the time and the fraction factor, f (Absorbance at 419 nm of **Ir1** in the solution is 3.47 which is calculated from the extinction coefficient of 4568 M^{-1} cm^{-1} and concentration of 0.76 mM. The absorbance at 460 nm was low and was not

considered). Finally, the obtained obtained PCQY is 4.7×10^{-2} (4.7%). With the same conditions, 3.0% of mesitylene product is obtained from 2-bromomesitylene at 25 °C, with 6.0% yield at 45 °C. The PCQYs are respectively 1.4% and 2.8% for the hydrodebromination of 2-bromomesitylene.

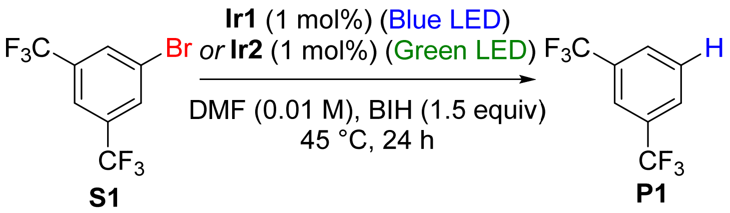

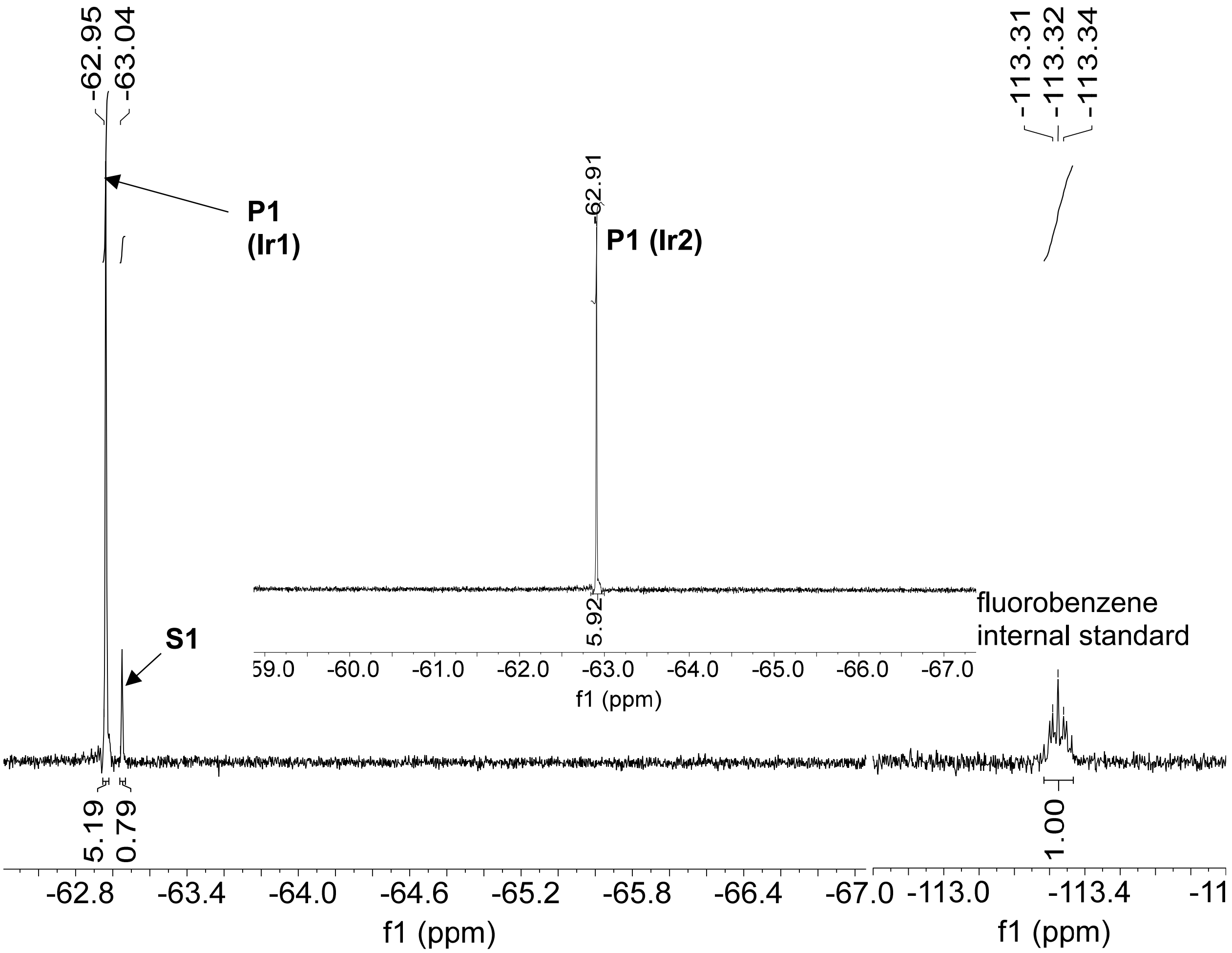

Figure S12. ^{19}F NMR spectra for the hydrodebromination of **S1.**

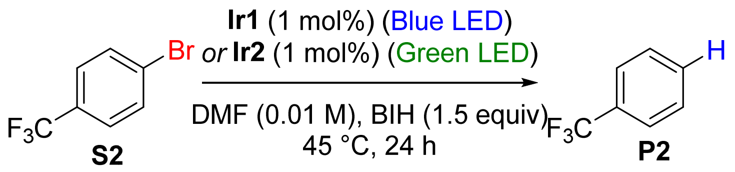

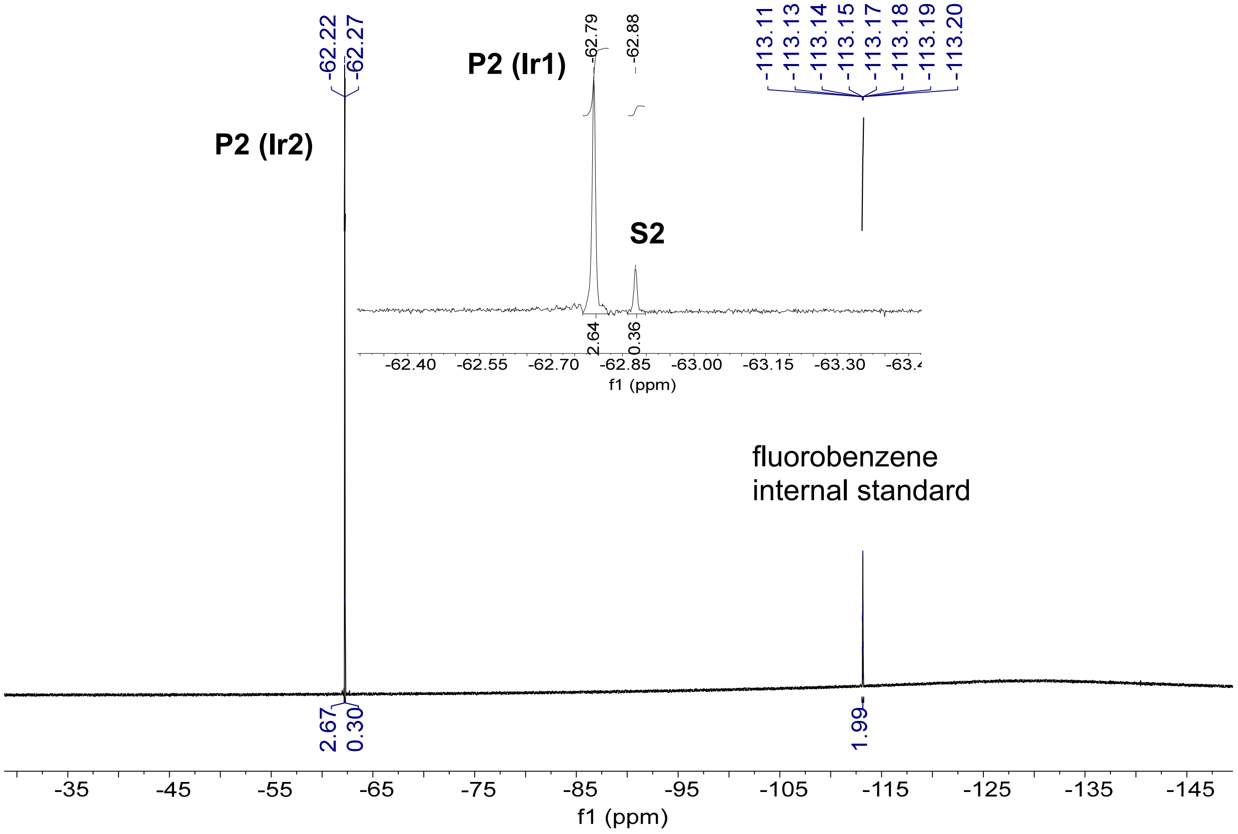

Figure S13. ^{19}F NMR spectra for the hydrodebromination of **S2.**

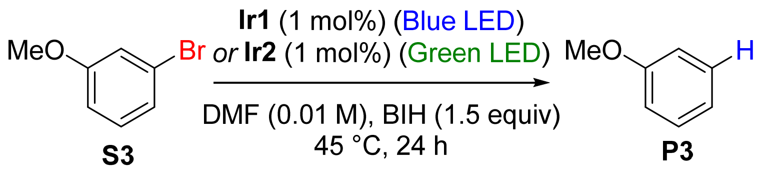

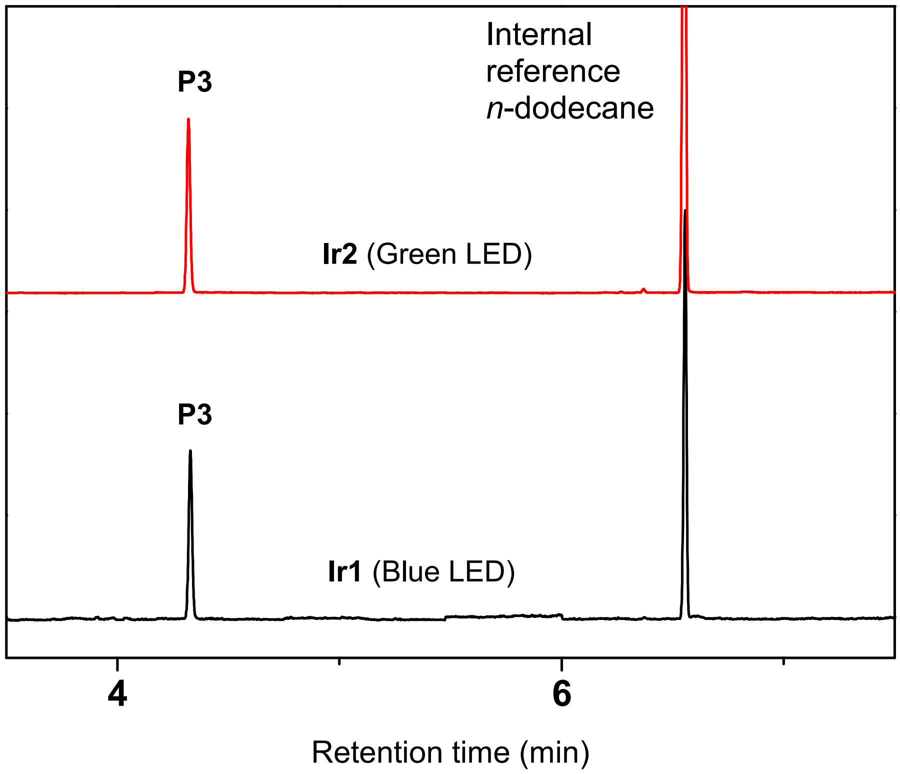

Figure S14. GC-FID traces for the hydrodebromination of **S3.**

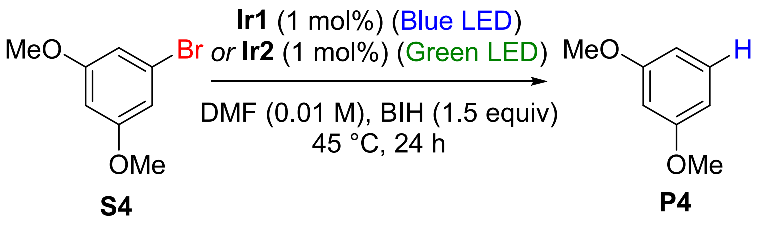

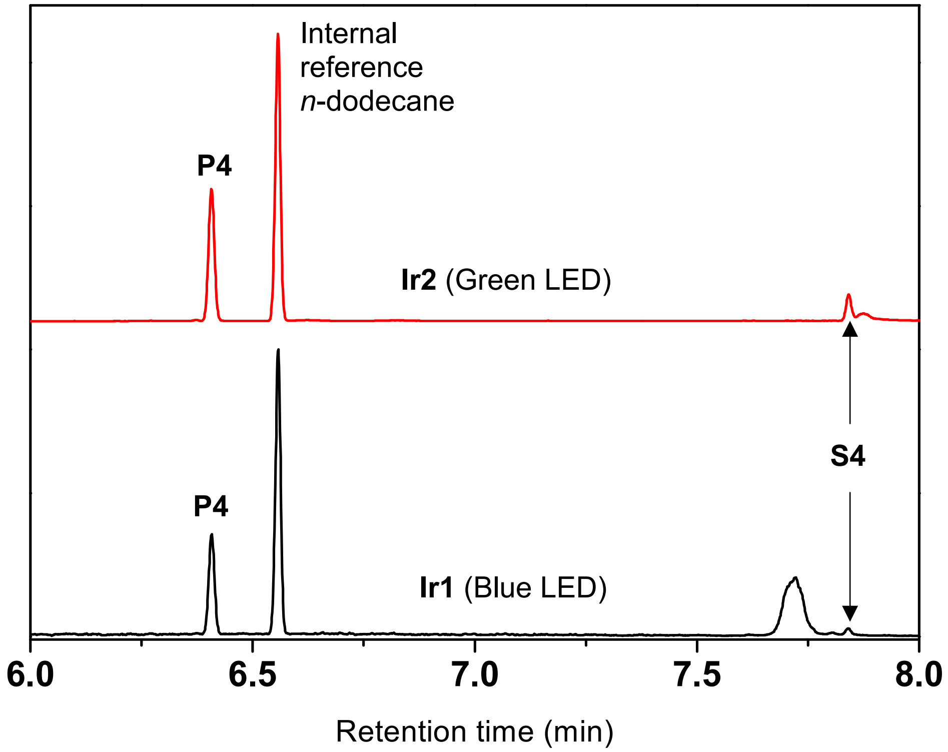

Figure S15. GC-FID traces for the hydrodebromination of **S4.**

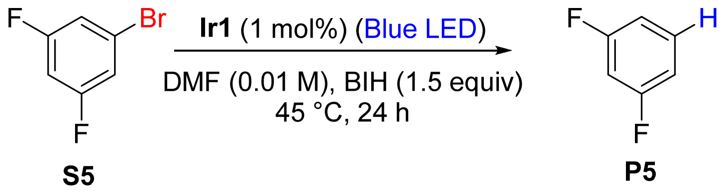

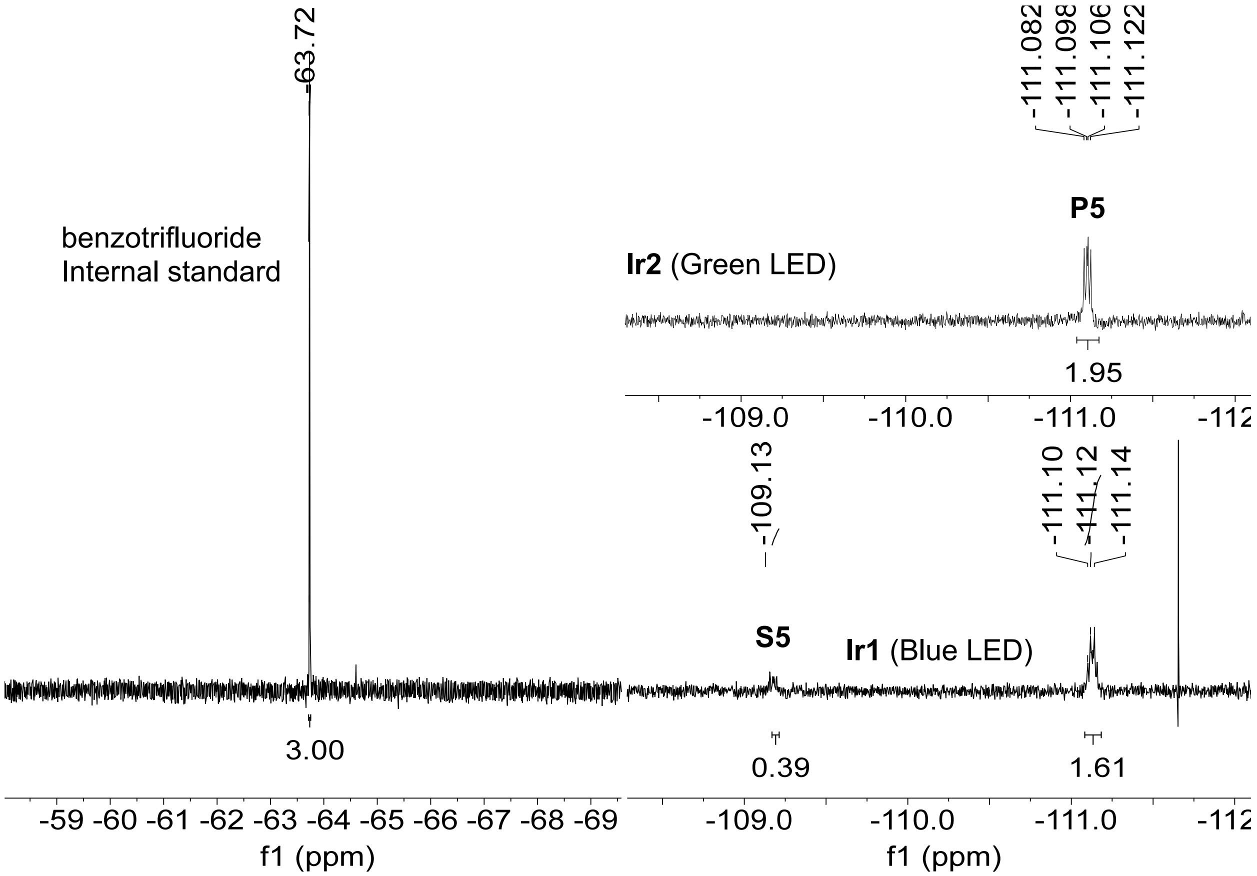

Figure S16. ^{19}F NMR spectrum for the hydrodebromination of **S5.**

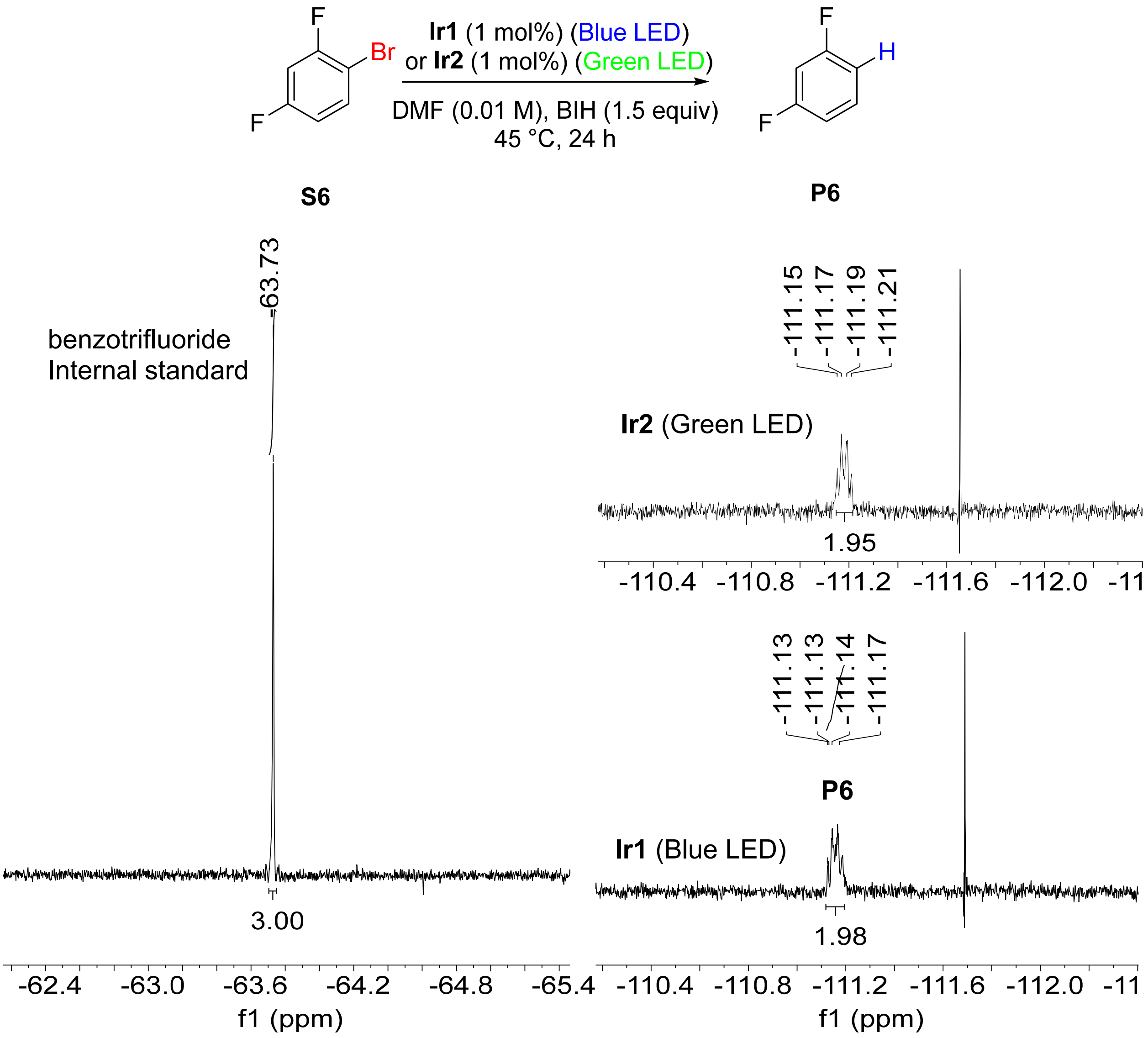

Figure S17. ^{19}F NMR spectrum for the hydrodebromination of **S6.**

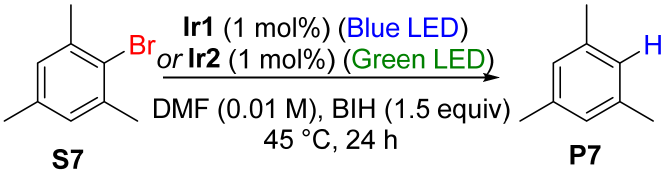

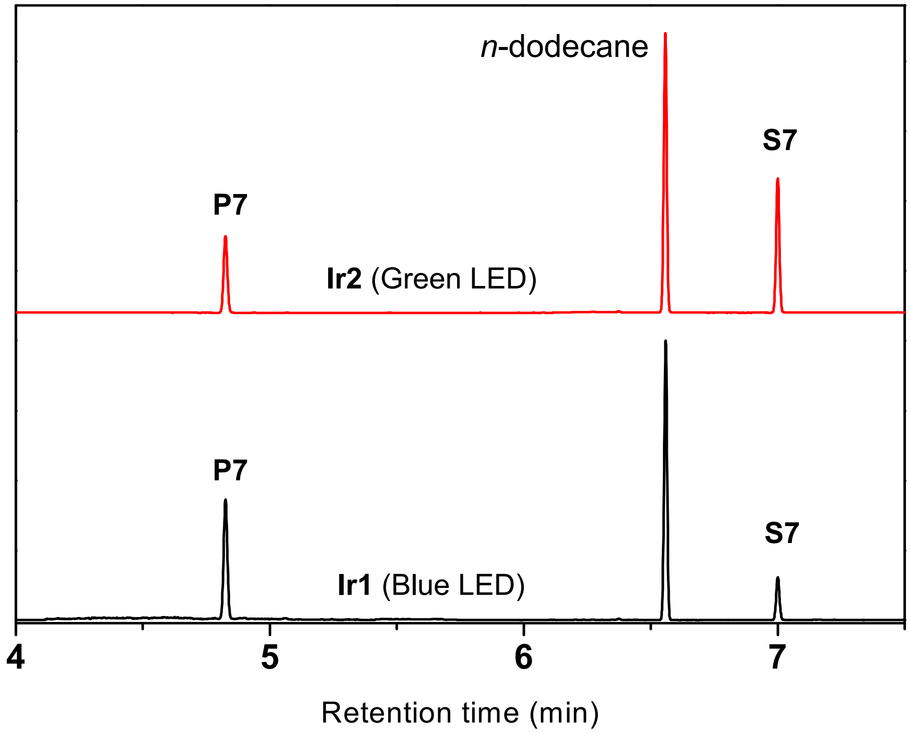

Figure S18. GC-FID traces for the hydrodebromination of **S7.**

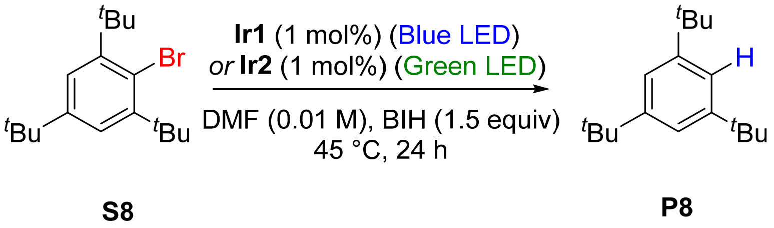

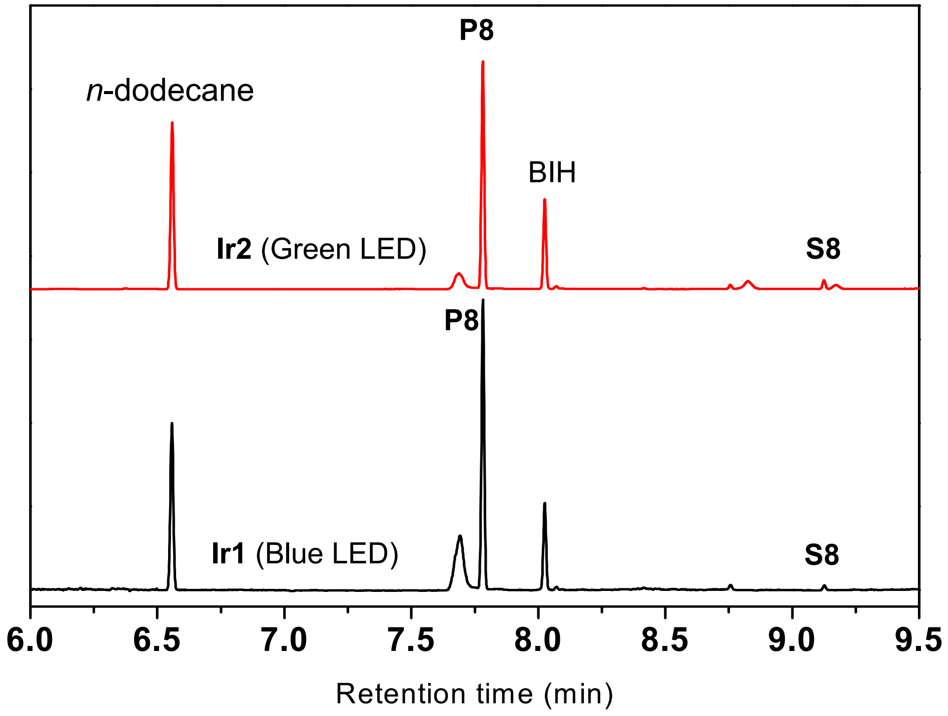

Figure S19. GC-FID traces for the hydrodebromination of **S8.**

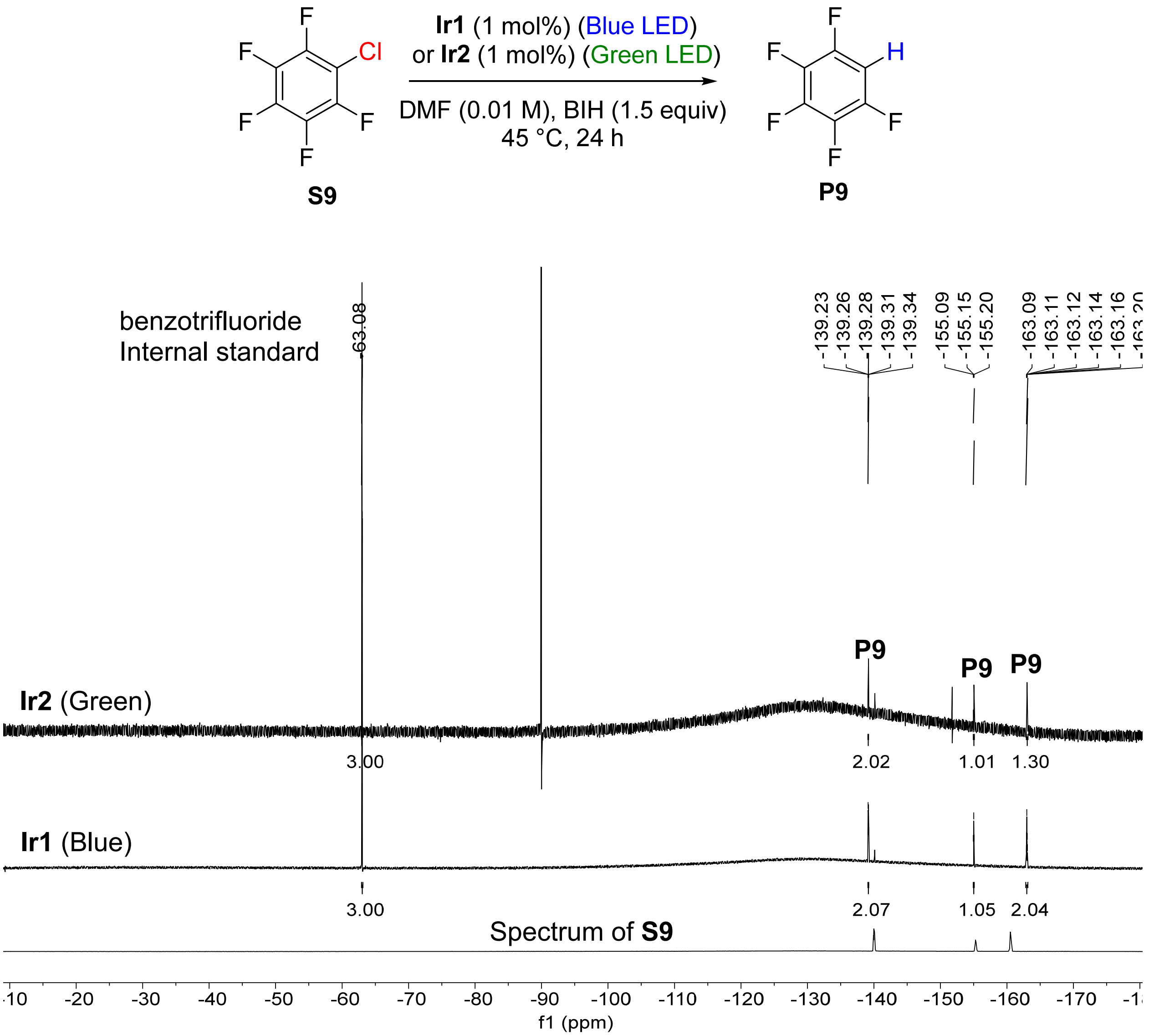

Figure S20. ^{19}F NMR spectrum for the hydrodechlorination of **S9.**

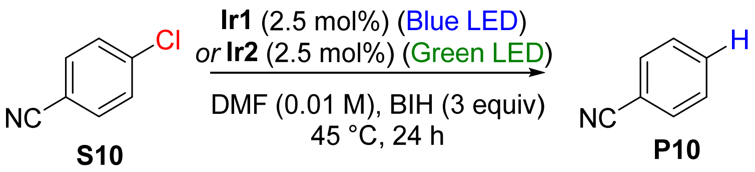

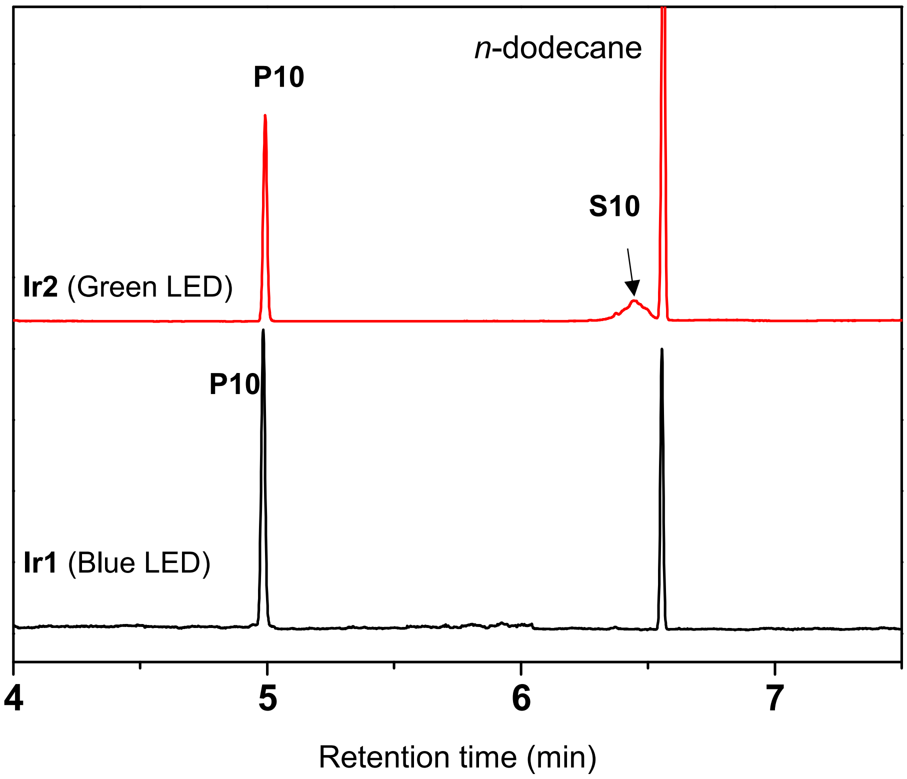

Figure S21. GC-FID traces for the hydrodebromination of **S10.**

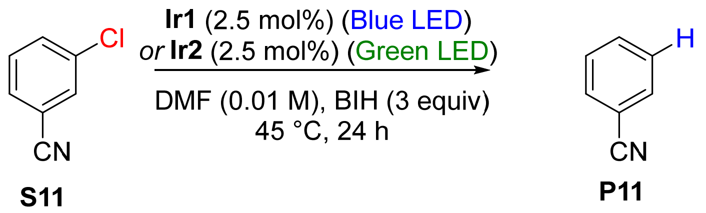

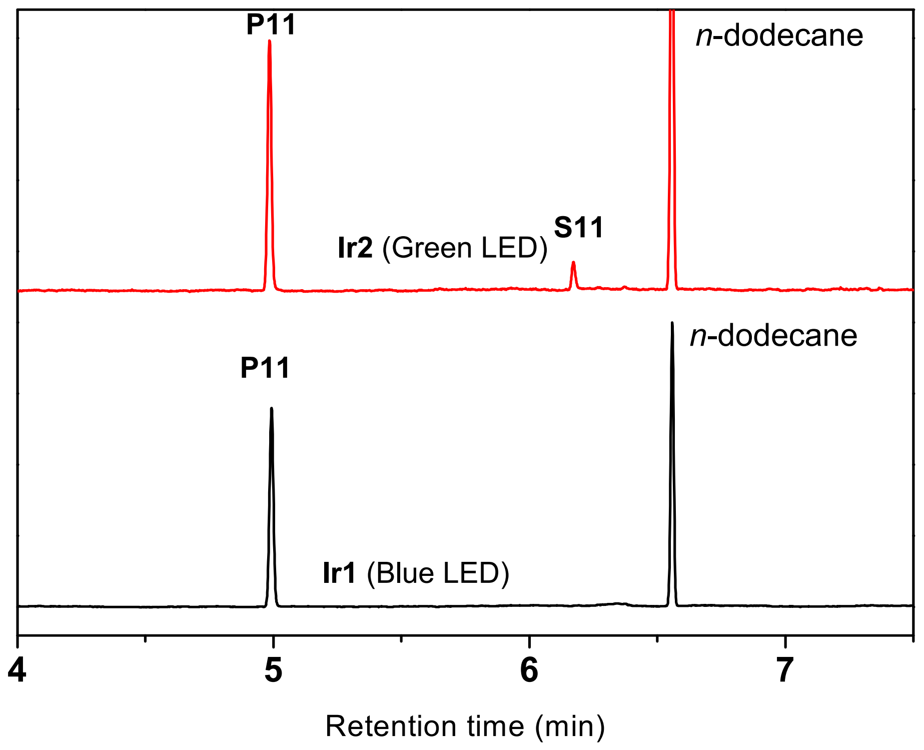

Figure S22. GC-FID traces for the hydrodebromination of **S11.**

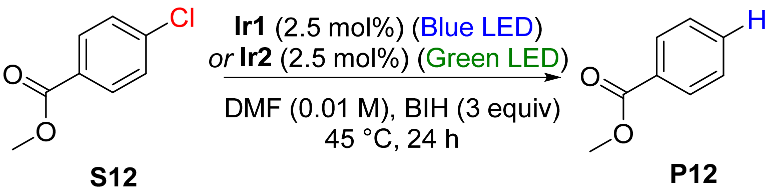

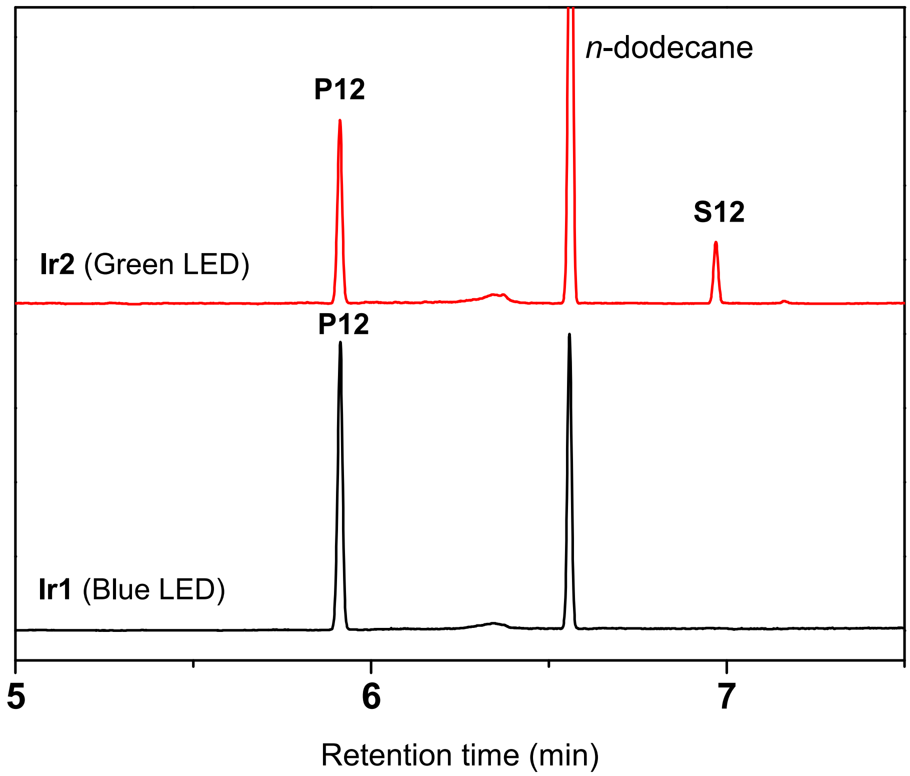

Figure S23. GC-FID traces for the hydrodebromination of **S12.**

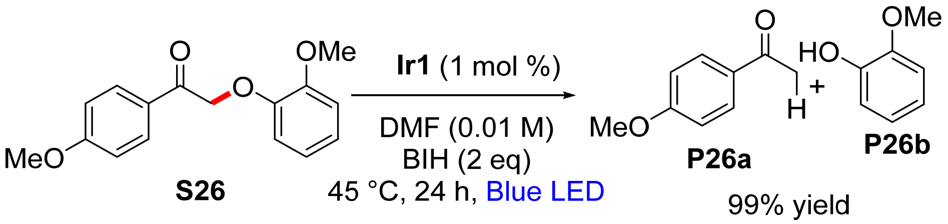

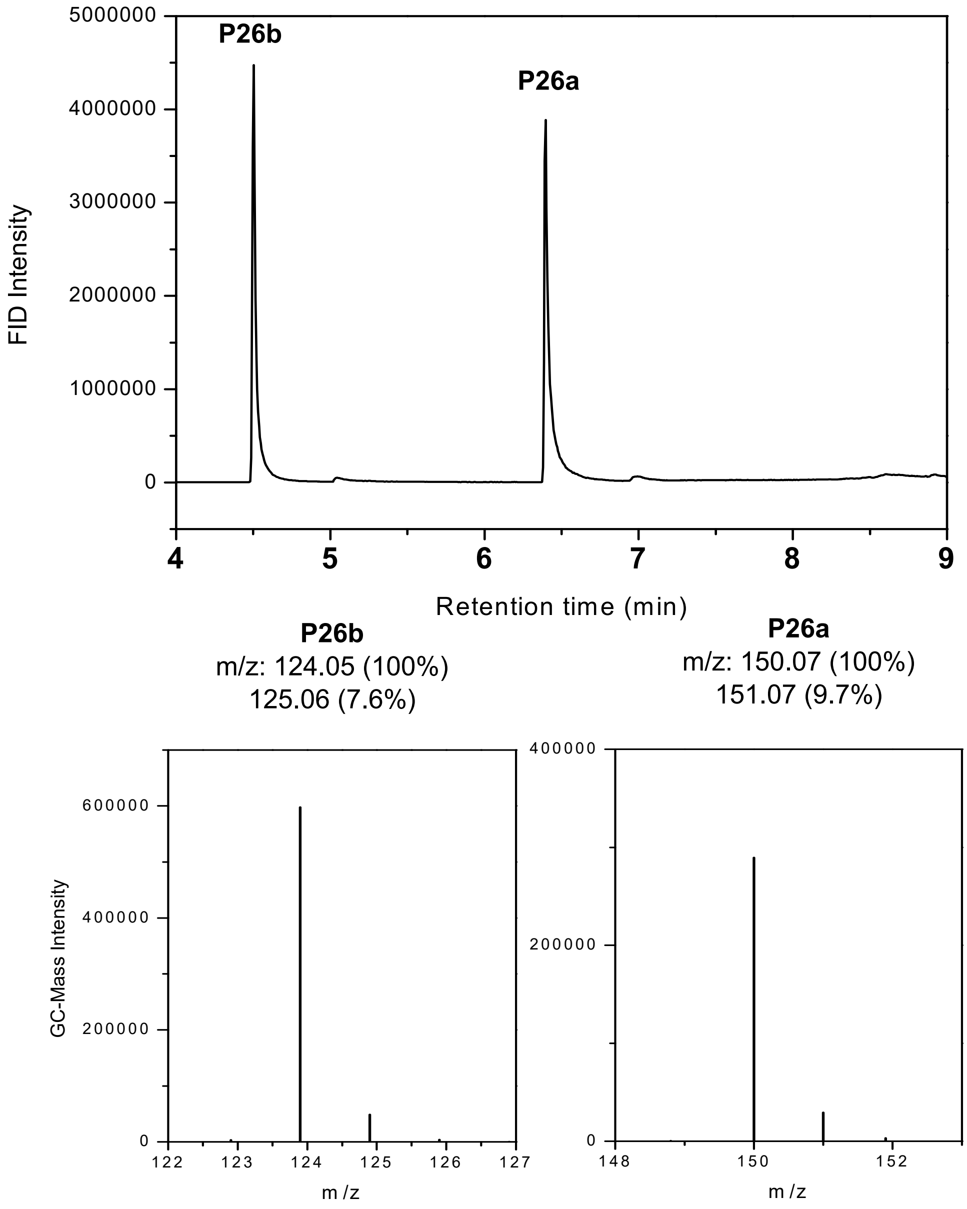

Figure S24. GC-FID and GC-MS data for the cleavage of **S26** under photoredox catalysis.

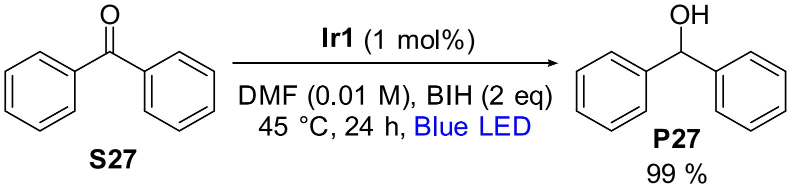

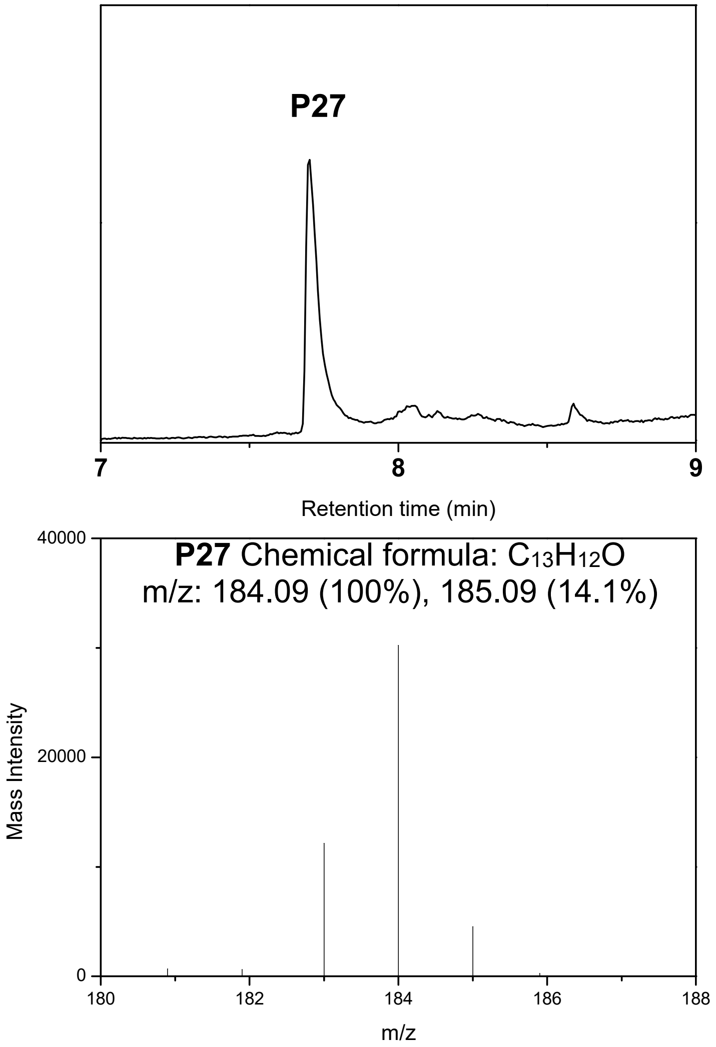

Figure S25. GC-FID (top) and GC-MS (bottom) data for the reduction of **S27** under photoredox catalysis.

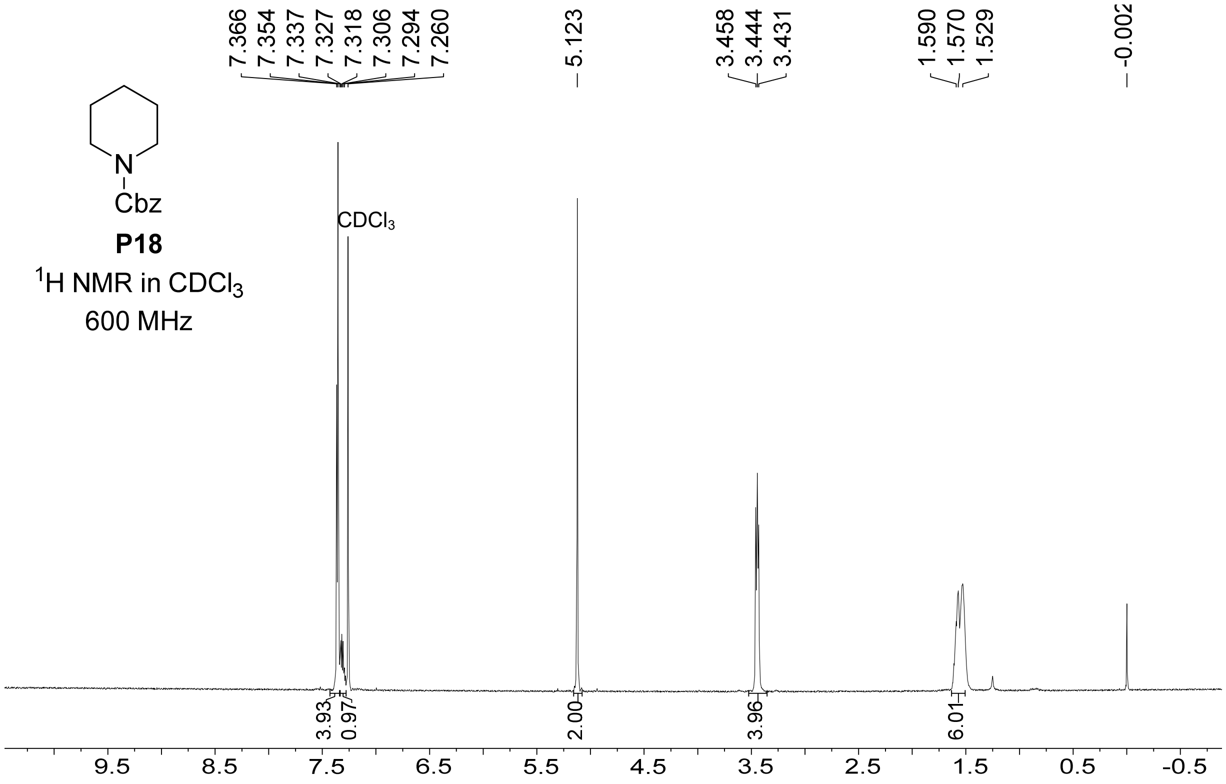

Figure S26. ^{1}H NMR spectrum of the isolated product **P18**.

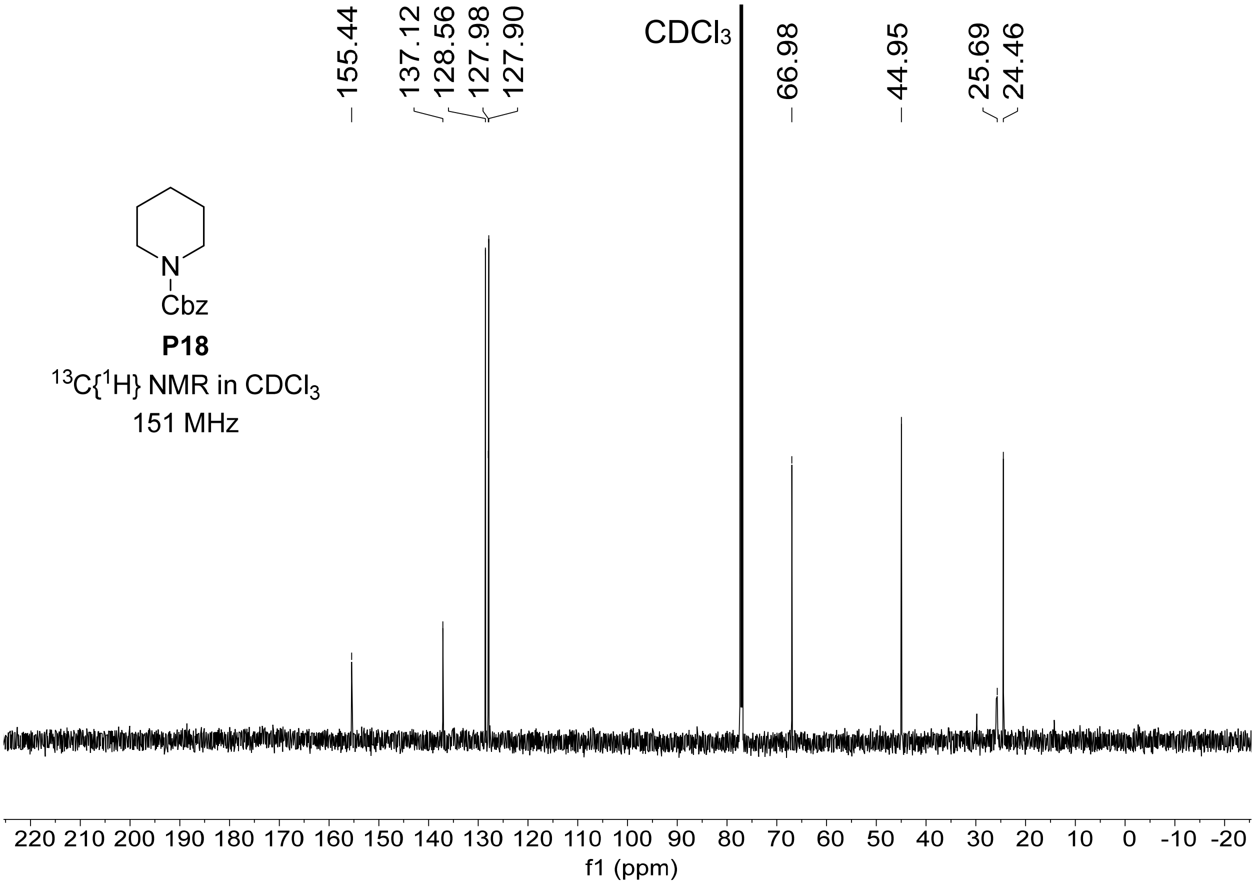

Figure S27. $^{13}C\{^{1}H\}$ NMR spectrum of the isolated product **P19**.

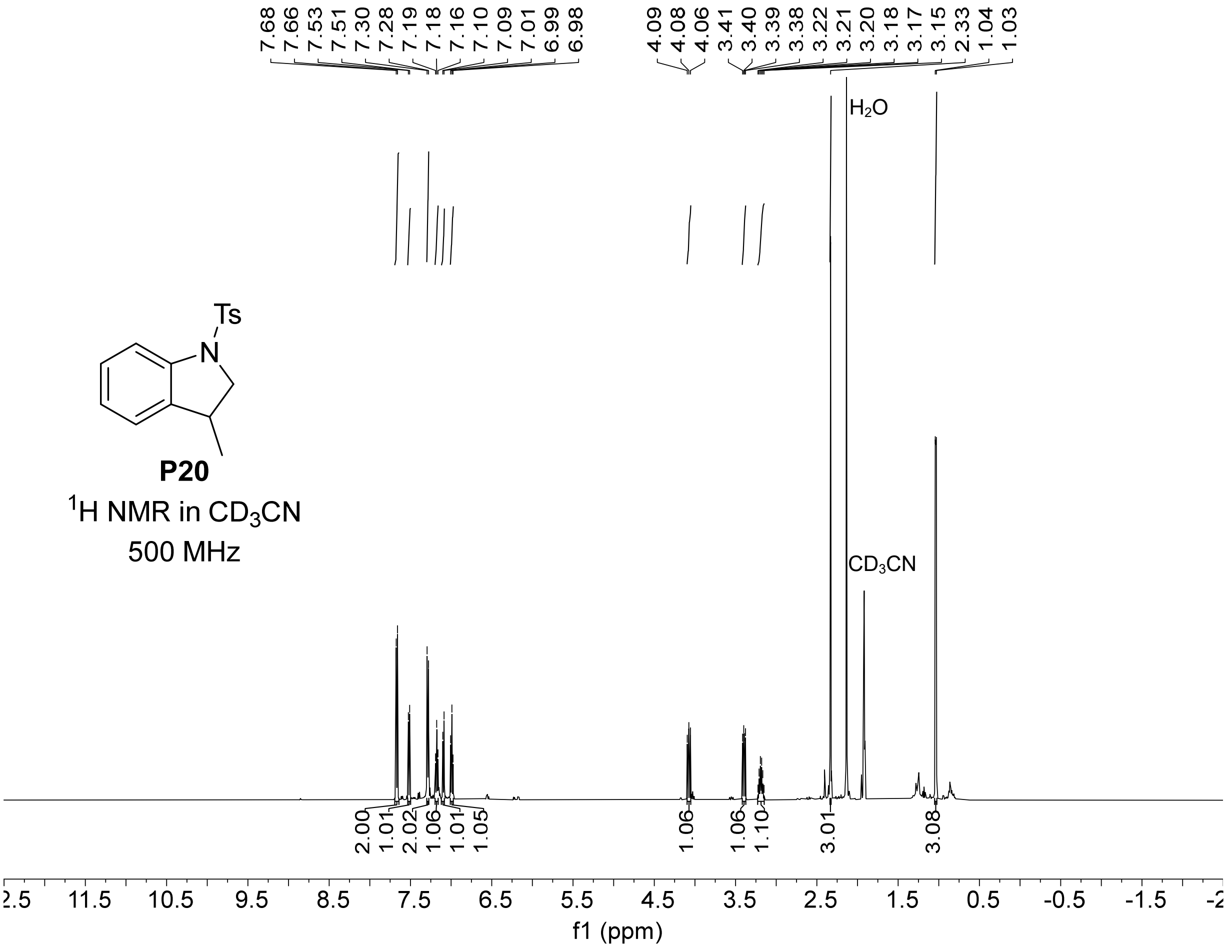

Figure S28. ^{1}H NMR spectrum of the isolated product **P20.**

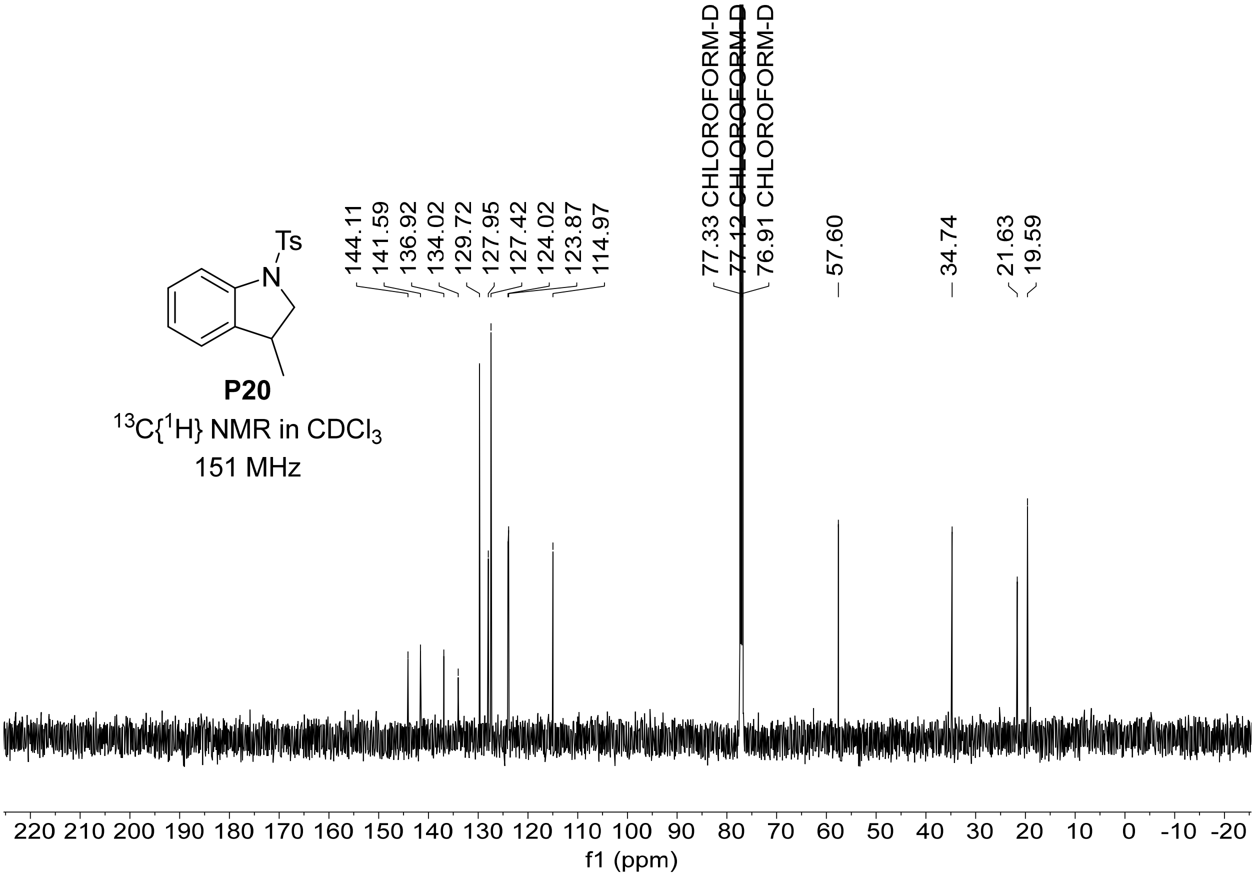

Figure S29. $^{13}C\{^{1}H\}$ NMR spectrum of the isolated product **P20**.

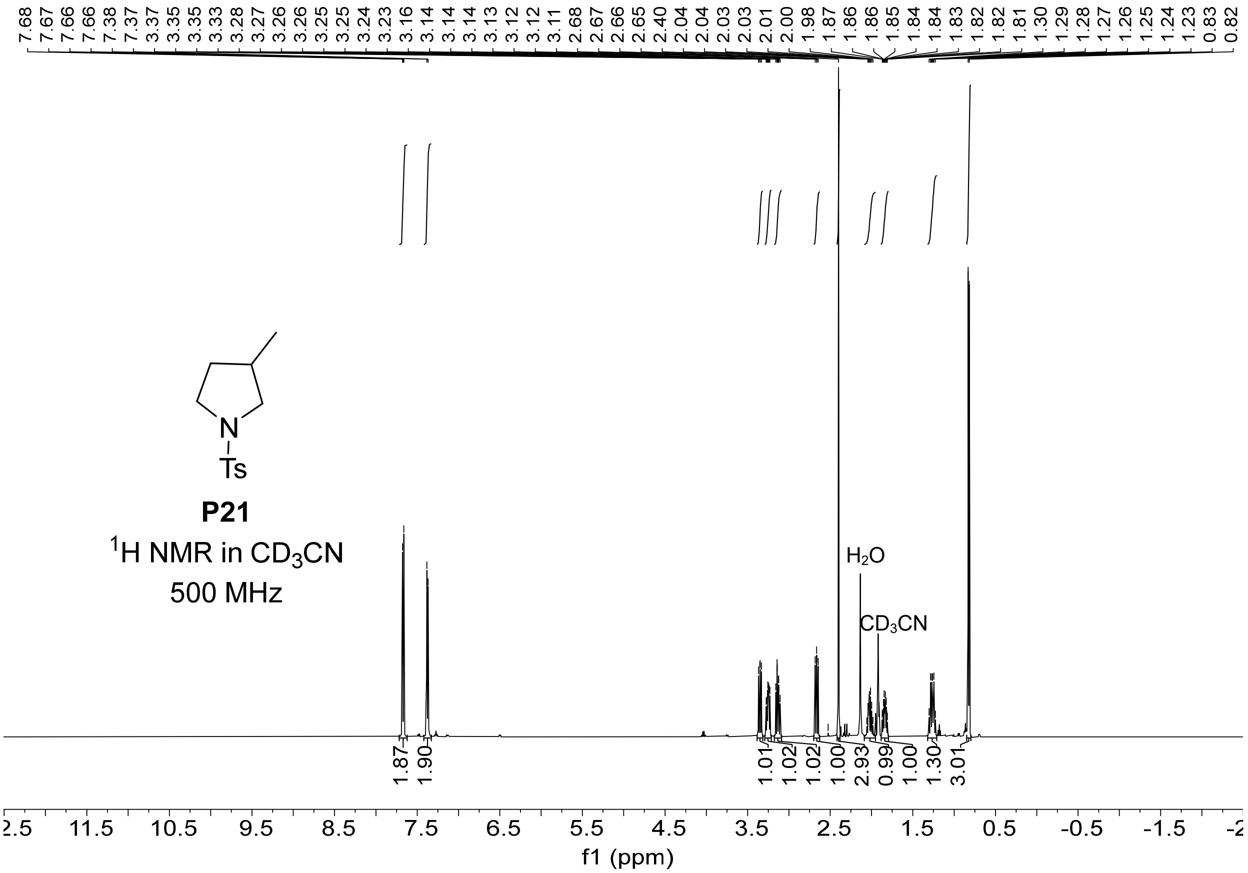

Figure S30. ^{1}H NMR spectrum of the isolated product **P21**.

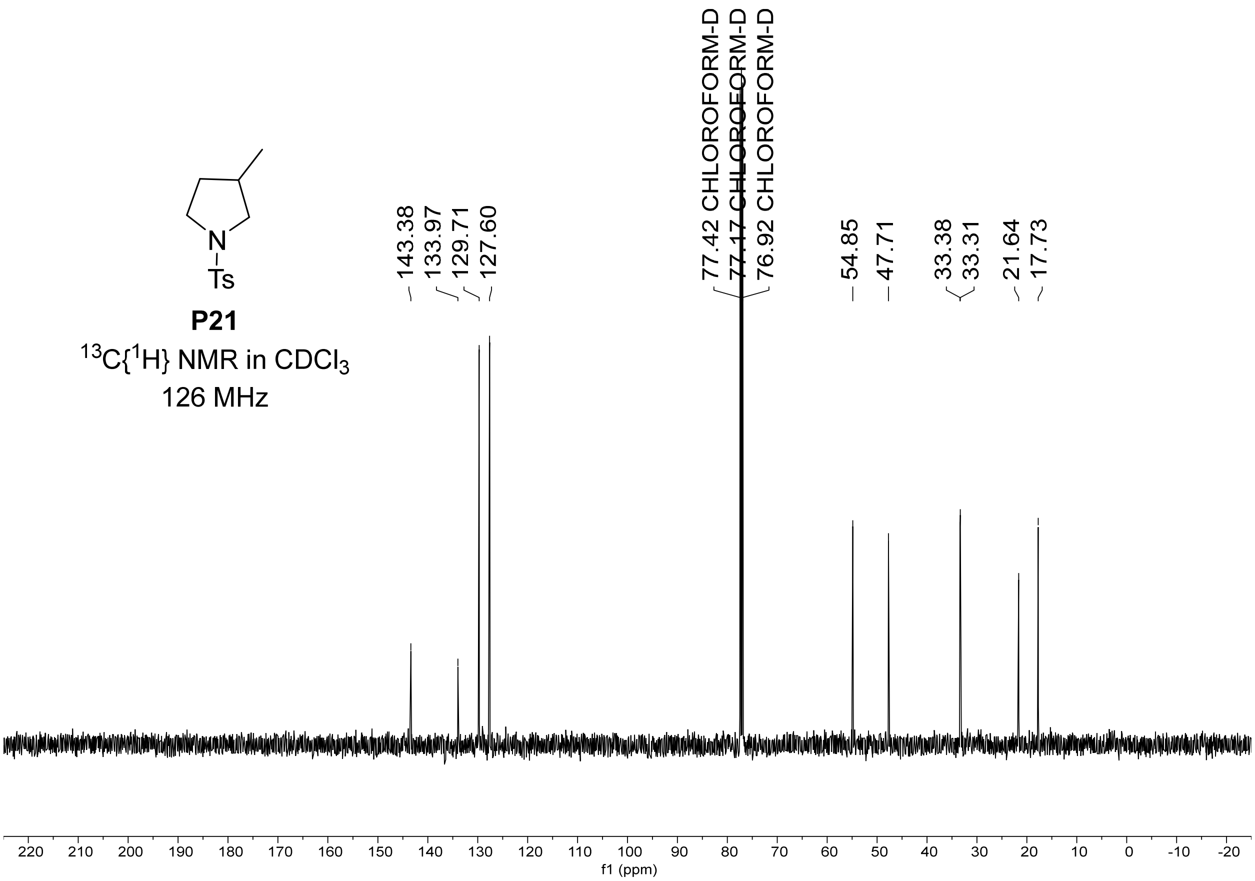

Figure S31. $^{13}C\{^{1}H\}$ NMR spectrum of the isolated product **P21**.

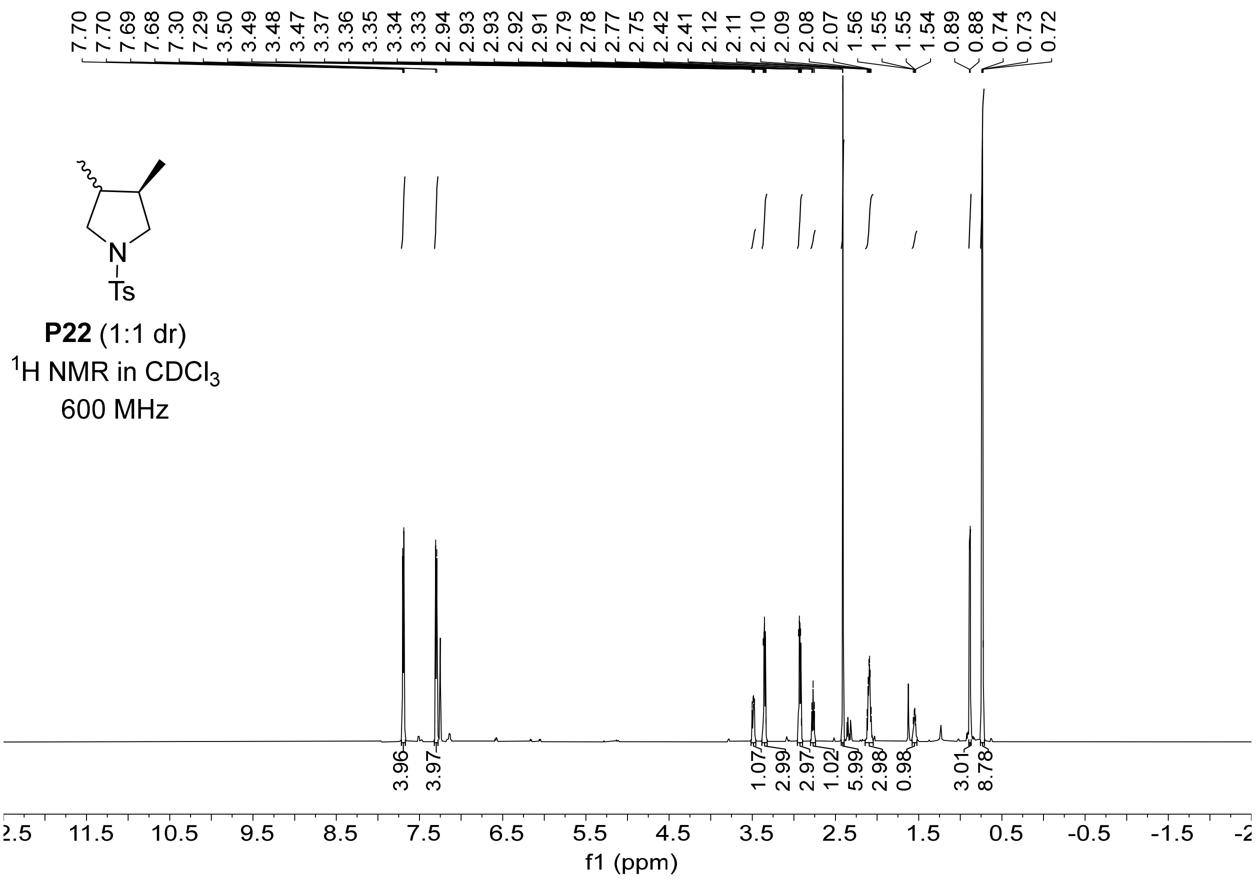

Figure S32. ^{1}H NMR spectrum of the isolated product **P22**.

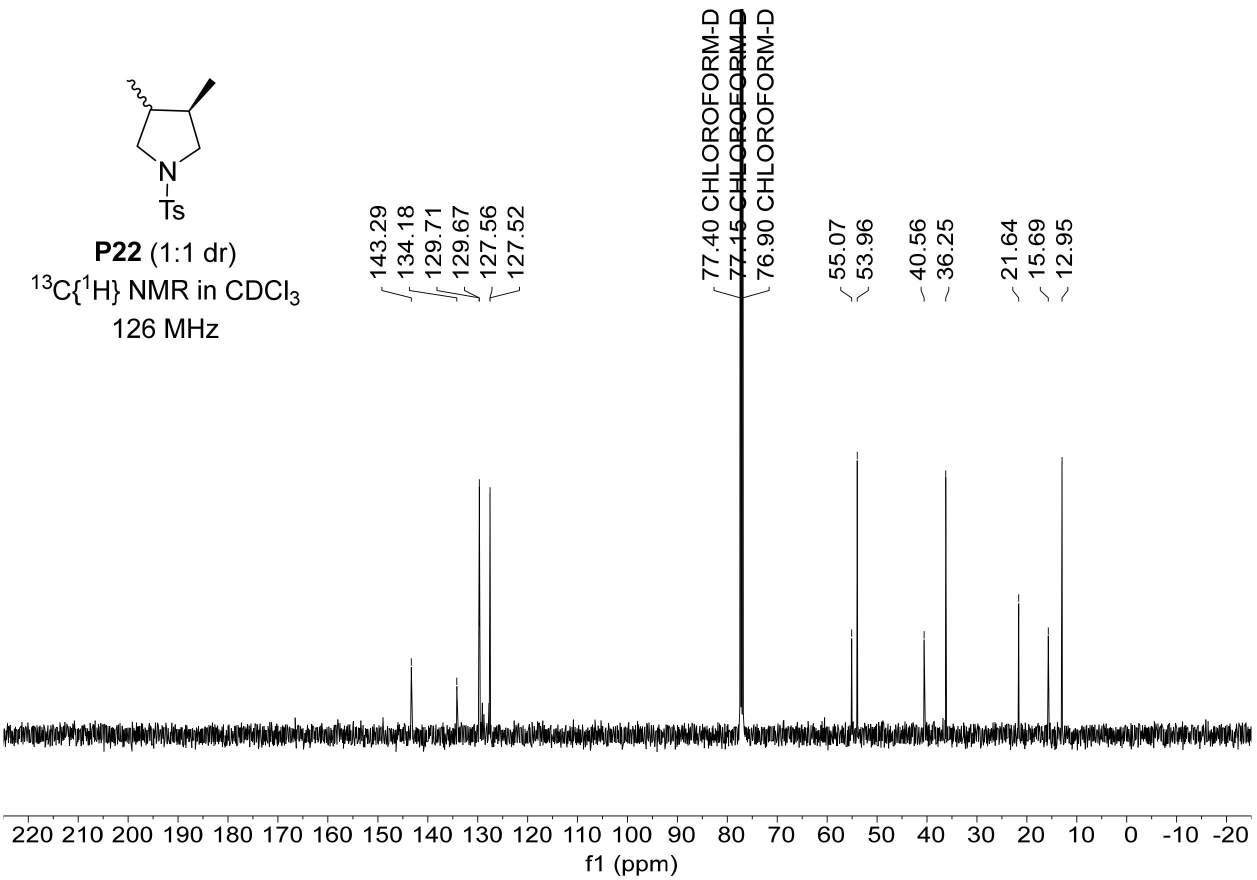

Figure S33. $^{13}C\{^{1}H\}$ NMR spectrum of the isolated product **P22**.

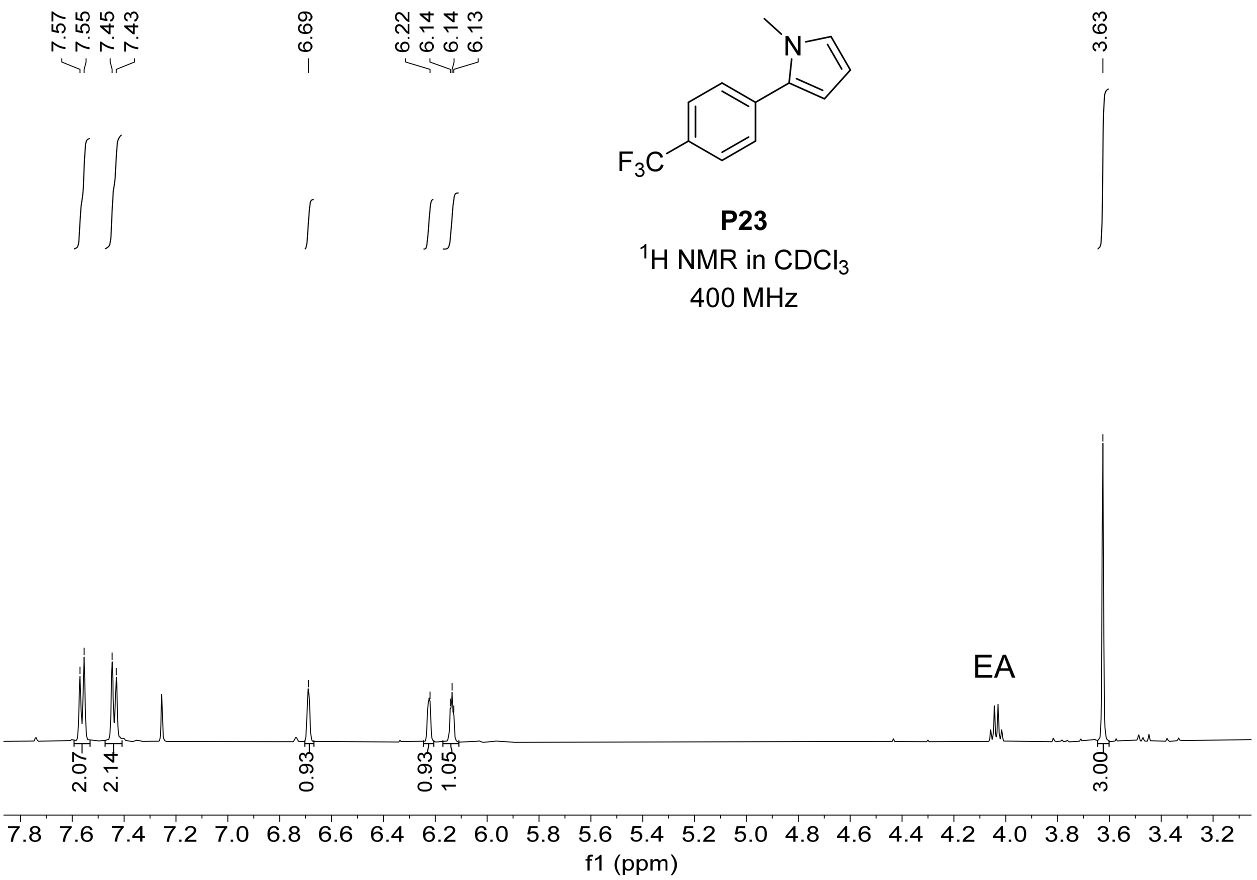

Figure S34. ^{1}H NMR spectrum of the isolated product **P23** (EA = ethyl acetate).

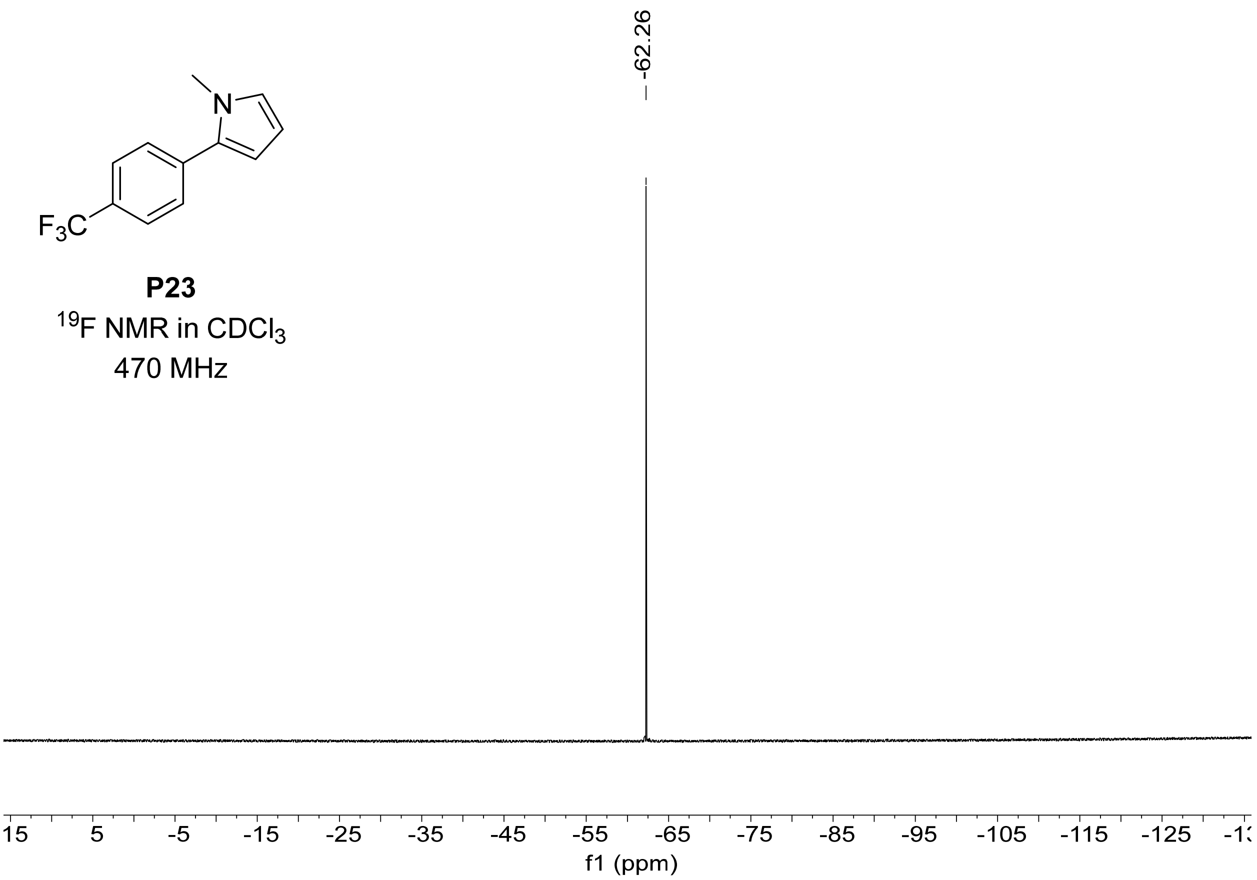

Figure S35. ^{19}F NMR spectrum of the isolated product **P23**.

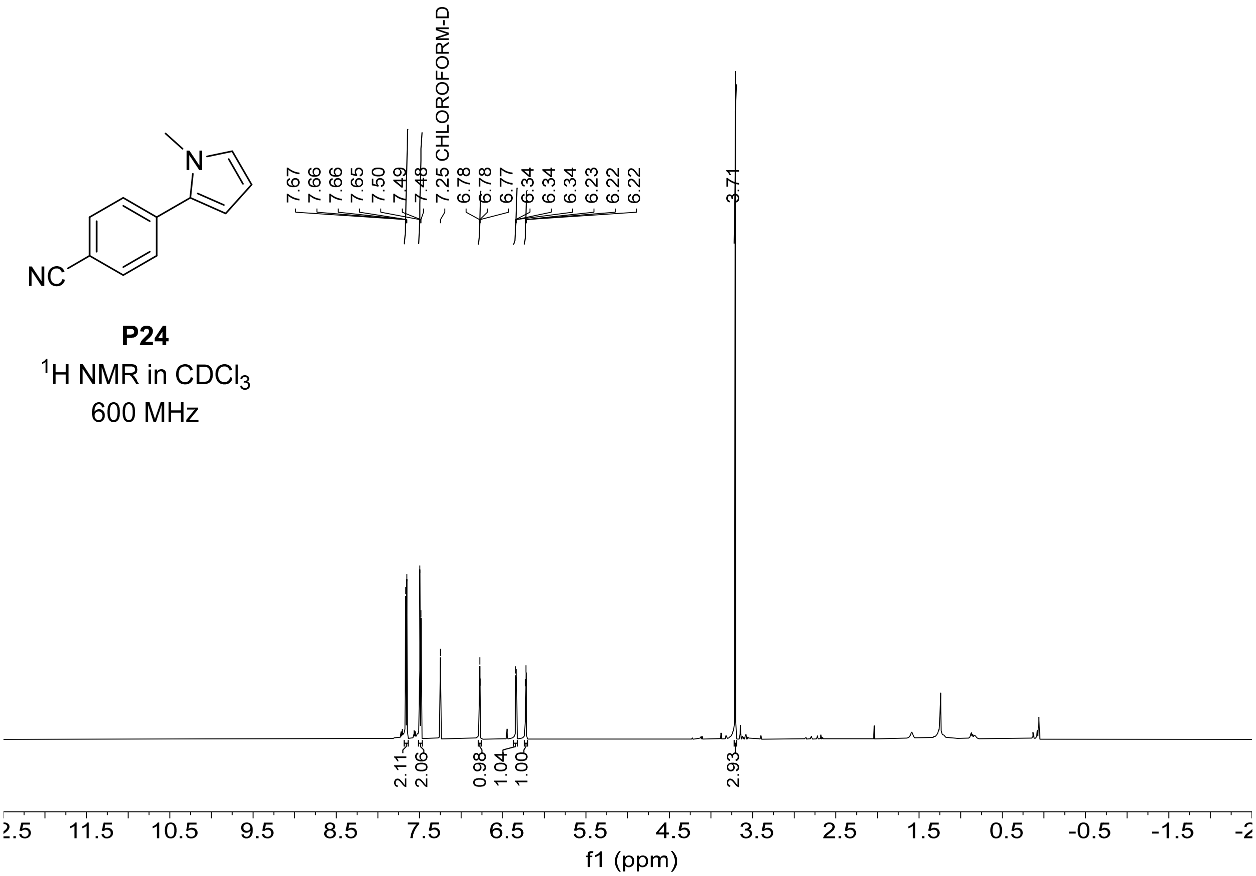

Figure S36. ^{1}H NMR spectrum of the isolated product **P24.**

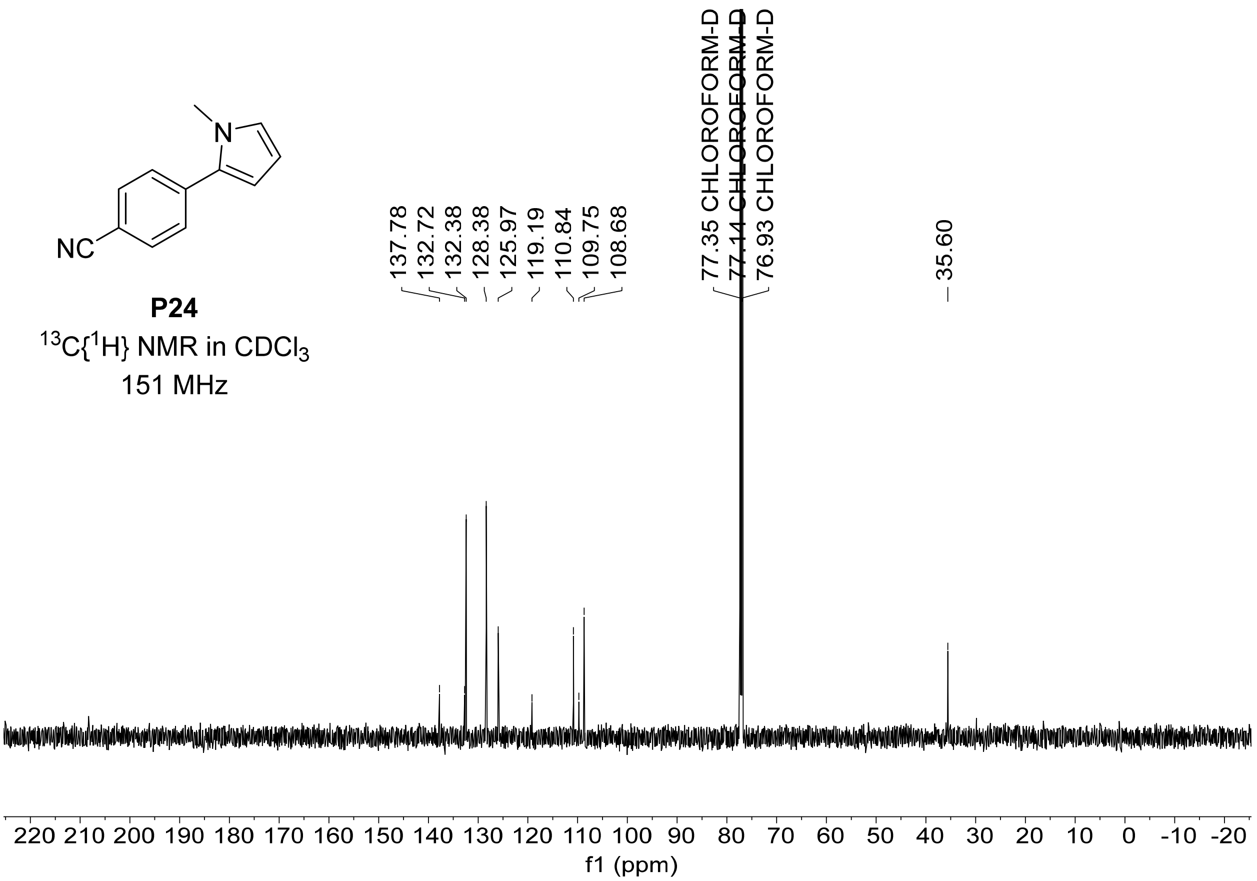

Figure S37. ^{13}C NMR spectrum of the isolated product (**P24**).

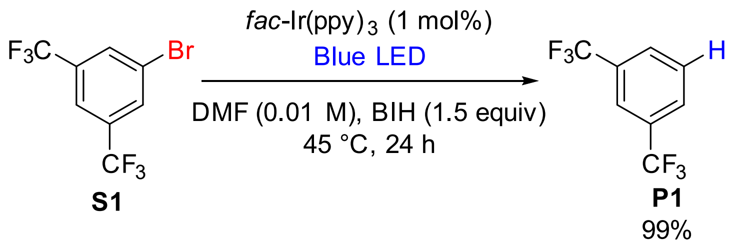

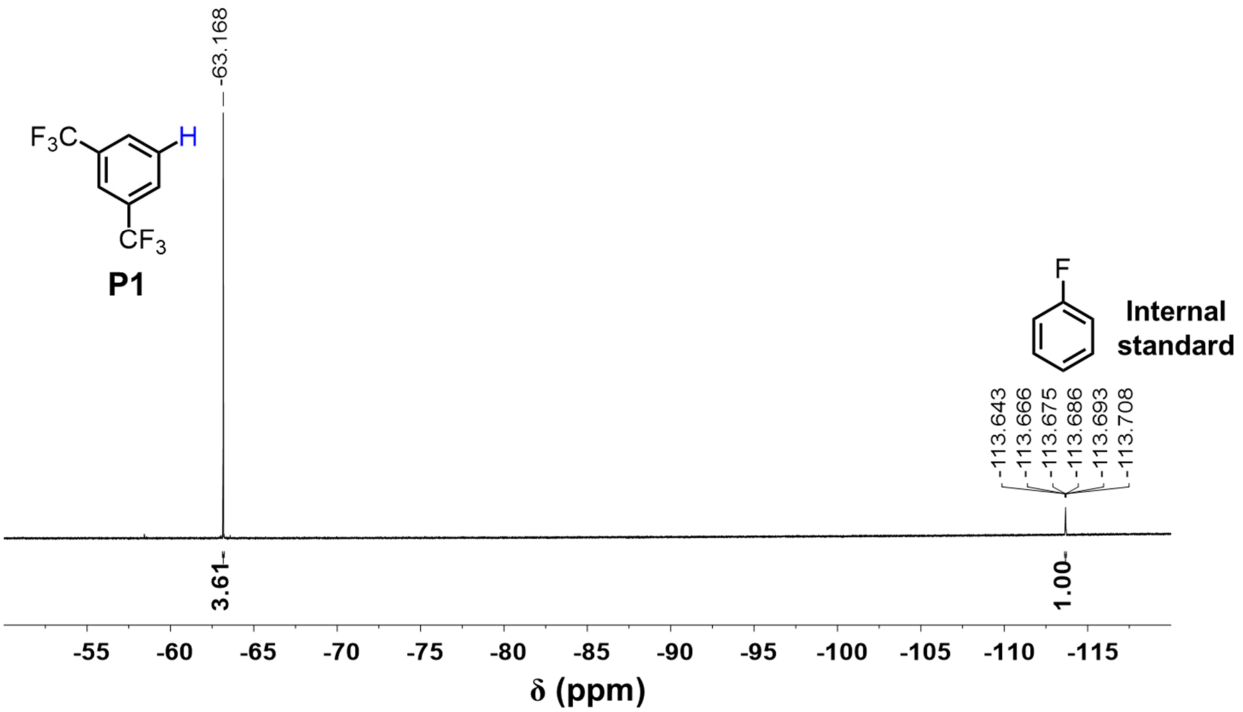

Figure S38. ^{19}F NMR spectrum for the hydrodebromination of **S1** using *fac*-Ir(ppy)3 as the photocatalyst.

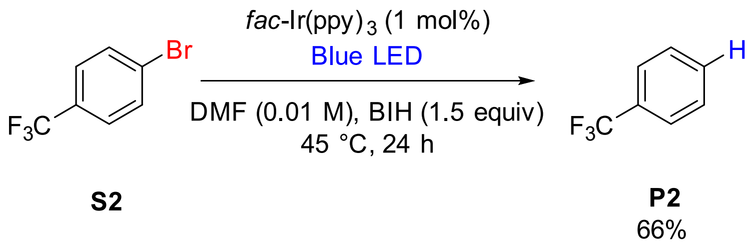

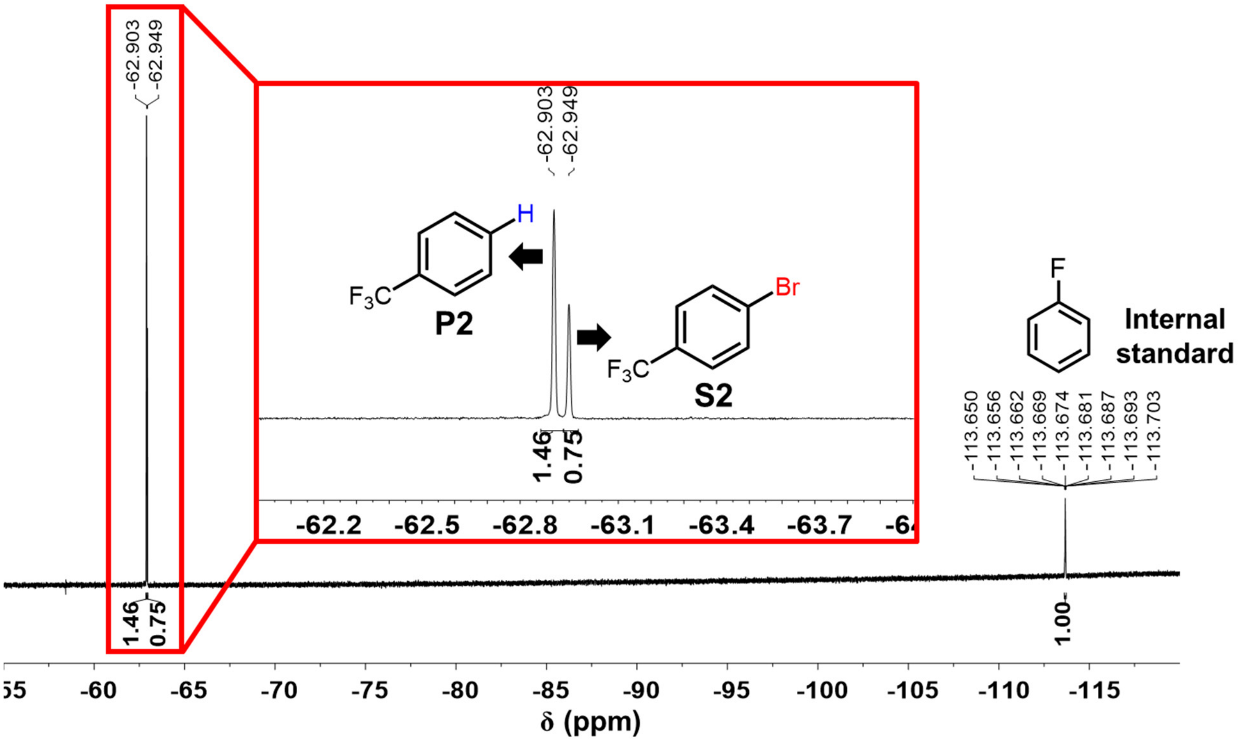

Figure S39. ^{19}F NMR spectrum for the hydrodebromination of **S2** using *fac*-Ir(ppy)$_3$ as the photocatalyst**.**

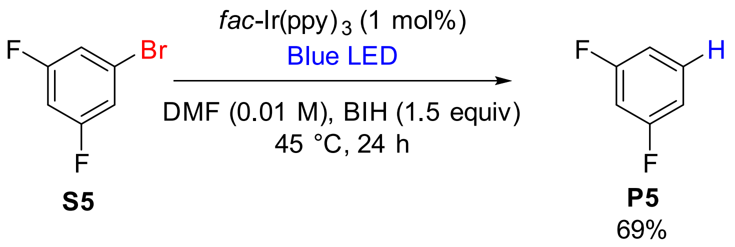

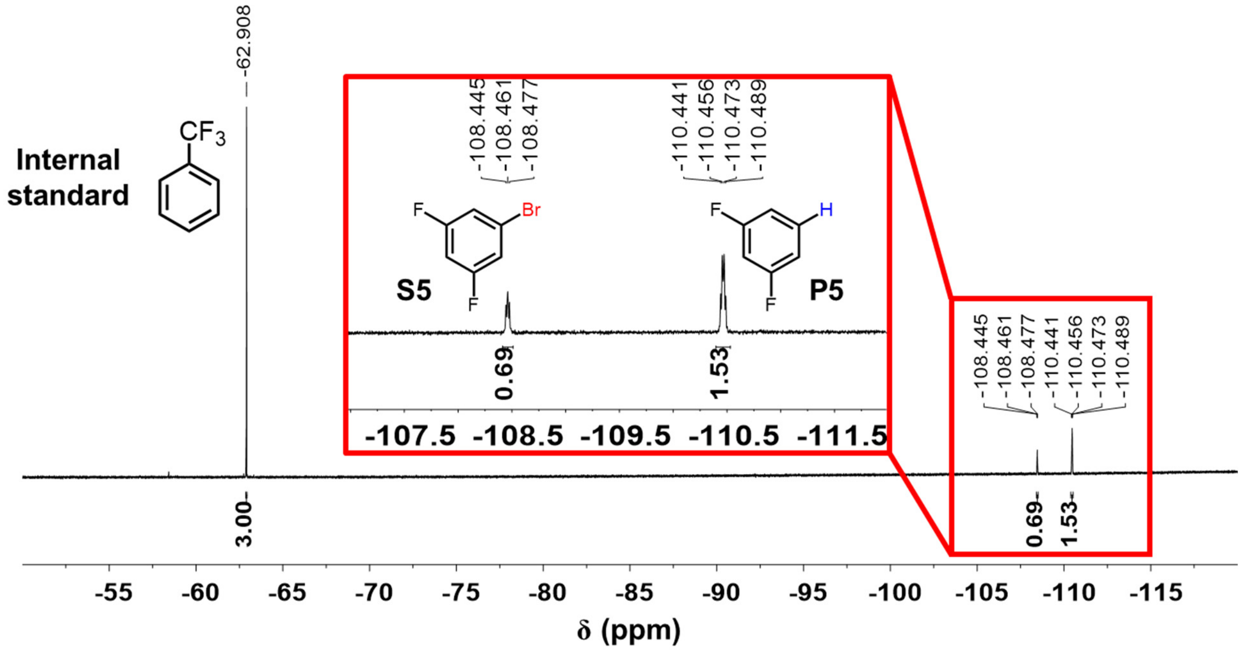

Figure S40. ^{19}F NMR spectrum for the hydrodebromination of **S5** using *fac*-Ir(ppy)3 as the photocatalyst**.**

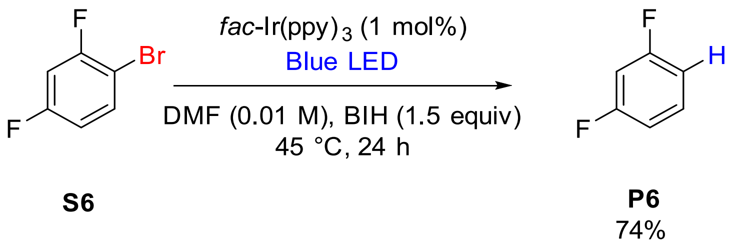

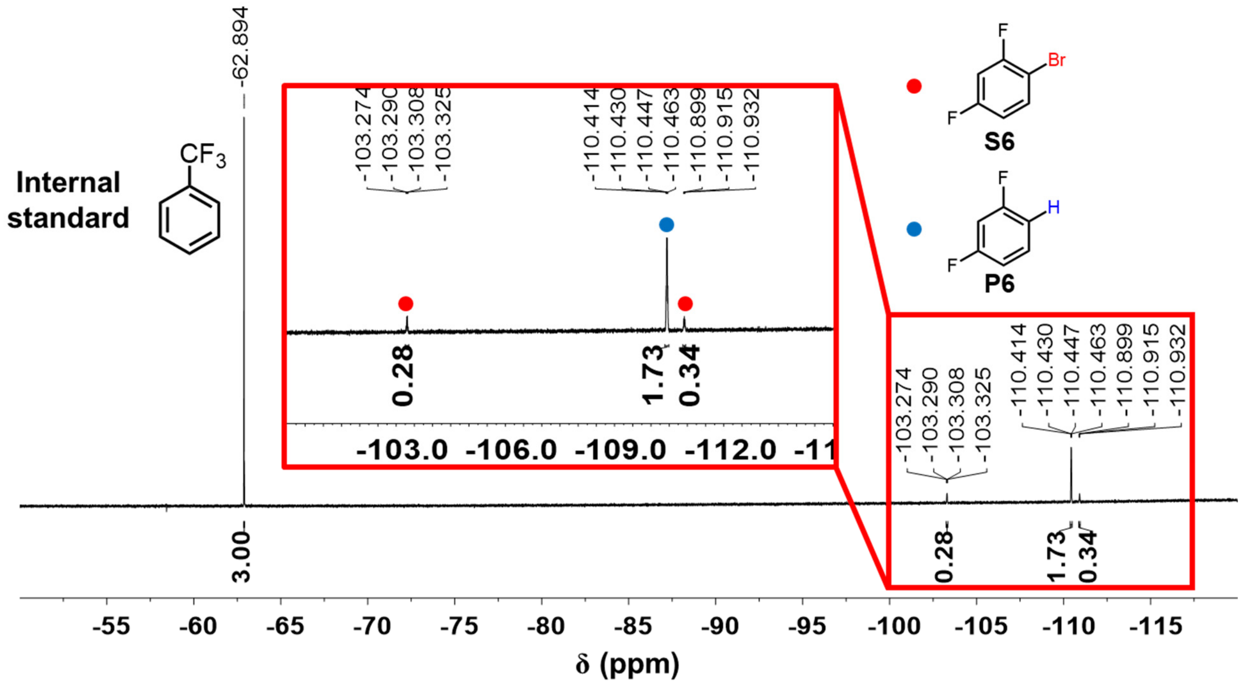

Figure S41. ^{19}F NMR spectrum for the hydrodebromination of **S6** using *fac*-Ir(ppy)₃ as the photocatalyst.

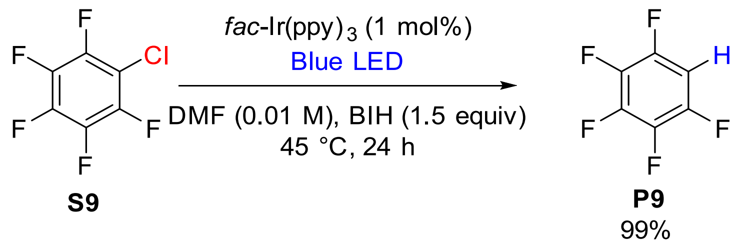

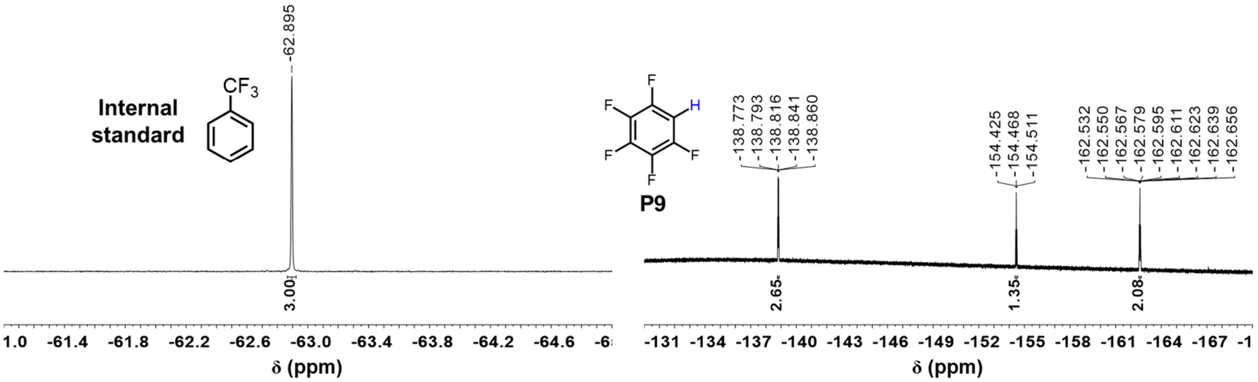

Figure S42. ^{19}F NMR spectrum for the hydrodechlorination of **S9** using *fac*-Ir(ppy)3 as the photocatalyst**.**

ESI References

(1) Shon, J.-H.; Teets, T. S. Potent Bis-Cyclometalated Iridium Photoreductants with β-Diketiminate Ancillary Ligands. *Inorg. Chem.* **2017**, *56* (24), 15295–15303. https://doi.org/10.1021/acs.inorgchem.7b02859.

(2) Shon, J.-H.; Sittel, S.; Teets, T. S. Synthesis and Characterization of Strong Cyclometalated Iridium Photoreductants for Application in Photocatalytic Aryl Bromide Hydrodebromination. *ACS Catal.* **2019**, 8646–8658. https://doi.org/10.1021/acscatal.9b02759.

(3) Dedeian, K.; Djurovich, P. I.; Garces, F. O.; Carlson, G.; Watts, R. J. A New Synthetic Route to the Preparation of a Series of Strong Photoreducing Agents: *Fac*-Tris-Ortho-Metalated Complexes of Iridium(III) with Substituted 2-Phenylpyridines. *Inorg. Chem.* **1991**, *30* (8), 1685–1687. https://doi.org/10.1021/ic00008a003.

(4) Hasegawa, E.; Izumiya, N.; Miura, T.; Ikoma, T.; Iwamoto, H.; Takizawa, S.; Murata, S. Benzimidazolium Naphthoxide Betaine Is a Visible Light Promoted Organic Photoredox Catalyst. *J. Org. Chem.* **2018**, *83* (7), 3921–3927. https://doi.org/10.1021/acs.joc.8b00282.

(5) Rosas-Hernández, A.; Steinlechner, C.; Junge, H.; Beller, M. Earth-Abundant Photocatalytic Systems for the Visible-Light-Driven Reduction of CO_2 to CO. *Green Chem.* **2017**, *19* (10), 2356–2360. https://doi.org/10.1039/C6GC03527B.

(6) Revol, G.; McCallum, T.; Morin, M.; Gagosz, F.; Barriault, L. Photoredox Transformations with Dimeric Gold Complexes. *Angew. Chem. Int. Ed.* **2013**, *52* (50), 13342–13345. https://doi.org/10.1002/anie.201306727.

(7) Devery, J. J.; Nguyen, J. D.; Dai, C.; Stephenson, C. R. J. Light-Mediated Reductive Debromination of Unactivated Alkyl and Aryl Bromides. *ACS Catal.* **2016**, *6* (9), 5962–5967. https://doi.org/10.1021/acscatal.6b01914.

(8) Sequeira, F. C.; Chemler, S. R. Stereoselective Synthesis of Morpholines via Copper-Promoted Oxyamination of Alkenes. *Org. Lett.* **2012**, *14* (17), 4482–4485. https://doi.org/10.1021/ol301984b.

(9) Liu, H.; Li, H.; Lu, J.; Zeng, S.; Wang, M.; Luo, N.; Xu, S.; Wang, F. Photocatalytic Cleavage of C–C Bond in Lignin Models under Visible Light on Mesoporous Graphitic Carbon Nitride through π–π Stacking Interaction. *ACS Catal.* **2018**, *8* (6), 4761–4771. https://doi.org/10.1021/acscatal.8b00022.

(10) Senaweera, S. M.; Singh, A.; Weaver, J. D. Photocatalytic Hydrodefluorination: Facile Access to Partially Fluorinated Aromatics. *J. Am. Chem. Soc.* **2014**, *136* (8), 3002–3005. https://doi.org/10.1021/ja500031m.

(11) Hofmann, J.; Gans, E.; Clark, T.; Heinrich, M. R. Radical Arylation of Anilines and Pyrroles via Aryldiazotates. *Chem. – Eur. J.* **2017**, *23* (40), 9647–9656. https://doi.org/10.1002/chem.201701429.

(11) Gowrisankar, S.; Seayad, J. AgONO-Assisted Direct C–H Arylation of Heteroarenes with Anilines. *Chem. - Eur. J.* **2014**, *20* (40), 12754–12758. https://doi.org/10.1002/chem.201403640.

(13) Priya, S.; Weaver, J. D. Prenyl Praxis: A Method for Direct Photocatalytic Defluoroprenylation. *J. Am. Chem. Soc.* **2018**, *140* (47), 16020–16025. https://doi.org/10.1021/jacs.8b09156.

(14) *Handbook of Photochemistry*, 3rd ed.; Montalti, M., Murov, S. L., Eds.; CRC/Taylor & Francis: Boca Raton, 2006.
